# Protein Multifunctionality: Principles and Mechanisms

**Published:** 2008-05-15

**Authors:** Joseph Z. Zaretsky, Daniel H. Wreschner

**Affiliations:** Department Cell Research and Immunology, George Wise Faculty of Life Sciences, Tel-Aviv University, Ramat-Aviv, Haim Levanon St., 69978 Tel-Aviv, Israel

**Keywords:** protein, structure, function, multifunctionality

## Abstract

In the review, the nature of protein multifunctionality is analyzed. In the first part of the review the principles of structural/functional organization of protein are discussed. In the second part, the main mechanisms involved in development of multiple functions on a single gene product(s) are analyzed. The last part represents a number of examples showing that multifunctionality is a basic feature of biologically active proteins.

## I. Main Principles of Protein Organization: Structural Compactness and Functional Variability

### 1. General consideration on the factors determining protein multifunctionality

The cell fulfills two main functions: (1) storage of genetic information and transfer it to the next cell generation; (2) synthesis of different molecules, protein molecules in particular, for fulfillment of numerous cell processes.

The compactness (maximum information packed in minimum DNA space) is one of the main principles of genetic information storage. The fulfillment of the first cell function is based on this principle. On the contrary, the abundance, variability and diversity of the protein molecules are necessary for realization of the second function. In contrast to DNA, proteins exhibit an apparently unlimited variety of structure. This is a necessary requirement of the vast array of different functions that they perform in maintenance of life “in contrast to the relatively static archival function of DNA” (Lesk, 2001). In the protein world, not only “a bewildering variety of form is observed but even within a common structure, there is variation in the lengths and orientation of substructures. Such variation is both a reflection of the very long evolution of protein structures and also a consequence of the fact that proteins cannot be completely rigid bodies but must have flexibility to accommodate the structural changes that are almost always necessary for them to perform their functions” (Lesk, 2001).

All multiple variations in protein structures and functions are genetically determined by DNA sequence fixed in amino acid primary structure of a protein and can be achieved by different mechanisms operating at different levels.

**Figure 1:**
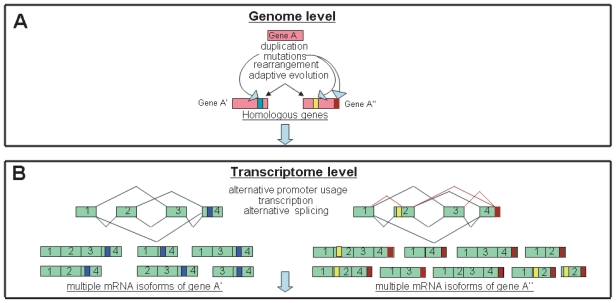

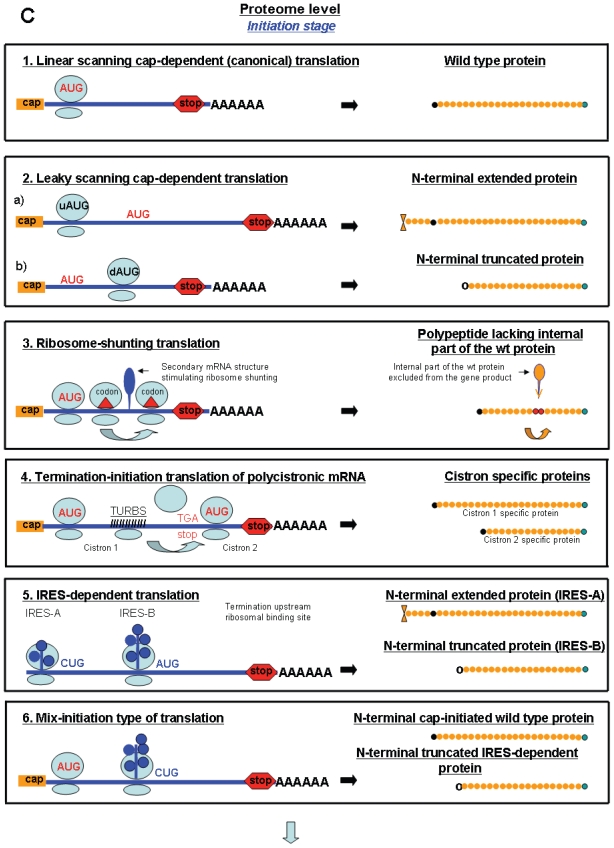

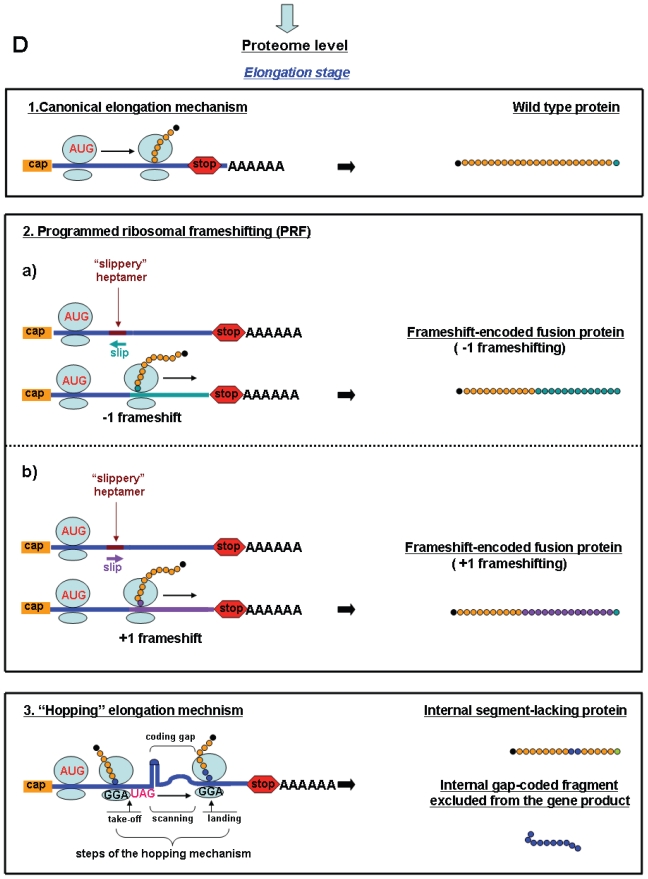

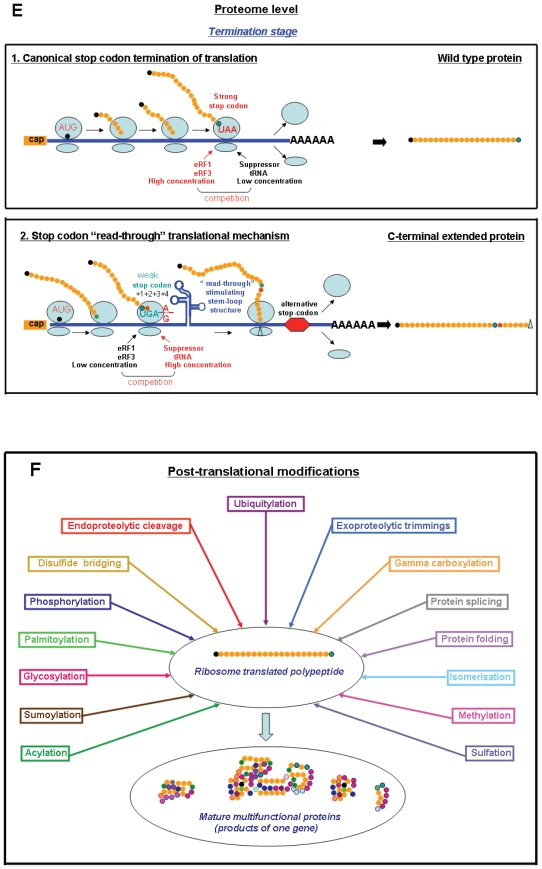
Mechanisms involved in development of protein multifunctionality. Mechanisms operating at genome (**A**), transcriptome (**B**), proteome (**C–E**) and post-translational (**F**) levels.

At the level of *genome*, a single gene may undergo duplication followed by rearrangement and/or mutations thus leading to appearance of homologous proteins possessing partially similar and/or different functions (Fig. 1, A).

At the next level of variability and complexity, *transcriptome* level, the gene transcription can be altered by several mechanisms including usage of alternative promoters, different transcription initiation sites, mRNA splicing and mRNA stability (Fig. 1, B). The extent of variability that may be obtained by mRNA alternative splicing is almost unlimited. An exelent example of this statement is the Drosophila gene Dscam, encoding Down syndrome cell-adhesion protein. Althernative splicing of Dscam pre-mRNA potentially generates 38 016 isoforms of a cell-surface recognition protein (Schmucker et al. 2000; Zipursky et al. 2006).

Further complexity is observed at the *proteome* level. A variety of the protein products encoded by the same gene can be achieved by three groups of specific mechanisms: those operating at the translational level; the mechanisms that determine post-tranlational modifications and mechanisms controlling the intermolecular interactions. Multiple translation products can be synthesized by usage different cap-dependent and cap-independent internal ribosome entry site (IRES) determined initiation mechanisms (Fig. 1, C) including leaky scanning, ribosome-shunting, termination-initiation and mix (or alternative) initiation (Cai et al. 2006; Candeias et al. 2006; Holcik et al. 2000; Huez et al. 1998; Kochetov, 2006; Kozak, 2002; Lu et al. 2004; Yueh and Schneider, 1996). At elongation stage of protein synthesis, frameshifting and “hopping” (Fig. 1, D) also make substantial impact in protein diversity (Atkins et al. 2001; Herr et al. 2000b; Namy et al. 2004). Stop codon readthrough (Fig. 1, E) is the main mechanism that determines appearance of new protein isoforms at the last stage of translation (Keeling et al. 2004; Namy et al. 2002).

Post-translation modifications of the primary polypeptide molecule (Fig. 1, F) include such mechanisms as different types of glycosylation, phosphorylation, acylation, specific restricted proteolysis, protein splicing and many others (Hanada et al. 2004; Rehfeld and Goetze, 2003). The intermolecular interactions that modify protein properties and functions occur between protein and metal, protein and carbohydrate, protein and lipid and protein and protein. These interactions lead to multiple folding variants which in their turns determine topology and functions of multiple products encoded by one and the same gene.

It is important to underline once again that all possible modifications and interactions of a given gene product(s) are encoded in its nucleotide sequence and, as a consequence, in amino acid structure of polypeptide molecule(s). The reactions described above are based on the natural mechanisms by which condensed genetic information can be transformed into variable and multiple protein structures and functions.

The wisdom of Nature [G-d] is that it does not create anything without specific purpose. There is nothing in nature what is superfluous or unnecessary. This is the main principle of nature. If amino acid sequence of a protein contains genetically determined possibilities for any type of modifications (by proteolysis, folding, glycosylation, phosphorylation etc), there should be definite conditions in a definite cell or tissue in an appropriate point of time which would allow realization of the encoded modifications.

Although the principle of structural and functional compactness is the most evident in nucleic acids, it would be mistake to think that this principle does not work in protein molecules. Actually, the genetic information contained in the DNA sequence is realized in two ways: each protein molecule produced by a gene contains maximum of functional possibilities, and each gene produces as much protein molecules as necessary for performing all functions encoded by the gene. These principles lead to multifunctionality of protein molecules. The following example from the virus world may illustrate this idea. Owing to the small size of their genome, viruses have evoleved by packing a maximum of genetic information in a minimum of polypeptide sequence. As a consequence, many of the proteins or protein domains encoded by viruses are multifunctional. The transmembrane (TM) domains of Hepatitis C Virus envelope glycoprotein are extreme examples of such multifunctionality. Indeed, these TM domains bear ER retention signals, demonstrate signal function and are involved in E1:E2 heterodimerization (Cocquerel et al. 1999; Cocquerel et al. 1998; Cocquerel et al. 2000). All these functions are partially overlapped and present in the sequence of <30 amino acids thus evidently demonstrating the principle of structure/functional compactness reflected in protein structure as well as in its functional map (Cocquerel et al. 2002).

### 2. Hierarchical nature of protein architecture

For better understanding ability of a protein to develope multiple functions and to discover potentials hidden in protein molecule, one should get insight the hierarchical nature of protein architecture. As pointed out by A. Lesk, several hierarhial levels of structural complexity can be observed in protein molecules (Lesk, 2001):

1. The first level is the sequence of amino acids in a polypeptide (*primary structure*).
2. The assignment of α-helices and β-sheets is the hydrogen-bonding pattern of the main polypeptide chain (*secondary structure*). This is the second level of complexity. Many proteins demonstrate recurrent patterns of interaction between helices and sheets present in one polypeptide molecule. These interactions form *supersecondary structure*.
3. The most of the protein molecules contain compact units within the folding patterns of a single chain, *domains,* that demonstrate definite structural stability and functional independence. Protein domains can be defined as segmented portions of a polypeptide sequence that assume stable three-dimensional structure (Richardson, 1981; Wriggers et al. 2005). A domain usually fulfills specific function(s). Assembly of domains and intervaining flexible stretches of short peptide chains develops multidomain protein molecule characterized by *the tertiary structure* (the third level of structural complexity).
4. Within multidomain proteins, each domain is capable of autonomous function, however, some of them also mediate the formation of complexes with partner proteins (intermolecular interactions) giving rise for new functions and forming so called a domain network (Santonico et al. 2005). The multidomain proteins containing more than one polypeptide chain compose the assembly of the monomers that represents the fourth level of complexity—*the quaternary structure*.

Multidomain proteins containing several copies of closely related domains are so called *modular proteins*. The modular protein can “mix and match” sets of domains. For example, fibronectin, a large extracellular protein, contains 29 domains including multiple tandem repeats of three types of domains, F1, F2 and F3 (Lesk, 2001). These and many other domains such as EGF, Ig, PZS etc are integral parts of the multiple modular proteins. Such recurring protein motifs, domains, are significant because it is increasingly recognized that “there are only a limited number of domain families in nature” (Wriggers et al. 2005). These domains are duplicated and combined in different ways to form the set of proteins (Apic et al. 2003).

Most of the known proteins are multifunctional molecules. The presence in a protein molecule of several domains that possess different functions indicates on its multifunctionality. At the beginning of the 1990s, K. Mizejewsky proposed the hypothesis of the “modular cassette” (Dauphinee and Mizejewski, 2002; Mizejewski, 1993). According to this hypothesis, the presence of amino acid stretches (domains) in a protein that are similar to those in physiologically active proteins implies that they may have similar functions. This hypothesis allows prediction of functional activities of newly discovered proteins or proteins which function have not yet been well studied. For example, more than 20 types of functionally important sites (domains) have been predicted and later on identified in α-fetoprotein (Terentiev and Moldogazieva, 2006). Among these sites, there are sites responsible for binding of hydrophobic ligands (metal ions, estrogens, bilirubin, retinoids, flavonoids, exotoxins, various dyes and drugs (Aoyagi et al. 1979; Aussel and Negrel, 1986; Birkenmeier et al. 1983; Iturralde et al. 1991; Milligan et al. 1998; Nagai et al. 1982; Nishi et al. 1991; Ruiz-Gutierrez et al. 2001; Ruoslahti et al. 1979; Tatarinov et al. 1991), sites that demonstrate immunosuppressive activity (Naval et al. 1985; Suzuki et al. 1992; Torres et al. 1992; Torres et al. 1989) and sites that regulate cell proliferation and tumor growth (Allen et al. 1993; Jacobson et al. 1990; Leffert and Sell, 1974; Li et al. 2002; Mizejewski et al. 1983).

It has become clear over the past decade that unrelated modular proteins frequently share significant portions of sequence similarity (Bork et al. 1997; Cesareni, 2004; Pawson and Nash, 2003; Santonico et al. 2005). Thus, a large number of functionally diverse proteins can be thought of as molecules built by combining a limited number of structurally stable folded domains. A good example of such “building bricks” is the *SAM* domains. The SMART database identifies more than 1300 *SAM*-containing proteins in genomes of all organisms from yeast to humans. They present in such different proteins as protein-kinases, lipid-kinases, scaffolding proteins, RNA-binding proteins and transcription factors. Although *SAM* domains adopt similar folds, they are remarkably versatile in their binding properties. Some identical *SAM* domains can interact with each other to form homodimers or polymers. In other cases, *SAM* domains can bind to other related *SAM* domains, to non-*SAM* domain-containing proteins, and even to RNA. Such versatility earns them multiple functional roles in different biological processes such as signal transduction, transcriptional regulation and translational control (Qiao and Bowie, 2005).

Speaking about protein architecture and function, it is important to underline the role which flexible linkers play in interconnection of the various domains in multidomain proteins. These linkers are stretches of amino acid residues that establish communication between different domains and functional modules (Gokhale and Khosla, 2000). A number of examples (e.g. immunoglobulin, diphtheria toxin, tomato bushy stunt virus protein) established a clear relationship between linker peptides and the functional dynamics they enable. Conformational transitions in multiple identical or homologous proteins have been observed. This gained widespread acceptance to the concept of hinge-bending (Dobson, 1990), whereby the relative flexibility of short regions of the polypeptide chain allow significant movement of structural domains.

One important consequence of the flexibility afforded by soft peptide linkers is the ability of linked domains to move to and from close spatial proximity. The flexibility and hydrophilicity of the linker are important factors in preventing the disturbance of the domain functions, thereby imparting stability to the domains (Arai et al. 2004). A range of stability occurs depending on rigidity of the linker peptides. Soft linkers confer flexibility, whereas more rigid peptides may act to keep domains apart (Wilchek and Miron, 1974).

Due to their ability to break or form contacts among adjacent domains, soft linkers often facilitate essential catalytic events in the overall function of a protein, as seen, for instance, in the tomato bushy stunt virus proteins (Harrison, 1981) or in the NFκB (Henkel et al. 1992). The hinge region of the NFκB is flexible enough to bring “p50” and “SW16/ANK” repeat domains into contact to regulate intracellular transport of the transcription factor (Henkel et al. 1992).

The classical example of the flexible linker power to produce large number of diverse functional interactions using limited number of structural domains is calmodulin. Calmodulin contains four Ca^2+^-binding domains known as the EF-hand with highly conserved amino acid sequences in all eukaryotes (Ikura and Ames, 2006; Kretsinger and Nockolds, 1973). Calmodulin regulates numerous target proteins that are functionally and structurally diverse. It activates cyclic nucleotide phosphodi-esterase (Cheung, 1970; Gopinath and Vincenzi, 1977; Kakiuchi and Yamazaki, 1970), Ca^2+^-transpotring ATPase (Gopinath and Vincenzi, 1977; Jarrett and Penniston, 1977), phosphorylase kinase (Cohen et al. 1978), nitric oxide synthetase (Stuehr et al. 1991) and many others. The list of known calmodulin dependent proteins exceeds 300 in number (Yap et al. 2000).

How calmodulin could bind and regulate all these different proteins? The structure determined for calmodulin in the complex with a target protein by NMR showed remarkable conformational change in calmodulin’s two EF-hand domains upon binding to the target protein (Ikura et al. 1992; Meador et al. 1992; Yap et al. 2000). This structure revealed that central domain linker is magically flexible and can be bent dramatically upon binding to the target protein (Barbato et al. 1992; Persechini and Kretsinger, 1988; Trewhella, 1992). The flexibility of the linker enables calmodulin to change its conformation as needed depending on the structure of target protein. Such conformational plasticity of calmodulin protein allows it to gain multifunctionality: by simply connecting two EF-hand modules via a flexible linker, an exponential increase in the number of target interactions is achieved (Ikura and Ames, 2006).

## II. The Basic Mechanisms Ensureing Transformation of Protein Structural Pecularities into Functional Multiplicity

Protein structural complexity and compactness may be achieved by different mechanisms operating at genome, transcriptome and proteome levels (Fig. 1, A–E). Several examples of multifunctional proteins described below may demonstrate how multifunctionality may be developed by these mechanisms.

### Genome level

The main tools in generation of biological diversity at the genome level are mutations (insertion or deletion of nucleotides in DNA sequences), transposition of pieces of DNA and gene duplication followed by genetic divergencies in each duplicated genes. If these changes in genetic information induce changes in protein structure and function, they undergo evolutionary selection and can be traced phylogenically (Fig. 1, A).

#### 1. Mutations as sources for multifunctionality

The product of the mutated gene can be “either an alternative protein of equivalent function -a *neutral mutation*- or a protein that carries out the same function but with an altered rate or specificity profile. It can be a protein with an altered function, or a protein that does not function—or even fold- at all” (Lesk, 2001). For instance, human adult and foetal haemoglobins differ by substitution of Ser (143β) for His. Primarily, as a result of this mutation, foetal haemoglobin demonstrates lower affininty than adult homologue for the regulatory ligand diphosphoglycerate. This promotes the transfer of oxygen across the placenta to the foetus (Lesk, 2001).

Another example demonstrates role of minor genetic alterations in induction of fundamental functional changes in the homologous proteins. Haptoglobin is a chymotrypsin homologue. As a result of homologuos gene mutations, it lost proteolytic activity characteristic for chymotrypsin but obtained several other functions: haptoglobin may function as chaperon as well as factor preventing the loss of iron from erythrocytes and as protein mediating immune responses (Lesk, 2001).

#### 2. Gene duplication as the origin of genetic novelties

It has been proposed that gene duplication is one of the important step for the origin of genetic novelties (Gu et al. 2002; Ohno, 1970). The following examples can confirm this proposition. GroEL is a 60 Kda heat-shock protein ubiquitous in bacteria. Interestingly, the GroEL in *Chlamydiae* became duplicated at the origin of the *Chlamydiae* lineage presenting three distinct molecular chaperones, namely the original protein GroEL1, and its paralogous proteins GroEL2 and GroEL3 (Karunakaran et al. 2003; McNally and Fares, 2007). GroEL protein copies species have diverged functionally after the gene duplication events. The functional divergence has occurred in important functional regions of these proteins. Even though the three *Chlamidiae* GroEL proteins present substantial amino acid sequence conservation in important regions involved in polypeptide binding when compared to GroEL from the *Escherichia coli*, significant differences have been spotted in GroEL binding regions and at regions involved in ATP binding and hydrolysis. Most of the amino acid replacements that have affected interaction with protein partners and were responsible for the functional divergence between GroEL paralogs were fixed by adaptive evolution after the groEL gene duplication events (McNally and Fares, 2007).

Stromal interaction molecules (STIMs) represent another example demonstrating the role of gene duplication in functional divergence of homologous proteins. STIMs function as the Ca^2+^ sensor to detect changes of Ca^2+^ content in the intracellular stores. STIMs are single-span membrane proteins with unpaired N-terminal EF-hand Ca^2+^ binding domain critical for Ca^2+^ sensor function. In addition, STIMs contain an N-terminal sterile α motif (SAM) domain and a C-terminal (cytoplasmic) coiled-coil ERM domain (Liou et al. 2005; Roos et al. 2005; Williams et al. 2001; Zhang et al. 2005). Human STIMs and invertebrate STIM share several functionally important protein domains, but diverge significantly in the C-terminus.

The phylogeny and sequence analysis revealed early adaptation of the C-terminal divergent domain in Urochordata, before the expantion of STIMs in Vertebrata. STIMs were subsequently subjected to one round of gene duplication as early as in the Euteleostomi lineage in vertebrates, with a second round of fish-specific gene duplication. After duplication, STIM-1 and STIM-2 molecules appeared to have undergone purifying selection. Furthermore, sequence analysis of the EF-hand Ca^2+^ binding domain and the SAM domain, together with functional studies, identified critical regions/residues likely underlying functional changes and proved evidence for the hypothesis that STIM-1 and STIM-2 might have developed distinct functional properties after gene duplication (Cai, 2007).

### Transcriptome level

Alternative splicing is a common post-transcriptional process in eukaryotic organisms to produce multiple transcript isoforms from a single gene (Black, 2003). Alternative splicing and gene duplication are two sources of proteomic functional diversity (Fig. 1, A, B). According to the “independent model” alternative splicing and gene duplication are two independent mechanisms for increasing the proteomic complexity. Alternatively, the “function-sharing model” claims that some proteomic components can be performed either by alternatively spliced genes or duplicate genes [cited by (Su et al. 2006)].

Alternative splicing can generate multiple transcripts encoding proteins with subtle or opposing functional differences that may have profound biological consequences. Recent estimates, based on analyses of expressed sequence tags (ESTs), suggest that the transcripts from 35% of human genes are alternatively spliced (Hanke et al. 1999; Mironov et al. 1999). However, as pointed out by B. Graveley, “this number is likely to be an underestimate” (Graveley, 2001). First, the human EST collection does not represent all protein coding sequences and for most genes, ESTs cover only a portion of the transcript. Because much of the functionally significant alternative splicing occurs in the coding region of the transcript, many alternative splicing events might be overlooked by EST comparisons. Second, and perhaps more important, many alternative splicing events are very rare and occur only in a specific tissue at a specific time in development and/or under certain physiological conditions. These types of splicing events will probably not be represented in EST collections (Graveley, 2001). Some genes produce several spliced mRNAs while it turns out that some other genes, in fact, encode transcripts that are alternatively spliced to produce tens of thousands of different mRNAs (Schmucker et al. 2000).

The neurexin proteins represent an example which demonstrates the role of alternative splicing in obtaining different functions by one gene products. The neurexins are a family of neural proteins present in vertebrates that have important functions as receptors for neuropeptides (Missler and Sudhof, 1998) and as adhesion molecules that participate in synaptogenesis (Scheiffele et al. 2000). It has been shown that more than 1000 different neurexin mRNA isoforms could potentially be synthesized virtue of alternative promoter usage and alternative splicing (Ullrich et al. 1995). The proteins encoded by these alternative spliced mRNA have altered specificities for their ligands (Ichtchenko et al. 1995; Sugita et al. 1999). Beside that, the diversity of neuroxin proteins might specify neural connectivity (Missler et al. 1998). It has been shown that interaction of β-neurexins present in pre-synaptic cells with neuroligins on the surface of post-synaptic cells is sufficient to trigger synapse formation (Scheiffele et al. 2000). Importantly, this interaction only occurs if the β-neurexin is encoded by an mRNA lacking an alternative exon 20. Proteins synthesized from exon 20-containing transcripts do not interact with neuroligins (Ichtchenko et al. 1995). Thus, alternative splicing of the neurexin transcripts might have a direct role in controlling the formation and maintenance of synapses.

### Proteome level

Proteome is a set of proteins obtained by translation of mRNAs exported from nucleus to cytoplasm. Translation of mRNA into protein represents the final step in the gene-expression pathway, which mediates the formation of the proteome from genomic information (Gebauer and Hentze, 2004). As we have already underlined, at the genome level, the maximal volume of genetic information has to be encoded in minimal DNA space. At transcriptome level, the consealed diversity of future proteins encoded in DNA and mRNA is revealed by shuffling of different fragments of the primary transcript during the alternative splicing process. The alternative promoter usage in its turn discloses the diversity of genetic information contained in mRNA and destined for translation. At the proteome level, the maximum of structural and functional potentials contained in the genetic information embeded in mRNA can be elicited by translational mechanisms. Recent data obtained by sequencing of the human genome showed that the human genome composed of about 35 000 genes which may express about 100 000 proteins. Such protein outburst is possible only because numerous mechanisms exist by which functionally diverse polypeptides are produced from a single gene (Touriol et al. 2003).

The ability to produce different polypeptides by translation of one and the same mRNA molecule is installed in the translation mechanism by itself. Conceptually, the translation process can be divided into three stages—initiation, elongation and termination. During initiation the ribosome is reassembled on mRNA, with initiator tRNA bound in the ribosomal P site and base paired with the start codon of the mRNA. Elongation refers to the polymerization of the peptide, the main function of the ribosome, while termination includes the sequence of events following recognition of the stop codon up to the disassembly of the ribosome into subunits and subsequent dissociation of the factors, tRNA, and mRNA (Touriol et al. 2003). The mechanisms operating at each stage determine the appearance of different isoforms of one and the same gene product (Fig. 1, C–E).

#### A. Initiation stage

##### 1. Cap-dependent initiation

Translation initiation of eukaryotic mRNAs in general occurs by a scanning mechanism (Kozak, 2002). According to the linear scanning model, 40S ribosomal subunit binds to the cap structure (m7GpppN) at the 5′ end of mRNA and scanning downstream to the first in frame AUG initiation codon (Pestova and Hellen, 1999). The canonical scanning mechanisms (Fig. 1, C1) rules initiation of most mRNAs, but three non-classical cap-dependent initiation mechanisms have been also described: leaky scanning, ribosomal shunting and termination initiation (Lopez-Lastra et al. 2005).

###### a) Leaky scanning

It has been shown that the nucleotide context in the vicinity of the start codon is important for its interaction with the ribosome. If the context is not optimal, some ribosomes do not recognize the 5′end proximal AUG codon as starting, skip it, and begin translation at the next AUG by mechanisms known as “leaky scanning” (Kochetov, 2006; Kozak, 2002). This leaky scanning mechanism (Fig. 1, C2) produces protein isoforms with alternative amino initiation sites. Hence, the genes may produce several versions of the encoded proteins, and the shorter versions, initiated from a downstream in-frame start codons, lack the N-terminal amino acids of the full-length isoform version (N-terminal truncated version) (Touriol et al. 2003).

Besides additional AUG codons located downstream to the main start codon, many mRNAs contain upstream located AUG (uAUG) which also may be chosen by translational mechanisms for synthesis of new isofunctional protein variants. Kochetov et al. (Kochetov et al. 2005) had analyzed more than 12000 of human mRNAs bearing uAUGs and found that 3% of mRNAs cantained uAUGs in the same reading frame as the coding sequences without stop codons in between. It means that such mRNAs can code for additional protein variants extended at the N ends. A comparison of their predicted subcellular locations showed that 31% of such N-extended proteins had a different location, and in 19% of the cases they had secretion signals absent from the annotated variants (Kochetov et al. 2005).

As the sequences that determine cell compartment targeting are located usually at the N-terminal region of a protein, the selection of alternative translation initiation codons may be used by cell to control cellular localization of the isoforms (Cai et al. 2006; Gandre et al. 2003; Packham et al. 1997). Cai et al. (Cai et al. 2006) showed that 85.7% of alternative translation events generated biological diversity, attributed to different subcellular localizations and distinct domain contents in alternative isoforms. Several examples presented below may illustrate how the leaky scanning mechanism is used by a cell for increasing diversity of protein isoforms produced by a single gene.

The Arabidopsis DNA ligase 1 gene (AtLIG1) is indispensable for cell viability. AtLIG1 expresses one major and two minor mRNA transcripts differing only in the length of the 5′ untranslated leader sequences preceding a common open reading frame (ORF) (Sunderland et al. 2006). Control of AtLIG1 isoform production and intracellular targeting depends upon mechanisms controlling the choice of translation initiation site within the AtLIG1 ORF. Translation of AtLIG1 mRNA transcripts from the first in-frame start codon produces an AtLIG1 isoform that is targeted exclusively to the mitochondria. Translation initiation from the second in-frame start codon produces an AtLIG1 isoform targeted only to the nucleus. The length of the 5′-UTR and more significantly the nucleotide context around alternative start codons in the AtLIG1 transcripts affect translation initiation to ensure a balanced synthesis of both nuclear and mitochondrial AtLIG1 isoforms via a context-dependent leaky ribosome scanning mechanism (Sunderland et al. 2006). Instead of having distinct genes to code the different compartment-specific isoforms of DNA ligase proteins, eukaryotic species from yeast to humans appear utilize an evolutionarily conserved mechanism that relies upon choice of in-frame translation initiation start codons within the ORF of a ligase mRNA transcript to regulate the synthesis of the appropriate DNA ligase isoform destined for the nucleus or mitochondria. As the control of subcellular localization of proteins and their interaction with specific protein partners *in vivo* are crucial for cell physiology, the importance of the mechanisms controlling production of different forms of protein and their intracellular topology is difficult to overestimate (Sunderland et al. 2006).

Ion channels are composed of membrane-spanning proteins that allow ions to permeate at high rates. Voltage-gate K+ (Kv) channels are indispensable for the electrical excitability of nerve and muscle fibers (Finol-Urdaneta et al. 2006). The N-terminal region of the Kv channels plays important regulatory roles, including inactivation kinetics, subunits recognition and redox modulation of the currents flowing through those channels. Thus, the differenes among N-terminal regions of Kv channels can result in important functional differences between molecular forms of Kv channels. The Kv 1.7 channels from mouse heart muscle are encoded by *Kcna7* gene. Its mRNA transcript has two putative translation initiation start sites that generate two channel isoforms, mKv1.7L (489 aa) and mKv1.7S (457 aa), with different electrophysiological characteristics. The presence of both of these isoforms is very important for normal functioning myocard as absence of one of them leads to different types of heart pathology (Finol-Urdaneta et al. 2006).

Insulin-degrading enzyme (IDE) is expressed in both insulin-sensitive and insulin-insensitive tissues. Within the cell, IDE is targeted predominantly to cytoplasm, however, in some cells it presents also in several subcellular compartments. The mechanisms responsible for the targeting of IDE to different cell compartments are not well understood. Studing this issue, Leissring et al. (Leissring et al. 2004) showed that alternative translation beginning at an in-frame initiation codon located upstream of the canonical start site generates earlier nonrecognized isoform of insulin-degrading enzyme that in contrast to cytoplasm locating isoform is targeted to mitochondria. This example shows that N-terminal extension achieved by usage of alterantive initiation codon can produce new protein isoform possessing new function.

These and many other examples (Porras et al. 2006; Sass et al. 2001) demonstrate the power of leaky scanning mechanisms in generating multiple functionally different protein isoforms from single mRNA transcript.

###### b) Ribosome-shunting

The next translation mechanism that also makes impact in diversity of protein product coding for by a single gene is ribosome shunting. The scanning model postulates that when a scanning 40S ribosomal subunit encounters a hairpin loop in the 5′ UTR, it does not skip over the loop but unwinds it (Kozak, 1980; Lopez-Lastra et al. 2005). Nevertheless, there are some cases when a scanning 40S ribosomal subunit encounters the structures present in the 5′UTR and skips or shunts over a large segment, bypassing intervening segments including AUG codons and strong secondary structures that normally would block the scanning process (Fig. 1, C3). The selective translation initiation in adenovirus-infected cells under heat shock is an example of ribosome jumping mechanism (Yueh and Schneider, 1996).

###### c) Termination-initiation

The third non-classical cap-dependent initiation mechanism, as it has already mentioned above, is termination-initiation. In the reinitiation mechanism, a second ORF located in the same mRNAs can be translated without the 40S ribosome subunit becoming disengaged from the mRNA after reaching the first ORF stop codon (Fig. 1, C4). Strictly speaking this mechanism is more related to the elongation stage than to initiation one although the continuation of translation needs the reinitiation step at a downstream AUG triplet.

The exploration of the termination reinitiation mechanism for regulation of protein translation is observed in different cells and viruses. For example, stress-induced eIF2α phosphorylation increases translation of ATF4, an activator of the integrated stress response (ISR) program, by reinitiation mechanism (Lu et al. 2004). The ATF4 mRNA has two conserved upstream ORF (uORF), uORF1 and uORF2. Scanning ribosomes initiate translation at both uORFs. After translation of the uORF, the scanning ribosome efficiently reinitiate translation at downstream AUGs. In unstressed cells, low levels of eIF2α phosphorylation favor early capacitation of such reinitiating ribosomes directing them to the inhibitory uORF2, which precludes subsequent translation of ATF4 and repress ISR. In stressed cells high levels of eIF2α phosphorylation delays ribosome capacitation and favors reinitiation at ATF4 over the inhibitory uORF2 (Lu et al. 2004). Analogous mechanism operates in regulated translation of Gsn4 protein in yeast (Hinnebusch and Natarajan, 2002).

Translation of human hepatitis virus polymerase is another example demonstrating involvement of different translation mechanisms participating in expression of the polycistronic mRNA. The human hepatitis B virus (HBV) has a compact genome encoding four major overlapping coding regions: the core, polymerase, surface and X. The polymerase initiation codon is preceded by the partially overlapping core and four or more upstream initiation codons. Several mechanisms are used to enable the synthesis of the polymerase protein, including leaky scanning and ribosome reinitiation. Chen et al. (Chen et al. 2005a) showed that core upstream open reading frame (CO uORF), highly conserved in all HBV subtypes, played an inhibitory role in downstream expression from the core initiation site, but also stimulated reinitiation at the polymerase start when in an optimal context. Thus, the CO uORF is a determinant in balancing the synthesis of the core and polymerase proteins.

##### 2. Cap-independent IRES-determined initiation

The described examples demonstrate the possibilities of cap-dependent initiation mechanisms to generate multiple protein isoforms possessing new functions. However, the possibilities of translation process to increase and diversify the potentials embedded in mRNA are not restricted only to cap-dependent mechanisms as cap-independent translation mechanisms operate also in cells. Cap-independent translation (Fig. 1, C5) is mediated by internal ribosome entry sites, IRES (Holcik et al. 2000). These are usually comprised of structured regions in the 5′ untranslated region and were initially identified in viruses, but have since been reported in eukaryotic mRNAs and are believed to constitute a major form of regulation of protein synthesis in mammalian cells (Candeias et al. 2006; Holcik et al. 2000). As a rule, there are no significant structural similarities between individual IRES. At present, IRESs are defined solely by functional criteria and cannot yet be predicted by the presence of characteristic RNA sequences or structural motifs (Lopez-Lastra et al. 2005). Examination of the available RNA structure prediction software and RNA motif searching programs indicates that while these programs are useful tools to fine tune the empirically determined RNA secondary structure, the accuracy of *de novo* secondary structure prediction of large RNA molecules and subsequent identification of new IRES elements by computational approaches, is still not possible (Baird et al. 2006).

###### a) Mix (or alternative) initiation

It is important that, although capped, some cellular mRNAs—including those encoding translation initiation factors, transcription factors, oncogenes, growth factors, homeotic gene products and survival proteins-contain also IRES elements in their 5′UTR sequences that may allow them to be translated under conditions when cap-dependent synthesis of proteins is impaired (Lopez-Lastra et al. 2005). Moreover, in some cases mRNA molecule contains both cap-dependent start codon and IRES element and upon different cell conditions may direct synthesis of different isoforms of a single protein by using cap-dependent or IRES-mediated translation mechanisms (Fig. 1, C6). For instance, expression of two p53 isoforms, full length (FLp53) and N-terminal truncated (p53/47), is controlled by alternative cap-dependent and IRES-mediated mechanisms of initiation govern the translation of the p53 mRNA (Candeias et al. 2006). In contrast to FLp53, the p53/47 isoform does not harbour the most of N-terminal p53 transactivating domain (aa 1–40) nor the Mdm2-binding site (aa 17–23) demonstrating different functional parameters. The p53/47 may form complexes with FLp53 thus changing its stability and ability to induce activation of other p53-dependent genes. Changes in synthesis of FLp53 or p53/47 are regulated through distinct cell stress-induced pathways acting through separate regions of the p53 mRNA. The functional differences of two isoforms are manifested also in that that some cytotoxic agents require the presence of FLp53 to induce apoptosis, whereas for others p53/47 is sufficient. This indicates that by harbouring alternative translation initiation sites, the p53 mRNA gives rise to different levels of the p53 isoforms which help to orchestrate the cell biological outcome of p53 activation in response to different types of cell stress (Candeias et al. 2006).

Another example showing the role of alternative translation initiation in synthesis of protein isoforms possessing different functions is vascular endothelial growth factor (VEGF). The unusually long (1038 nucleotides) and structured 5′ untranslated region (UTR) of VEGF mRNA contains two independent IRES (IRES A and IRES B). In the human sequence, four potential CUG translation initiation codons are located in between these IRES and are in frame with the classical AUG start codon. The VEGF IRES A located within 300 nucleotides upstream from the AUG codon directs a cap-independent translation initiation, which has been shown to allow VEGF synthesis in hypoxic conditions (Huez et al. 1998; Sass et al. 2001; Stein et al. 1998). It has been shown that IRES B located in the first half of the 5′UTR between nucleotides 379 and 483 controls the translation initiation of new VEGF isoform (L-VEGF) which is N-terminal extended by 206 amino acids in comparison to the classical AUG-initiated form. Translational initiation of this isoform occurs at the first of CUG codons. Different functions of two VEGF isoforms can be evidenced by different targeting of AUG- and CUD-initiated isoforms: while the classical AUG-initiated VEGF isoform is a secreted protien, the CUG-initiated L-VEGF isoform demonstrates intracellular targeting. Besides that the L-VEGF undergoes post-translational partial cleavage into two fragments, namely a 206-a.a. N-terminal fragment that after cleavage remains in cell and a C-terminal fragment that is equeal to the AUG-initiated isoform by size and by ability to be secreted out of the cell (Huez et al. 2001).

#### B. Elongation stage

Thus, at the initiation stage of translation process the translation apparatus has numerous possibilities for material (molecular) realization of the information potentials consealed in mRNA. At the elongation stage other mechanisms may also make their impact in structural and functional diversity of synthesized protein isoforms. In contrast to the initiation and termination stages of translation, the machinery used during the course of translational elongation has been highly conserved and less variable across the three kingdom of life (Kapp and Lorsch, 2004). As noted by Namy et al. (Namy et al. 2004), the main mechanisms by which translation machinery may uncover the hidden genetic information at the elongation stage are the frame-shifting (Fig. 1, D2) and to a less degree—hopping (Fig. 1, D3).

##### 1. Frameshifting

Prediction the protein(s) encoded by a given DNA is difficult because of the lack of knowledge of the signals embedded in the genome that are involved in the translation of the genetic information. It has been shown that in many genes the standart rules of decoding postulated by the dogma of the universal genetic code are subverted by “recoding” signals found in DNA that promote alternative decoding events like programmed ribosomal frameshifting, hopping, or termination codon reassignment (Atkins et al. 2001; Gesteland and Atkins, 1996; Gesteland et al. 1992).

As noted by Namy et al. (Namy et al. 2004) “natural frameshift errors occur very reraly, but programmed ribosomal frameshifting signals increase the probability of tRNA slippage enormously, occasionally to such an extent that up to 50% of ribosomes change frame”. Analysis of the frameshifting mechanisms is beyond this review scope. They were critically studied and described in several excellent publications (Atkins et al. 2001; Brierley and Pennell, 2001; Plant et al. 2003; Stahl et al. 2001). In principle, frameshifting is triggered by two elements—a slippery and stimulator sequences. A slippery sequence is the mRNA region where tRNA movement or misalignment is favored while a stimulator is the mRNA sequence that ehhances framshifting by induction of a ribosomal pause (Namy et al. 2004).

Different kinds of frameshifting were described both in prokaryotes and eukaryotes. For example, programmed frameshifting was observed in translation of the prokaryotic DNA polymerase III, α-fucosidase and release factor 2 (RF2) as well as eukaryotic proteins such as antizyme-1 (AZ1), IL-10, ABP140 etc [cited by (Namy et al. 2004)]. The biological expediency and functions of such frameshifting are different in each case however it is always warranted. For instance, in the case of RF2 the recoding frameshift represents an auto-regulatory mechanism controling the abundance of RF2. At high RF2 levels, the competition between termination and frameshifting is shifted in favor of termination, leading to a decrease of the RF2 concentration in the cell. When level of RF2 begins to decrease, frameshifting begins to predominate, raising the level of RF2 (Adamski et al. 1993).

The antizyme-1 (AZ1) is another well studied example of a protein which full length expression is regulated by programmed frameshifting. AZ1 is a protein that negatively regulates polyamine synthesis by inhibiting the key synthetic enzyme ornithine decarboxylase and targeting it for degradation by the 26S proteasome (Heller et al. 1976; Murakami et al. 1992). Like RF2, the AZ1 full length protein expression depends on frameshifting which in its turn is linked to a feedback mechanism which regulates polyamine levels in mammalian cells. For AZ1, when cellular polyamine levels are low, the polypeptide corresponding to amino acid residues 1–68 is produced and translation is terminated at the following UGA stop codon (Matsufuji et al. 1995). When cellular polyamine levels are high, +1 frameshifting occurs after the 68th codon resulting in the full-length antizyme protein containing 227 amino acid residues (1–227 a.a.) (Ichiba et al. 1994). Frameshifting is employed in the expression of all known antizymes in different species and many other proteins. The concervation of this mechanism throughout evolution highlights a crucial role for frameshifting in the regulation of gene expression.

It should be pointed out that frameshifting determines multifunctionality of AZ1 only partially. Differential usage of alternative initiation sites also makes its impact in this phenomenone. AZ1 mRNA contains two initiation AUG codons separated by 32 codons. Both AUG triplets are utilized as translation start sites (Matsufuji et al. 1995; Rom and Kahana, 1994). It has been shown that N-terminal region of AZ1 contains nuclear export and mitochondrion targeting signals (Gandre et al. 2003; Gritli-Linde et al. 2001; Murai et al. 2003). Depending on either AUG codon is used for translation, the resulting AZ1 isoform is targeted to nucleus, mitochondria or cytoplasm (Murai et al. 2003). The multifunctionality of antizyme was evidenced not only by targeting of its isoforms to different cell compartments and serving as ornithine decarboxylase inhibitor but also by recent finding that AZ1 functions as positive transcriptional regulator of the atoDAEB operon genes in E.coli (Filippou et al. 2007).

We have described here only two proteins which synthesis are directed by frameshifting. Many other examples in which this mechanism is used for development of protein diversity may be found in very informative review written by Namy et al. (Namy et al. 2004).

##### 2. Hopping (translational bypassing)

An unusual frameshift event that also diversifies the protein structures and functions is “hopping” where the transition from frame 0 to another frame occurs by translational bypassing of an extended region of the mRNA sequence rather than by slippage past a single nucleotide, as has been described for most examples of frameshifting. When genetic code has been discovered, its translation seemed to be rigid. However, later on the reading of the genetic text (code) has been found to be quite flexible, and several alternatives in its expression have been described (Groisman and Engelberg-Kulka, 1995).

Translational bypassing joins the information found within two disperate open reading frames into a single polypeptide chain. The underlying mechanism centers on the decoding properties of peptidyl-transfer RNA (tRNA) and involves three stages: *take-off,* in which the peptidyl-tRNA/mRNA complex in the P-site of the ribosome dissociates; *scanning,* at which the peptidyl-tRNA probes the mRNA sliding through the decoding center; and *landing,* in which the peptidyl-tRNA repairs with a codon with which it can form a stable interaction (Fig. 1, D3) (Herr et al. 2000a). The best characterized examples of this phenomenon are T4 gene 60 and plaA gene (the *Prevotella loescheii* adhesion gene). In T4 gene 60, a complex signals stimulates bypassing of 50 nucleotides between the two open reading frames (Herr et al. 2000b). In plaA, the translation of its mRNA requires bypassing of 29-nt of coding gap (Manch-Citron et al. 1999).

Herr et al. (Herr et al. 2000a) noted that although few examples of genes are known that rely on translational bypassing to couple ORFs, ribosomes appear to have innate capacity for bypassing. This suggests that the strategy of translational bypassing may be more common than presently appreciated.

#### C. Termination stage

Termination of protein synthesis involves the decoding of a stop signal through an interaction between ribosome, mRNA and release factors (RF) that facilitates the hydrolytic release of the nascent polypeptide chain from the peptidyl-transferase center of the ribosome (Cridge et al. 2006). Stop codons are used to signal the ribosome to terminate the decoding of an mRNA template. Open reading frames within an mRNA are terminated by an in-frame any one of three stop or nonsense codons (UAA, UAG or UGA) which are recognized during translation by the binding of RFs to the ribosomal A site (von der Haar and Tuite, 2007; Williams et al. 2004).

##### 1. Stop codon readthrough

Translation termination is normaly a highly efficient process. It has been shown that stop codons are normally suppressed at a frequency of only 0.001%–0.1% (Loftfield and Vanderjagt, 1972; Stansfield et al. 1998). Two competing events, termination and stop codon readthrough (or nonsense suppression), can occur when stop codon reaches the A-site of a translating ribosome. Translation termination results in hydrolysis of the final peptidyl-tRNA bond and release of the completed nescent polypeptide. Alternatively, readthrough, in which the stop codon is erroneously decoded by a suppressor or near-cognate t-RNA, results in translation past the stop codon and production of a protein with a C-terminal extention (Fig. 1, E). The relative frequency of termination versus readthrough is determined by parameters such as the upstream and downstream stop codon nucleotide contexts, the activities of termination polypeptide-chain release factors and the abundance of suppressor near-cognate t-RNAs. All these parameters modulate the balance of termination to readthrough reactions in a cell-type-specific manner (Bonetti et al. 1995; Fearon et al. 1994; Janzen and Geballe, 2004; Keeling et al. 2004; Namy et al. 2002). Willams et al. (Williams et al. 2004) underline that “the potential for even short C-terminal extention (peptide tags) to alter the cellular localization or activity of a protein is great. Addition of even a single amino acid could complete a partial targeting signal already present at the C-terminus of a protein. All protein extensions altering targeting could potentially act as genetically dominant, gain of function events. Thus, despite the generally low efficiency of stop codon readthrough events, such events may well have phenotypic consequence for the cell”.

There are an increasing number of examples of stop codon readthrough, both in viral and cellular systems, and in many cases this readthrough has physiological consequences for the cell. It is become obviously that different cells and organisms not only accommodate stop codon as a mainstream termination signal, but also used it to provide these cells and organisms flexibility to respond physiologically to various changeable environments (Namy et al. 2002; Steneberg and Samakovlis, 2001; Tate et al. 1999; Yoshinaka et al. 1985). The alternative non-canonical usage of stop codons allows production of several polypeptides from one open reading frame thus increasing structural and functional diversity of one gene products (Doronina and Brown, 2006). The classical illustration to this statement is selenoproteins.

More that 15 animal selenoproteins have been described. Many selenoproteins, such as glutathi-one peroxidase (Sukenaga et al. 1987), type I iodothyronine 5′-deiodinase (5′DI) (Berry et al. 1991b) and thioredoxin reductase (Gasdaska et al. 1995) are enzymes which catalyze oxido-reduction reaction. All selenoproteins contain selenocysteine (Sec). Selenocysteine incorporation is specified by a UGA codon in the open reading frame of the mRNA that is accompanied by a “selenocysteine insertion sequence” element (SECIS) in the 3′ UTR (Berry et al. 1991a; Goto et al. 2001). However, in definite conditions this UGA codon may also serve as a stop codon. The competition between UGA as a stop signal and as the code for the Sec has been observed during expression of the deiodinase gene. Both the termination product (14 kD) and the complete iodinase protein (28 kD) could be detected within cells (McCaughan et al. 1995). The relative amounts of these products could vary with cell type and expression system, reflecting a competition between the two UGA decoding mechanisms (Steneberg and Samakovlis, 2001).

The selenoprotein P represents even more strong illustration of competition between UGA as a stop codon and UGA as a triplet that specifies Sec-incorporations. Selenoprotein P is an extracellular glycoprotein that has been suggested to serve in oxidant defense (Burk et al. 1995). Its mRNA contains 10 UGA codons in an open reading frame terminated by a UAA, implying 10 selenocysteines in the primary structure of the protein (Hill et al. 1991). Full-length selenoprotein P and three smaller isoforms that have identical N termini but different affinity to heparin-Sepharose have been detected in rat plasma (Chittum et al. 1996). By mass spectrometric peptide analysis it was evidenced that the full-length isoform contains predicted amino acid residues, including its C terminus and all 10 selenocysteines. The shortened isoforms terminated where the second, third and seventh selenocysteines residues were predicted to be (Ma et al. 2002). This suggests that all isoforms arise from the same mRNA and that the UGAs that specify the second, third and sevenths selenocysteines in full-length selenoprotein P can alternatively serve to terminate translation, producing the shorter isoforms. As Ma et al. (Ma et al. 2002) pointed out, “it remains a theoretical possibility that the shorter isoforms are produced from the full-length protein by proteolysis”. However, the protease that could cleave the protein at specific selenocysteine residue is unknown, and this excludes the possibility of proteolytical cleavage of the full-length isoform at the specific Sec-sites.

The *Drosophila kelch* gene is another example showing that translational control of gene expression may be realized through stop codon suppression and readthrough. It has been demonstrated by Robinson and Cooley (Robinson and Cooley, 1997) that the *kelch* gene produces a single transcript with a UGA stop codon separating two open reading frames (ORF1 and ORF2). From the transcript, 76 kDa ORF1 and 160 kDa full-length (ORF1+ ORF2) proteins are expressed. The expression of these two proteins is regulated in tissue-specific manner causing the ratio of two isoforms to vary in different tisuues (Robinson and Cooley, 1997).

The important role of “leaky” stop codon and readthrough mechanism for cell physiology can be illustrated also by the yeast *PDE2* gene which encodes the high affinity cAMP phophodiesterase. By synthesis the cAMP phosphodiesterase this gene may regulate the intracellular concentration of cAMP and thus protect the cell from extracellular cAMP (Wilson et al. 1993; Wilson and Tatchell, 1988). When the translation of the *PDE2* mRNA is terminated at leaky stop codon, the pde2 protein (active phosphodiesterase) is synthesized leading to low level of intracellular cAMP and increased stress sensitivity. On the contrary, when stop codon readthrough mechanism is switched on, the C-terminal extension of 21 amino acids occurs leading to pde2 protein destabilization which in its turn results in high level of cAMP in the cell and resistance to stress environment (Namy et al. 2002; von der Haar and Tuite, 2007). The *PDE2* is thus an example of an individual gene product in *Saccharomyces cerevisiae* where alterations in stop-codon readthrough are directly linked to phenotypic variations that have crucial consequence for cell physiology.

When physiological roles of canonical termination signals, leaky stop-codons and readthrough mechanisms are discussed, the principle difference between normal function of termination stop codon and abnormal role of premature stop codon(s) which may appear in the mRNA as result of mutations or alternative splicing to cryptic splice sites should be underlined (Frischmeyer and Dietz, 1999; Lewis et al. 2003). The nucleotide context surrounding the classical stop codon is genetically determined and promotes translational termination of the synthesized natural protein. The upstream and downstream contexts of leaky stop signal are also genetically determined for promotion of the readthrough activity which results in appearance of new protein isoform that is necessary for fulfillment of specific physiological function. Thus, the expression of terminated or extended proteins in these cases represents realization of specific steps in the normal cell program. The random appearance of the premature termination codon (PTC), or nonsense codon, in ORF of mRNA is not correlated with the inherited surrounding nucleotide context and therefore cannot be regulated by physiological translation mechanisms (Lewis et al. 2003).

Nonsense codon-bearing transcripts typically encode C-terminally truncated proteins which could possess dominant-negative or deleterious gain-of-function effects. Usually, PTCs trigger the rapid degradation of mRNA by nonsense-mediated mRNA decay (NMD) mechanism that detects and degrades transcripts harboring nonsense codons (Frischmeyer and Dietz, 1999; Wilkinson, 2005). This downregulatory response is an important quality-control mechanism. Its function is safeguarding cells from abnormal mRNA function (Behm-Ansmant et al. 2007; Isken and Maquat, 2007). Surprisingly, recent studies strongly evidenced that components of NMD machinery (proteins UPF1, UPF2, UPF3, RNP21, Y14 and MAGOH) also promote translation of normal mRNAs in mammalian cells (Nott et al. 2004; Wiegand et al. 2003; Wilkinson, 2005). It has been shown that the function of NMD proteins depends on their location. If located within an ORF, NMD factors stimulate translation and control high-fidelity translation termination. If located downstream of an ORF, they elecit RNA decay (Lykke-Andersen et al. 2001; Maderazo et al. 2000; Wang et al. 2001; Wiegand et al. 2003; Wilkinson, 2005). Thus, NMD proteins also demonstrate multifunctionality participating in two oposite processes. On the one hand, by takeing part in NMD, they degrade mRNA and decrease the level of protein synthesis. On the other hand, by binding to mRNA within ORF, they stimulate protein syntesis and even correct naturally occurring mistakes (Wilkinson, 2005).

The described examples not only document the existence of several evolutionary conserved mechanisms of translation destined for production of multiple and functionally different isoforms of one gene product but also show that proteins involved in these mechanisms are multifunctional molecules that simultaneously may participate in opposite processes leading to decreasment and to increasment of protein synthesis. These mechanisms make a substantial impact in transformation of compact genetic information in diverse protein moiety.

### Post-translational modifications

Although the amino acid structure of a protein is defined by the gene and mRNA encoding the protein, its final functional structure is achieved by numerous post-transclational modifications. The majority of all proteins undergo co- and/or post-translational modifications (Rehfeld and Goetze, 2003). Knowledge of these modifications is extremely important, since they may alter physical and chemical properties, conformation distribution, folding, stability, activity, and, consequently, function of the proteins. The protein precursor may undergo a wide variety of proteolytic cleavages, N- and C-terminal trimmings and amino acid derivatization in cells that express the protein. Occasionally, the same precursor is differently processed in different cell types (Rehfeld and Goetze, 2003). According to Rehfeld and Goetze (Rehfeld and Goetze, 2003) all post-translational modifications may be divided in three groups [Table 1 cit. from (Rehfeld and Goetze, 2003)].

Although this table contains many types of post-translational modification, it can not be considered as a complete list of possible modification. For example, it does not contain such modification as palmitoylation, ubiquitylation, methylation, protein folding and protein splicing which appear to be a powerful mechanism of protein diversity (Draper et al. 2007; Hanada et al. 2004; Mostaqul Huq et al. 2008; Nagradova, 2004; Perler, 2005; Schartner et al. 2007). The limit of the review volume does not allow discussion of all the possible post-translational modifications depicted in Fig. 1, F. Therefore, we will concentrate just on some of them—phosphorylation, glycosylation, palmitoylation, protein folding and protein splicing.

#### 1. Protein phosphorylation

Protein phosphorylation is one of the most widespread type of post-translational modification of protein that allows the cell to control various cellular processes, including signal transduction, transcriptional and translational mechanisms, metabolism, growth, division, differentiation, motility, organelle trafficking, membrane transport, immunity, learning and memory (Manning et al. 2002a; Manning et al. 2002b). In eukaryotic cells, most phosphorylation occurs on Ser, Thr and Tyr residues (Ubersax and Ferrell, 2007). The phosphorylation state of phosphoproteins is controlled by the activity of protein kinases and phosphatases (Urner and Sakkas, 2003). According to calculation performed by Ubersax and Ferrell (Ubersax and Ferrell, 2007), a typical protein kinase must recognize between one and a few hundred *bona fide* phosphorylation sites in a background of about 700,000 potentially phosphorylatable residues in typical eukaryotic cell. Multiple mechanisms have evolved that contribute to this exquisite specificity, including the structure of the catalytic site, local and distant interactions between the kinase and substrate, the formation of complexes with scaffolding and adaptor proteins that spatially regulate the kinase etc (Ubersax and Ferrell, 2007).

Although different kinases demonstrate their individual specificities and peculiarities in phosphorylation processes, the main results of this post-translational modification are structural and functional changes of the substrate protein. Several examples can illustrate this statement.

Many nuclear receptors have been found to be modified by phosphorylation (Rochette-Egly, 2003). Testicular receptor 4 (TR4) is an orphan member of the nuclear receptor superfamily. In the absence of specific ligands the activity of TR4 can be modulated by mitogen-activated protein kinase (MAPK)-mediated phosphorylation of its AF-1 domain (activation function 1). MAPK-mediated phosphorylation of the AF-1 domain renders TR4 a repressor while dephosphorylation of the AF-1 domain makes TR4 an activator (Huq et al. 2006). Reversible protein phosphorylation, like bidirectional regulation of TR4, is known to control a wide range of biological activities (Chen et al. 2005b; Collins et al. 2004; Kim et al. 2003; Koritschoner et al. 2001; Shyr et al. 2002; Tanabe et al. 2002).

Another example also may show the power of post-translational phosphorylation in determing the protein function. Glucose-mediated phosphorylation converts the transcription factor Rgt1 from a repressor to an activator (Mosley et al. 2003). In the yeast *Saccharomyces cerevisiae*, glucose induces the expression of the hexose transported/*HXT*/genes by modulation the activity of transcription factor Rgt1 that functions as a repressor when glucose is absent. However, in the presence of high concentration of glucose, Rgt1 is converted from a repressor to an activator by glucose-mediated phosphorylation. Rgt1 activator is required for maximal induction of *HXT1* gene expression (Mosley et al. 2003).

Several transcription factors, as well as other proteins, have been shown to change their subcellular localization in response to external stimuli (De Vit et al. 1997; Komeili and O’Shea, 1999). Yeast protein Mig1 is phosphorylated by the Snf1 kinase in the absence of glucose and is trapped in the cytoplasm. High concentrations of glucose cause dephosphorylation of Mig1 and translocation into the nucleus (Komeili and O’Shea, 1999; Treitel et al. 1998). The subcellular localization of the other yeast protein, Pho4, is regulated in response to another agent, phosphate. At high concentrations of phosphate, Pho4 is phosphorylated and exported into cytoplasm, whereas starvation for phosphate causes dephosphorylation of Pho4 and translocation into the nucleus (Komeili and O’Shea, 1999).

These examples clearly demonstrate that definite functional potentials encoded in the primary protein structure by specific topology of Ser, Thr and Tyr residues may be uncovered and realized by specific phosphorylation events.

#### 2. Glycosylation

Glycosylation is another widely observed post-translational modification of proteins. Proteins can be glycosylated on certain amino acid side-chains, and these modifications are designated as N- and O-glycosylation (Medzihradszky, 2002). N-glycosylated species are modified at Asn residues. O-glycosylation occurs at Ser or Thr-residues. It should be noted that characterization of both types of glycosylation is complicated by the fact that in some cases the same amino acids within a population of protein molecules may be derivatized with an array of different carbohydrate structures while in other cases they may remain unmodified. This site-specific heterogeneity may vary by species, tissue and may be affected by physiological changes (Medzihradszky, 2002). Thus, the same protein may be glycosylated in one type of cells and unmodified in another type of cell and the difference in glycosylation status will be reflected by different functions of a given protein in different cells (Bloom et al. 1996; Hironaka et al. 1993). The analogous situation can be observed even in the same cells, however, at different physiological conditions (Nemansky et al. 1998).

The purpose of glycosylation, as any other post-translational modification, is to uncover functional activities hidden in the protein structure. By adding sugars to polypeptide molecule, glycosylation changes it structure in such a way that new function(s) may occur. For instance, Miranda et al. (Miranda et al. 2007) showed that neutralization properties of the HIV-specific antibody F240 were dramatically altered by glycosylation. This Ab, when produced in a hybridoma, is nonneutralizing. However, F240 IgG1 Ab expressed in CHO cells acquired a strong neutralization activity without a change in immunoreactivity. Sequencing of the F240 mRNA produced in the parental hybridoma and CHO cells revealed identical sequences, suggesting that acquired neutralization resulted from cell-specific post-translational modifications. Among different types of post-translational modification which the Abs may undergo during their intracellular maturation process, N-linked glycosylation is one of the best characterized. It is widely acknowledged that an Ab expressed in CHO cells is differentially glycosylated as compared with an Ab produced in other mammalian cells (Routier et al. 1997; Sheeley et al. 1997). Miranda et al. (Miranda et al. 2007) showed that the Ab produced by CHO cells is glycosylated to a greated extent than parental Ab produced by the hybridoma. It was found that the F240 Ab produced in CHO cell had significantly more terminal glycans and core fucose when compared with the F240 Ab from hybridoma. Moreover, treatment with peptide N-glycosidase F abrogated F240 neutralization. This study showed that structural changes induced by additional N-glycosylation in the F240 Ab determined functional changes in its neutralizing activity.

Two other examples show the effect of O-glycosylation on the conformation and biological activities of two other proteins, calcitonin and prion protein. Using different O-glycosylated calcitonin derivatives, each with a single GalNAc residue attached to either Ser or Thr, Tagashira et al. (Tagashira et al. 2001) have demonstrated that calcitonin conformation and its hypocalcemic activity were strongly dependent on O-glycosilation. Moreover, these authors evidenced that effect of glycosylation was clearly site-specific: glycosylation at Thr-6 affected both the structure and activity, at Ser-5 only activity and at Ser2 none, indicating that the site dependence was very strict. The drastic difference in the hypocalcemic activity observed in this study could be explained by the steric inhibition of the interaction with the receptor (Tagashira et al. 2001).

It has been known for a long time that the structural transition from PrP-c, the normal product of the prion gene, to PrP-sc, the pathological form of the protein, leads to the prion formation and to disease, however, the mechanism of this transition was obscured. Based on prion peptide studies, Chen et al. (Chen et al. 2002) provided evidence for linking of O-glycosylation to the structural transition of PrP-c to PrP-sc. They found that O-linked α-GalNAc at Ser-135 suppressed the development of the amyloid fibril formation of the prion peptide at physiological salt concentration, whereas the peptide with the same sugar but at different location, at Ser-132, showed the opposite effect. Moreover, this effect was sugar specific: replacing α-GalNAc with β-GalNac did not yield the same effect (Chen et al. 2002).

As we have alredy noted, many types of post-translational protein modifications have been described (Rehfeld and Goetze, 2003). They all are directed to modify structure of the parental polypeptide chain thus increasing number of protein’s functions. It is *apriori* obviously, that for achieveing maximal functional variability and adaptivity to environment by post-translational modification, the compact polypeptide structure must contain sites that might be sensitive to different type of modification. Moreover, the same site could be sensitive to different modifying reactions, so the different modifying agents could compete for these sites (competitive modification). The correctness of such suggestion was confirmed by the discovery that phosphorylation and glycosylation may compete for and modify the same amino acid residues in targeted proteins (Kelly et al. 1993).

The O-linked β-N-acetylglucosamine (O-GlcNAc) is a monosaccharide modification abundant on serine and threonine residues of a multitude of nucleo-cytoplasmic proteins in virtually all higher eukaryotes, including plants and fungi (Comer and Hart, 2000; Hart, 1997; Hart et al. 2007; Torres and Hart, 1984; Wells and Hart, 2003). The attachment and removal of O-GlcNAc by O-GlcNAc-transferase and O-GlcNAcase, respectively, is rapid reactions analogous to dynamics of O-phosphate adding or removing controlled by kinases and phosphatases (Dong and Hart, 1994; Haltiwanger et al. 1992; Haltiwanger et al. 1990; Krebs, 1993; Kreppel et al. 1997; Kreppel and Hart, 1999; Lubas et al. 1997). It has been shown that O-GlcNAc sites resemble phosphorylation sites, and in many cases the two modifications are mutually exclusive (Kelly et al. 1993). Reversible phosphorylation and alternative glycosylation induce changes in secondary and tertiary structures and control the functional behaviour and modular interactions of proteins (Ahmad et al. 2007; Cheng and Hart, 2001; Pawson and Gish, 1992). For example, in case of estrogen receptor β (Cheng et al. 2000), SV-40 large T antigen (Medina et al. 1998) and the c-Myc oncogen (Chou et al. 1995a; Chou et al. 1995b), O-GlcNAc and O-phosphate compete for the same hydroxyl moiety. Like phosphorylation, O-GlcNAcylation is responsive to the cell cycle, extracellular signals, glucose metabolism, cell growth and regulation of transcription and translation (Chou and Omary, 1994; Datta et al. 1989; Du et al. 2000; Han et al. 2000; Kearse and Hart, 1991; Roos et al. 1997; Roquemore et al. 1996).

The examples described above show once again that primary protein has many possibilities to be modified. What type of modification it will undergo depends on many factors such as cell type, cell cycle, extracellular and intracellular signals, pH gradient, local concentrations of specific modifying molecules etc. As a result, the protein will possess a specific function in an appropriate cell, at definite cell compartment and at the precise time.

#### 3. Protein palmitoylation

The next type of protein modification that we would like to discuss is S-palmitoylation. A major mechanism by which cells regulate the subcellular localization of proteins is post-translational lipidation (Draper et al. 2007). S-palmitoylation is one of lipid modifications that is reversible thioester linkage of palmitate, a 16 carbon saturated fatty acid, to cystein residues (S-palmitoylation) catalyzed by membrane-bound palmitoyl-transferases (PATs). Palmitoylation increases the hydrofobicity of the modefied molecules (Greaves and Chamberlain, 2007).

It is *apriori* obviously, that simultaneous display of the principles of structural compactness with maximal functional variability demands that sites on the polypeptide molecule that may undergo different types of modification should be overlapped or even be the same, and different modifying agents should compete for these sites of proteins by altering their conformation. The changes in structure of proteins promotes their localization to specific cellular compartments, contributes to their membrane association and control protein-protein interactions. They also regulate subcellular trafficking of proteins between membrane organelles and within microdomains of the same membrane compartments and modulate their functions (Bijlmakers and Marsh, 2003; Casey and Seabra, 1996; Dunphy and Linder, 1998; el-Husseini Ael and Bredt, 2002; Milligan et al. 1995; Nadolski and Linder, 2007; Resh, 1999; Silvius, 2002).

For many palmitoylated proteins, the half life of the palmitate moieties is significantly shorter than that of the substrate proteins, indicating that the complex goes through cycles of depalmitoylation and repalmitoylation. The reversibility of palmitoylation suggests that it is a regulated modification much like protein phosphorylation. Depalmitoylation and repalmitoylation could provide a mechanism to regulate such processes as binding cytosolic proteins to membranes, segregating proteins to microdomains and mediating protein-protein interaction. For proteins involved in signal transduction, these cycles could be induced by activation and by controlling access to specific substrates (Bijlmakers and Marsh, 2003; Casey and Seabra, 1996; Dunphy and Linder, 1998; el-Husseini Ael and Bredt, 2002; Milligan et al. 1995; Nadolski and Linder, 2007; Resh, 1999; Silvius, 2002). Palmitate cycling on the scaffolding protein PSD-95 may illustrate this mechanism. Palmitoylation allows PSD-95 to cluster in the postsynaptic membrane. Blocking of PSD-95 palmitoylation leads to a loss of AMPA receptors from these domains by the rapid endocytosis of AMPA receptors which requires depalmitoylation of PSD-95 (el-Husseini Ael and Bredt, 2002; Nadolski and Linder, 2007).

Several other examples can additionally illustrate the roles of palmitoylation in protein functioning. It has been shown that palmitoylation controles the regulator of G-protein signaling (RGS). Palmitoylation of the Cys residue in the RGS box is essential for RGS16 GAP activity and their ability to regulate G-coupled signaling in mammalian cells. Enzymatic palmitoylation of RGS16 results in internal palmitoylation on residue Cys-98. Mutation of this residue to alanine reduces GAP activity of the 5-HT-1A/Gα fusion protein (Osterhout et al. 2003).

S-palmitoylation, as shown by study of Marino et al. (Marino et al. 2006), can modulate estrogen receptor α (ER α) localization and functions. In cells expressing wild-type of ERα, a major population of estrogen receptor was membrane-associated whereas in cells expressing the ERα Cys447->Ala mutant no membrane-bound receptor has been detected. Moreover, ERα Cys447->Ala mutation does not allow E2-induced proliferative signaling via ERK/MAPK and P13K/AKT pathways in human cancer cells (Acconcia et al. 2005). As a whole, S-palmitoylation allows the extra-nuclear localization of ERα and promotion downstream of signaling for E2-mediated proliferation (Marino et al. 2006).

S-palmitoylation, like phosphorylation and glycosylation, is important post-translational modification that provides proteins with many important functions and controls cell processes dependent on these functions. Thus, as other post-translational modifications, palmitoylation fulfills the main principle of protein structural/functional relationship: gaining maximal functional activities by introducing minimal but specific changings in the protein structure.

#### 4. Protein folding

The protein architecture opens many ways for proteins to obtain multiple functions. Evolution gives many examples confirming this statement. In some cases, evolution can merge proteins by chaning tertiary to quaternary structure. For instance, five separate enzymes in the bacterium *E.coli* that catalyse successive steps in the pathway of biosynthesis of aromatic amino acids, correspond to five regions of a single protein in the fungus *Aspergillius nidulans* (Lesk, 2001).

To become functionally active, a newly synthesized polypeptide chain must fold a unique three-dimentional structure. Although experiments *in vitro* have firmly established that the information on the three-dimentional structure of a protein is genetically determined and contained in its amino acid sequence (Anfinsen, 1973; Dobson and Karplus, 1999; Nagradova, 2004), protein folding in the cell cannot be regarded as occurring spontaneously (Nagradova, 2004). It has become clear that many proteins require assistance to fold in the cell and that this is provided by helper proteins (molecular chaperones) (Bukau and Horwich, 1998; Coyle et al. 1997; Fewell et al. 2001; Gutsche et al. 1999; Hartl and Hayer-Hartl, 2002).

The action of chaperones is based on two different mechanisms. The first mechanism consists of maintaining the polypeptide chain in a state capable of productive folding, which occurs spontaneously after release of unfolded chains into solution. Thus, interaction with the chaperone has no effect on the folding *per se* (Nagradova, 2004).

The second mechanism is used by chaperons, whose large cylindrical complexes create physically isolated compartments destined for the folding of polypeptides, partially folded or misfolded proteins, which become encapsulated inside the central cavity. Since only one molecule of the substrate protein can fit inside the cavity, its folding takes place under conditions, which completely exclude aggregation (Nagradova, 2004). However, the role of chaperons clearly is not limited to their ability to create isolated compartments where protein can fold spontaneously under condition simulating infinite dilution (the “Anfinsen cage” model) (Ellis and Hartl, 1996). A large body of information accumulated to date supports the notion that chaperons can play an active role in protein folding, accelerating this process and considerably increasing its efficiency (Nagradova, 2004). A special group of helper proteins so called foldases (Balbach, 2000; Wu and Matthews, 2002) and protein disulfide isomerases (Creighton, 2002; Frand et al. 2000) assist protein folding by catalyzing the rate-limiting isomerization reactions.

#### 5. Protein splicing

A simple view of protein expression is continually challenged by new examples of post-translational processing. These examples include non-ribosomal addition of moieties and proteolytic cleavage of polyprotein as well as excision and rearrangements such as intein-mediated protein splicing, splicing by reverse proteolysis, protein auto-processing and proteasome-mediated peptide ligation (David et al. 2004; Hanada et al. 2004; Noren et al. 2000; Paulus, 2001; Perler, 2005; Perler et al. 1994; Vigneron et al. 2004).

##### a) Intein-mediated protein splicing

Inteins are internal segments of precursor proteins that catalyze their *ipso* excision by an intramolecular process called protein splicing, with the concurrent ligation of the two flanking external regions (N- and C-exteins) through a native peptide bond (David et al. 2004; Perler, 2005). Inteins are found in proteins of unicellular organisms belonging to all three domains of life Archaea, Bacteria and Eukarya as well as in viral and phage proteins but have not jet been detected in higher organisms such as Vertebrata (Hanada and Yang, 2005). More than 100 inteins are known. Intein host proteins are very diverse, including DNA and RNA polymerases, ribonucleotide reductases, ATPases, proteases, metabolic enzymes, transcription and translation factors (Pietrokovski, 2001). The following properties differentiate intein-mediated protein splicing from other types of protein rearrangements: (a) intein signature sequence, (b) a self-catalytic reaction in the absence of exogenous proteins, cofactors or energy sources, and (c) the formation of a native peptide bond between the ligated exteins (Perler, 2005).

Inteins can be divided into four classes: the maxi inteins (with integrated endonuclease domain), mini inteins (lacking the endonuclease domain), trans-splicing inteins (where the splicing junctions are not covalently linked) and Ala inteins (Ala as the N-terminal amino acid) (Evans and Xu, 2002). The intein specific sequences have some characteristics in common. They appear in conserved regions of the host protein. All intein sequences harbor different blocks termed A and B at the N-terminal splicing domain and F and G at the C-terminal splicing domain.

Inteins typically have N-terminal Cys, Ser or Ala residues and C-terminal His or Asn and inserted into host proteins next to Cys, Ser or Tre (Hanada et al. 2004). Although amino acids indicated above are typical for inteins, many inteins have other amino acids at specific blocks. It is likely that each intein has specific combination of nucleophiles and assisting groups honed during evolution for optimal function. Structural studies have not identified more consensus facilitating residues because each intein has different residues with similar chemical functionalities at or near its active site (Perler, 2005). The smallest known intein consists of 134 amino acids (Evans et al. 1999), and most are much larger.

The role of inteins in the cell is as yet unclear, however, one can assume that because inteins are integrated in highly conserved protein regions, the function(s) of host protein before and after excision of intein might be different. If intein has to excise itself for activation the host protein function, it has to be sensitive to specific signals. There are some indications that this is the case (Chong et al. 1998; Southworth et al. 1999). It has been established that intein regulates activity of *Synechocystis* sp.PCC6803 DnaE functioning as internal regulator (Ghosh et al. 2001). Free inteins (after splicing) may perform regulatory as well as enzymatic function by itself (Klabunde et al. 1998; Starokadom-skyy, 2007). Thus, although it has been evidenced only in limited number of proteins, inteins may determine different functions of host protein before and after splicing and to obtain new function(s) after self-excision.

##### b) Post-translational splicing by reverse proteolysis

Another example of post-translational modification, protein splicing by reverse proteolysis, is a protein splicing discovered in plants. It was observed with the lectin concanavalin A (Con A) (Carrington et al. 1985). The following steps of this kind of protein splicing were described. The initial precursor of Con A (glyco-pro-Con A) is first activated by deglycosylation to pro-Con A. Pro-Con A is then cleaved to produce two distinct proteins that are transposed and relegated to become mature Con A. Although the exact mechanism of this splicing in plants is still not clear, *in vitro* study showed that asparaginyl endopeptidase can digest Con A and then re-ligate the digested fragments by its reverse proteolytic activity (Min and Jones, 1994).

##### c) Proteasome-mediated protein splicing

Recently it has been shown that proteasomes are cell compartments where not only protein degradation but also post-translational protein modifications occur (Hanada and Yang, 2005; Hanada et al. 2004; Vigneron et al. 2004). Hanada et al. (Hanada et al. 2004) discovered that antigen maturation and presentation may be performed in proteasome by protein splicing involving peptide excision and re-ligation. They showed that in contrast to intein splicing that usually excised polypeptide of not less than 134 amino acids in length (the smallest known intein is consist of 134 amino acids) the excised polypeptides in their system were as short as 18–40 amino acids. Using the proteasome inhibitor, clasto-lactacystin β-lactone, the authors evidenced that FGF-5 protein splicing observed in their system was proteasome-mediated (Hanada and Yang, 2005). Moreover, these authors showed for the first time that protein splicing may occur in human cells.

Another group also described an antigen production from a protein by splicing of noncontiguous peptides in the reverse order (Vigneron et al. 2004). This splicing was conducted by 20S proteasome *in vitro* and *in vivo*. In the described systems the length of excised intervening region was only four amino acids (Noren et al. 2000; Vigneron et al. 2004). Thus, the proteasome-mediated splicing overcomes the limitation associated with the length of intervening sequences which is crucial for intein-mediated protein modification.

What are the implications of these findings? As Hanada et al. underlined these findings show that “immune system monitors non-contiguous peptide sequence generated post-translationally. This capability represents an enormous increase in the ability of CTLs to recognize self and forein proteins… In a broder context, the existence of protein splicing in vertebrates greatly increases the cell’s options for converting genetic information into proteins” (Hanada et al. 2004). These results also show that protein splicing is a powerful mechanism allowing realization of the main principle of protein multifunctionality—transformation of compact genetic information into wide diversity of protein structures and functions. “Processes such as DNA recombination and RNA splicing were already known to increase the number of different proteins produced by each of mammalian genes; the discovery of protein splicing adds to the tool kit” (Rammensee, 2004).

## III. Multifunctionality as Intrinsic Feature of Different Types of Proteins

In the previous section we have described some of the mechanisms by which the Nature developes multifunctionality of the protein molecules. Below, we present examples of proteins belonging to different groups that demonstrate high extent of multifunctionality.

### 1. Amino-acyl tRNA synthetases

Amino-acyl tRNA synthetases, an ancient conserved family of proteins well known for its participation in protein synthesis, are a good paradigm of protein multifunctionality. Several studies have described numerous examples of these “housekeeping” proteins taking part in extensive critical cellular activities other than protein synthesis. These are cellular fidelity, cytokine-like activity, angiogenesis, RNA splicing, RNA trafficking, apoptosis, transcriptional and translational regulation. Amino-acyl tRNA synthetases can even block protein synthesis as established by the recent studies performed with glutamyl-prolyl-tRNA synthetase (Glu-ProRS) (Otani et al. 2002; Sampath et al. 2004; Wakasugi and Schimmel, 1999).

The lysil-tRNA synthetase (LysRS) has been shown to have a dual functionality. In addition to its contribution to the translation process, LysRS also function as a positive regulator of MITF and USF2 transcription factors via the synthesis of Ap4A (Lee et al. 2004; Lee and Razin, 2005; Plateau and Blanquet, 1982; Yannay-Cohen and Razin, 2006; Zamecnik et al. 1966).

Tyrosyl-tRNA synthetase and tryptophanil-tRNA synthetase upon their release in intracellular environment become proinflammatory cytokines with multiple activities during apoptosis, angiogenesis and inflammation. In addition, these proteins play important role in cancer progression, modulation tumor angiogenesis and its escape from surveillance by immune system (Ivakhno and Kornelyuk, 2004).

### 2. Cell-surface receptors

#### a) MUC1 protein

The MUC1 is a mucin-like type I transmembrane glycoprotein normally expressed on the surface of epithelial, hematopoetic and some other cells (Baruch et al. 1997; Weiss et al. 1991; Zaretsky et al. 1990). It is generally accepted that MUC1 gene is transcriptionaly regulated (Zaretsky et al. 2006; Zaretsky et al. 1999). It is transcribed as a single pre-mRNA followed by alternative splicing leading to synthesis of isoform specific polypeptide chains. Several MUC1 isoforms, MUC1/TM, MUC1/X, MUC1/Y, MUC1/Z and MUC1/SEC, have been identified (Tsarfaty et al. 1990; Williams et al. 1990; Wreschner et al. 1990; Zrihan-Licht et al. 1994). MUC1/TM and MUC1/X are synthesized by translation of alternativly spliced mRNAs as single polypeptides that undergo auto-cleavage while in the endoplasmic reticulum to yield the amino-terminal extracellular α-subunit, which in case of MUC1/TM contains O-glycosylated tandem repeats of 20 amino acids, and the carboxyl-terminal β-subunit, containing the membrane anchor (transmembrane domain) and cytoplasmic tail (Altschuler et al. 2000; Hilkens and Buijs, 1988; Kinlough et al. 2004; Ligtenberg et al. 1992; Linsley et al. 1988; Litvinov and Hilkens, 1993; Zrihan-Licht et al. 1994). The resulting subunits remain tightly associated with each other composing the MUC1/TM and MUC1/X isoforms, respectively.

The MUC1/TM and MUC1/X contain the SEA domain which is responsible for the self-cleavage of the precursor protein molecules leading to development of α and β subunits. The MUC1/Y isoform also contains SEA domain, however, it lakcs of 18 amino acids present in SEA domain of the MUC1/X isoform. In contrast to MUC1/X, the MUC1/Y is expressed as a single non-cleaved nascent polypeptide chain (Levitin et al. 2005b; Wreschner et al. 2002).

Translation of the MUC1/Z isoform is associated with frame shifting (Levitin et al. 2005a). The translation initiation of the MUC1 secreted form, MUC1/SEC, begins with the same AUG codon as other MUC1 isoforms, however, its termination stop codon is located in the first intron (Smorodinsky et al. 1996; Wreschner et al. 1990). The described structural peculiarities of MUC1 show its structural diversity which supposes also multiple different functions. Indeed, MUC1/TM, MUC1/X and MUC1/Y are expressed in plasma membrane and suppose to function as membrane receptors which cytoplasmic domains participate in different signal transduction pathways by interaction with the pathway specific transducer molecules (Li et al. 1998; Li et al. 2003a; Li et al. 2001a; Li et al. 2001b; Li et al. 2003b; Ren et al. 2002; Singh and Hollingsworth, 2006; Wen et al. 2003; Wreschner et al. 1994). It has been shown that besides participating in mucin forming layer, MUC1/SEC functions as ligand for MUC1/Y (Baruch et al. 1999). MUC1/Z demonstrates structural homology with some cytokins suggesting participation in defense inflammation reactions. Recently, it has been established that Hkr1 and Msb2, the yeast analogs of human transmembrane mucins, are putative osmosensors (Tatebayashi et al. 2007). These results indicate on possible new function of the MUC1.

#### b) LDL

The members of the low-density lipoprotein (LDL) receptor family are evolutionary conserved cell-surface receptors produced by mammals and other organisms (Nykjaer and Willnow, 2002). Initially thought to be endocytic receptors that exclusively mediate the uptake of lipoproteins (Goldstein, 2001; Willnow et al. 1994), recent studies in various experimental systems including knockout mice and patients with receptor gene defects uncovered a plethora of additional activities performed by these receptors (Gliemann, 1998; Gotthardt et al. 2000; Herz and Strickland, 2001). The prototype of the gene family is the LDL receptor, an endocytic receptor that mediates cellular uptake of cholesterol-rich lipoproteins. The LDL receptor like other members of this family is built by several functionally different domains which may interact with numerous of cellular proteins. These domains are represented by EGF precursor homology domain, complement-type repeat domain, *O*-linked sugar domain and NPxYmotif. Among other molecules, these domains bind to proteases, protease inhibitors, signaling molecules, heat-shock proteins, vitamin carriers, toxins and antibiotics (Basu et al. 2001; Gliemann, 1998; Herz and Strickland, 2001; Howell and Herz, 2001). These receptors physiscally or functionally interact with other classes of cell-surface proteins such as seven-transmembrane-span receptors, ion channels, glycosylphosphatidylinositol (GPI)-anchored proteins and adhesion molecules, thereby acquiring activities not usually observed for endocytic receptors. Binding of ligands not only results in endocytic uptake but also affects many cellular functions, including migration, Ca-influx, trans-cytosis, protein processing, antigen presentation, signal transduction, synaptic plasticity and cholesterol and vitamin homeostasis (Nykjaer and Willnow, 2002).

### 3. Proteases

Proteases compose one of the biggest groups of proteins. Approximately 2% of mammalian genes encode proteases (Kaiserman et al. 2006)[179]. Comparative genomics reveals that those involved in immunity and reproduction show the most inter-species diversity and multifunctionality selected during evolution. Granzymes, the cytotoxic serine proteases used by cytotoxic lymphocytes to destroy virus-infected and malignant cells, are the examples of this protein group.

There are 5 granzyme genes in humans and 10 in mice, and it is suggested that granzymes have evolved through gene duplication. Although high sequence homology and conserved primary cleavage specificity of human and mouse granzymes were observed, they are structurally and functionally divergent (Bell et al. 2003; Kaiserman et al. 2006).

Human granzyme B (GrB) has multiple paralogues in the mouse and rat, suggesting that the roles fulfilled by a single enzyme in humans may be split among several in rodents (Pham et al. 1996; Revell et al. 2005). Although functioning primarily as a cytotoxin, GrB has other roles also. In humans it is found in testis and has been implicated in reproduction (Hirst et al. 2001). It is also involved in extracellular matrix remodeling (Buzza et al. 2005).

### 4. Protein kinases

Numerous protein kinases regulate multiple pathways of signal transduction. For instance, human platelets express at least six protein kinase C (PKC) isoforms which phosphorylate multiple proteins during platelet activation (Quinton et al. 2002; Tsukuda et al. 1988). During hemostasis and thrombosis, platelets have two distinct but additive functions in thromboformation, aggregation and procoagulant activity. Recently, the stimulating and inhibitory properties of all PKC isoforms in the thrombus-forming process have been studied. It was found that platelet PKC isoforms have a dual controlling role in thrombus formation as follows: (1) by mediating secretion and integrin activation required for platelet aggregation under blood flow, and (2) by suppressing Ca^2+^-dependent phosphatidylserine exposure, and consequently thrombin generation and coagulation. Thus, platelet PKC is the signaling protein that balances the pro-aggregatory and procoagulant functions of thrombi (Strehl et al. 2007).

### 5. Cholinesterases

Cholinesterases A and B, AChE and BChE, are two enzymes which classical roles are “cholinergic functions” that originate from their cholinolytic capacity (Layer et al. 2005). However, recently non-classical functions of these enzymes have been uncovered (Johnson and Moore, 2004; Layer et al. 1993; Paraoanu and Layer, 2004). A high degree of sequence homology between AChE and a family of synaptic cell adhesion proteins, e.g. neuroligins, suggested that AChE may act also as a heterophilic cell adhesion molecule. Indeed, AChE was found to bind laminin-β1, an extracellular matrix protein involved in neuronal differentiation and adhesion. AChE binds to the laminin-β1 chain and through it can activate integrin receptor which in its turn activates intracellular signaling pathways. Thus, the complexing of AChE with laminin-1 represents a major signaling mechanism of AChE in cell formation process and pathfinding (Layer et al. 2005).

Besides of the described functions, both types of cholinesterases exhibit also aryl acylamidase activities (called AAA-AChE and AAA-BChE, respectively), which have been suggested to be involved in developmental processes (Layer et al. 2005).

### 6. Deglycosylation enzymes

Deglycosylation enzemes represents of group of proteins that catalyze separation of peptide component of glycoprotein from its carbohydrate part. As a paradigm of polyfunctional member of this group we chose the peptide: N-glycanase (PNGase). PNGase releases intact N-glycans from misfolded glycopeptides or glycoproteins which undergo deglycosylation during their degradation (Suzuki et al. 2002; Suzuki et al. 2007). The PNGase is a member of the transglutaminase (TGase) superfamily (Makarova et al. 1999). The orthologues of this enzyme are widely distributed throughout eukaryotes. While the “core” TGase domain is well conserved, a key difference is that orthologoues in higher eukaryotes have additional domains at both N- and C-termini of the core domain. The PNGase of *S. cerevisiae* contains only TGase/PNGase domain, while *C. elegans* enzyme in addition to TGase domain has thioredoxin domain and Man-binding domain located N- and C-terminally, respectively. *S. cerevisiae* possesses only deglycosylation activity whereas PNGase from *S. elegans* exhibits dual enzyme functions, not only as PNGase but also as an oxidoreductase (thioredoxin). Thus, structural changes associated with additional domains exhibited additional function to modified molecule.

### 7. Growth factors

Growth facrors are typically multifunctional proteins. Two examples will illuctrate this notion. FGFs are heparin-binding multifunctional growth factors which play a crucial role in a variety of developing processes (Bohlen, 1989). FGF-2 is one of the currently known members of the fibro-blast growth factor family. It has been established that FGF-2 functions as 1) a differentiation factor for sympathoadrenal progenitor cells; 2) a target-derived neurotrophic factor for the preganglionic sympathetic neurons of the spinal cord and 3) an element of the auto−/−paracrine chain in the adrenal medulla (Grothe and Meisinger, 1997).

Growth-colony stimulating factor (G-CSF), named for its specific stimulation of the growth of neutrophil progenitor cells *in vitro* (Roberts, 2005), is a growth factor known as a major extracellular regulator of haemopoesis and innate immune system. G-CSF influences the survival, proliferation and differentiation of all cells in the neutrophil lineage, from haemopoetic stem cell to mature neutrophils. Recent researches uncovered initially unsuspected polyfunctionality for G-CSF. This growth factor is well recognized now as a signaling molecule as well as mobiliser of haemopoetic stem cells from bone marrow into the blood. Beside that G-CSF influences T-cell function and dendritic cell activation (Roberts, 2005).

### 8. Cytokines/chemokines

Chemokines are small cytokines that function in immune responses, wound healing, inflammation and tumorogenesis (Sorg, 1989). This multifunctionality has been attributed primarily to ligand interaction with multiple receptors. Recent studies have shown that their multifunctionality could also result from interactions of the receptors with small peptides produced by precessing of the chemokines. Li et al. (Li et al. 2004) have analyzed the interactions between the two forms of human interleukin 8 (hIL8), N- and C-peptides, with the chemokine receptors hCXCR1 and hCXCR2 and showed that the N and C termini of the chemokine could stimulate the respective CXCR1 to induce intracellular Ca^2+^ release and MAPK activation independent of the other regions of molecule. These peptides could also stimulate chemotaxis of several cell types and this function is specific and mediated by hCXCR1 and/or hCXCR2. These findings advance understanding of the multifunctionality exhibited by chemokines.

### 9. Growth hormone

The growth hormone (GH) is a large multifunctional protein. It exhibits extensive heterogeneity. Some GH molecules/fragments are generated and secreted directly from the pituitary gland without any post-translational modification, whereas other molecules or fragments do arise from post-synthetic modifications (De Palo et al. 2006; Lewis et al. 2000; Wood, 2001). The link between molecular structures (different fragments of the GH-molecule) and biological effects were studied and analysis of functional activities possessing by different fragments of the GH molecule confirmed this statement.

It has been shown that the GH1-43 a.a. fragment possesses an important insulin-like activity, but does not appear to have growth-promoting activity. The insulin-like action of this amino-terminal segment has been confirmed in a series of GH peptide fragments including GH1-20 a.a., GH1-15 a.a. and GH6-13 a.a. All these GH fragments have been demonstrated to induce hypoglycaemia *in vivo* and amplify the actions of insulin *in vitro* (Ohkura and Hori, 2000).

GH44-191 a.a. fragment demonstrates neither growth-promoting nor insulin-like activities. On the contrary, it possesses GH antagonist activity (Clerico et al. 2000; Heffernan et al. 2000). GH108-129 a.a. fragment appears to possess mitogenic activity although intact GH demonstrates anti-mitogenic activity (De Palo et al. 2006). These data evidently demonstrate that different domains of the same protein molecule possess different and sometimes opposite functions.

### 10. Lactoferrin

Lactoferrin, a component of mammalian milk, is a member of the transfer proteins family (Brock, 2002; Kanyshkova et al. 2001). These glycoproteins transfer Fe^3+^ ions (Brock, 2002; Suzuki and Lonnerdal, 2002). Lactoferrin is a polyfunctional protein that influences cell proliferation and differentiation (Baveye et al. 1999). It regulates also granulopoiesis and DNA synthesis. Lactoferrin inhibits prostaglandin synthesis in human milk macrophages and activates the nonspecific immune response by stimulating phagocytosis and complement (Baveye et al. 1999; Mann et al. 1994; Ward et al. 2002). It interacts with macromolecules such as DNA, RNA, proteins, polysaccharides and heparin-like polyanions (Elass-Rochard et al. 1995; He and Furmanski, 1995; Son et al. 2002). In some cells lactoferrin functions as transcription factor (He and Furmanski, 1995; Mariller et al. 2007). It was recently demonstrated that lactoferrin also possesses ribonuclease activity (Kanyshkova et al. 2001).

### 11. Protein synthesis regulatory proteins

Protein synthesis regulatory proteins as well as proteins of other groups demonstrate extensive polyfunctionality. Sometimes, the functions they possess are unrelated or even opposite. As a paradigm of these proteins we will discuss the iron regulatory protein 1 (IRP1). IRP1 is a cytosolic, RNA binding protein that regulates the translation and stability of mRNAs encoding proteins for iron transport, storage, and use (Walden et al. 2006). IRP1 has an alternate function as cytosolic (c)-aconitase when the iron sulfur [4Fe-4S] cluster is bound (Eisenstein, 2000; Henderson and Kuhn, 1995; Hentze and Kuhn, 1996; Hentze et al. 2004; Kennedy et al. 1992; Theil and Eisenstein, 2000). The distribution of IRP1 between these mutually exclusive functions requires no new protein synthesis; iron excess promotes (c)-aconitase activity while starvation activates RNA binding function (Henderson and Kuhn, 1995). Assembly and disassembly of the iron-sulfur cluster appears to be effective mechanism for regulation IRP1 activity, dependnt on facile interchange between the two functional conformations.

The RNA-binding and enzyme active sites are extensively overlapped in IRP1, with many amino acids serving important, but different, roles in each functional state of the protein. The functional plasticity of amino acids serving both catalytic and RNA binding roles reflects the conformational flexibility of the protein, particularly in the vicinity of the Fe-S and RNA binding sites (Walden et al. 2006).

### 12. Transcriptional factors

Proteins regulating transcription belong to a huge group of DNA binding proteins. Zinc-finger containing proteins represent one of the subgroups of these proteins. The WT1, zinc-finger containing transcription factor, is a multifunctional protein. Two allels of WT1 gene is described: one contains insertion of three amino acids (+KTS) immediately after the zinc-finger 3b and another which does not contain this insertion (−KTS). In its transcriptional roles the WT1(−KTS) acts as an activator (Lee et al. 1999) while WT1(+KTS) functions as a repressor of transcription (Loeb et al. 2002). The WT1(−KTS) also binds RNA and interacts with SF1 (steroidogenic factor nuclear hormone receptor). On the contrary, the WT1(+KTS) weakly interact with SF1 but strongly binds to U2snRNP-associated splice factor (Ladomery and Dellaire, 2002).

Another zink-finger protein coding gene, ZNF74, can produce multiple protein isoforms through alternative promoter usage and splicing: these exhibit differences in their transcriptional activities and nuclear partitioning (Cote et al. 2001). The original isoform, ZNF74-I, lacks the canonical N-terminal sequences of KRAB box A, localizes to speckle domains and exhibits weak transcriptional repression. In contrast to this, the ZNF74-II isoform contains the full KRAB A and B boxes, exhibits strong transcriptional repression and localizes in a diffuse pattern throughout the nucleus (Ladomery and Dellaire, 2002).

The presented data show that these and probably other Zn-finger proteins possess functional versitality of the zinc finger motif and suggest that both alternative splicing and sub-cellular compartmentalization may modulate their multifunctionality.

### 13. Bacterial toxins

Bacterial toxins represent interesting examples of protein multifunctionality. Many toxins that act intracellularly have multidomain organization, with each domain capable of mediating a separate step of the intoxication process. There are numerous different mechanisms by which a bacterial toxin can produce pleiotropic cellular effects. On the basis of their ability to cause pleotropic cellular effects, bacterial toxins may be regarded as multifunctional proteins (Cover and Blanke, 2005).

Two of the most extensively studied examples of multifunctional bacterial toxins are ExoS and ExoT from *Pseudomonas aeruginosa*. These toxins have multiple effector domains, each of which is associated with a discrete activity against the host cell. The N-terminal domains of ExoS and ExoT are GTPase-activating proteins (GAPs) that target members of the Rho family of small GTPases, which regulate processes in cytoskeleton (Goehring et al. 1999). The C-terminal domains are ADP-rybosyltransferases that modify multiple host proteins, including Ras and Ras-like GTPases (Vincent et al. 1999). The discrete effector domains of these toxins may retain functional activity if expressed as individual proteins. The RhiGAP activities of EXoS and ExoT are almost identical, whereas ExoS and ExoT ADP-ribosylation effects are directed to different substrates (Barbieri and Sun, 2004).

Another example of a multifunctional toxin is the binary toxin produced by *Bacillus anthracis*. The B subunit of this toxin (protective antigen) can translocate two different A subunits into host cells. The A subunits may function as a metalloproteinase known as lethal factor or as adenylate cyclase known as oedema factor. On entry into host cells, lethal factor can cause many different effects, including alteration of dendritic-cell functions, repression of glucocorticoid-receptor transactivation, lysis of macrophages and apoptosis (Agrawal et al. 2003; Friedlander, 1986; Kirby, 2004).

The VacA toxin of *Helicobacter pylori* also possesses multiple functions producing upon entry into the cell: alterations in late endosomes, alterations in mitochondrial membrane permeability and inhibition of T-cell proliferation (Cover and Blanke, 2005).

Thus, many bacterial toxins are multifunctional proteins. By producing toxins with multifunctional properties, bactera are able to use a single protein to produce a range of effects at different sites in the host, depending on which cell types and tissues are targeted. Consolidating multiple functions within a single protein has several advantages to the bacteria, including conservation of genome space, conservation of energy and resources that would be needed to regulate, produce and secrete multiple gene products, and stoichiometric, special, and temporal control of multiple host-modulating activities.

### 14. Plant ABC transporters

The ABC-transporter superfamily is one of the largest protein families known. Its members can be found in bacteria, fungi, plants and animals (Henikoff et al. 1997; Martinoia et al. 2002). All ABC transporters consisit of two pairs of basic structural elements: integral membrane-spanning domains and nucleotide-binding folds oriented towards the cytoplasm. In bacteria, most members of this family are responsible for the high-affinity uptake of small molecules (for example, maltose and histidine) (Higgins, 1992). Eukaryotic ABC transporters mediate diverse cellular transport processes, such as excretion of potentially toxic compounds, lipid translocation, excretion of the mating factor of yeast, conferring heavy-metal tolerance, multidrug resistance, and antigen presentation, as well as exhibiting ion channel activity (Balzi and Goffeau, 1994; de Souza et al. 2000; Gottesman and Pastan, 1993; Higgins, 1995; Kelly et al. 1992; Kuchler et al. 1989; McGrath and Varshavsky, 1989; Szczypka et al. 1994). The first reports on plant ABC transporters showed that they are implicated in detoxification processes. Recent results indicate that the function of this protein family is not restricted to detoxifixation. Plant ABC transporters have been demonstrated to participate in chlorophyll biosynthesis, formation of Fe/S clusters, stromal movement and ion fluxes. They may also transport steroids and participate in signal transduction. Hence, plant ABC transporters play multifunctional central role in plant growth and development (Higgins, 1992).

### “Hidden” protein functions

The list of the multifunctional proteins is not limited to the described examples and could be extended. The multifunctionality appears to be a general feature of proteins. It is most likely, that in cases where proteins exhibit monofunctionality, the other functions of a protein have simply not jet been identified. These are “hidden” functions.

The “hidden or eclipsed” function is not a rear phenomenone. Many causes may lead to situation when one or several protein isoforms can not be detected while they were synthesized and present in the cell. One such a cause is a very short half-life time of some mRNA and/or protein. Another cause is the efficiency of the mRNA detection methods. The generally used analysis of expressed sequence tags (ESTs) is not perfect because the EST collection does not represent all protein coding sequences and for most genes ESTs cover only a portion of the transcripts. For instance, only half of the genes located on human chromosome 22 are represented in the current EST database (Dias Neto et al. 2000). Moreover, 26% and 65% of the ESTs correspond to the extreme 5′ and 3′ ends, respectively, of any given transcript (Graveley, 2001; Regev-Rudzki and Pines, 2007). Because much of the functionally significant alternative splicing occurs in the coding region of the transcript, many alternative splicing events might be overlooked by EST comparisons (Graveley, 2001).

Speaking about the causes leading to “loss of protein functions”, one should consider the phenomenone of dual- or multi-targeting of proteins and its consequence. The precise subcellular localization of a protein is ctitical for its function. Eukaryotic cells synthesize thousands of proteins, each having a specific function in a specific subcellular compartment. In recent years, a growing number of examples of single genes whose products are located to two (or more) separate compartments have been discovered (Huh et al. 2003; Karniely and Pines, 2005; Mackenzie, 2005).

There is a growing number of studies designed to develop a global screens of protein localization in order to characterize location-specific function (Kumar et al. 2002; Regev-Rudzki et al. 2005; Yook et al. 2004). However, these “large-scale” analyses often fail to detect one of the isoproteins in one of the locations of a dual targeted protein due to a highly uneven distribution between compartments. In frame of this phenomenon called by N. Regev-Rudzki and O. Pines ‘eclipsed distribution’ (Regev-Rudzki and Pines, 2007), the relatively large amount of an isoprotein in one subcellular compartment obscures the detection of the small amount of the other isoprotein in the other location (Levay and Viljoen, 1995).

As underlined by Regev-Rudzki and Pines (Regev-Rudzki and Pines, 2007), ‘such eclipsed distribution phenomena have a wider occurance than recorded currently; the reason being that, in most cases, the small fraction of one of the isoproteins, in one of the locations, makes its detection very difficult. This is particularly true for the cytosol which occupies several fold larger volume than other compartments thus diluting a significant amount of a dual targeted protein, so that it is easily missed by the currently used large-scale visualization methods’.

## Conclusion

The data presented in this review evidently show that genetic information embedded in nucleotide sequences of a genome contains a program of multiple structural and functional transformations of this information into real protein molecules that are involved in mirriads of reactions which ultimately determine physiology of a cell. Moreover, the encoded physico-chemical potentials of the molecules allows intermolecular interactions in such a way that one molecules may modify the other molecules thus increasing structural and functional diversity of the proteins produced by a given gene. After all, thousands of protein molecules that build the multiple mechanisms responsible for protein synthesis and different post-translational modifications are encoded in a compact form in cell genome. Hence, the cell may be considered as a very economic self-coding and self-realizing system which effectivity is beeing achieved by usage of multifunctional protein molecules. Thus, the multifunctionality is not an exceptional feature of a particular protein but to the contrary it is a fundamental basic property of most or all proteins which allows the cell effective accomplishment of multiple and complex physiological processes.

## Figures and Tables

**Table 1:** Post-translational modifications.

**Endoproteolytic cleavages:** Dibasic sites Monobasic sites Post(poly) Clu/Asp sites Post Phe-sites Tri and tetrabasic sites
**Exoproteolytic trimmings:** Carboxyamidation Glutaminyl cyclation N-terminal dipeptidyl cleavage
**Amino acid derivatizations:** Acylations Disulfide bridgings Gamma carboxylation Glycosylations Isomerisatios Phosphorylations Sulfations

## References

[b1-tog-2008-099] Acconcia F Ascenzi P Bocedi A Spisni E Tomasi V Trentalance A Visca P Marino M 2005 Palmitoylation-dependent estrogen receptor alpha membrane localization: regulation by 17beta-estradiol Mol. Biol. Cell 16 231 7 15496458 10.1091/mbc.E04-07-0547 PMC539167

[b2-tog-2008-099] Adamski FM Donly BC Tate WP 1993 Competition between frameshifting, termination and suppression at the frameshift site in the Escherichia coli release factor-2 mRNA Nucleic Acids Res 21 5074 8 7504811 10.1093/nar/21.22.5074 PMC310619

[b3-tog-2008-099] Agrawal A Lingappa J Leppla SH Agrawal S Jabbar A Quinn C Pulendran B 2003 Impairment of dendritic cells and adaptive immunity by anthrax lethal toxin Nature 424 329 34 12867985 10.1038/nature01794

[b4-tog-2008-099] Ahmad I Hoessli DC Gupta R Walker-Nasir E Rafik SM Choudhary MI Shakoori AR 2007 In silico determination of intracellular glycosylation and phosphorylation sites in human selectins: implications for biological function J. Cell Biochem 100 1558 72 17230456 10.1002/jcb.21156

[b5-tog-2008-099] Allen SH Bennett JA Mizejewski GJ Andersen TT Ferraris S Jacobson HI 1993 Purification of alpha-fetoprotein from human cord serum with demonstration of its antiestrogenic activity Biochim. Biophys. Acta 1202 135 42 7690596 10.1016/0167-4838(93)90074-2

[b6-tog-2008-099] Altschuler Y Kinlough CL Poland PA Bruns JB Apodaca G Weisz OA Hughey RP 2000 Clathrin-mediated endocytosis of MUC1 is modulated by its glycosylation state Mol. Biol. Cell 11 819 31 10712502 10.1091/mbc.11.3.819 PMC14813

[b7-tog-2008-099] Anfinsen CB 1973 Principles that govern the folding of protein chains Science 181 223 30 4124164 10.1126/science.181.4096.223

[b8-tog-2008-099] Aoyagi Y Ikenaka T Ichida F 1979 alpha-Fetoprotein as a carrier protein in plasma and its bilirubin-binding ability Cancer Res 39 3571 4 89900

[b9-tog-2008-099] Apic G Huber W Teichmann SA 2003 Multi-domain protein families and domain pairs: comparison with known structures and a random model of domain recombination J. Struct. Funct. Genomics 4 67 78 14649290 10.1023/a:1026113408773

[b10-tog-2008-099] Arai R Wriggers W Nishikawa Y Nagamune T Fujisawa T 2004 Conformations of variably linked chimeric proteins evaluated by synchrotron X-ray small-angle scattering Proteins 57 829 38 15390267 10.1002/prot.20244

[b11-tog-2008-099] Atkins JF Baranov PV Fayet O Herr AJ Howard MT Ivanov IP Matsufuji S Miller WA Moore B Prere MF Wills NM Zhou J Gesteland RF 2001 Overriding standard decoding: implications of recoding for ribosome function and enrichment of gene expression Cold Spring Harb Symp. Quant. Biol 66 217 32 12762024 10.1101/sqb.2001.66.217

[b12-tog-2008-099] Aussel C Negrel R 1986 Effect of alpha-fetoprotein on arachidonic acid metabolism in the preadipocyte cell line OB. 17 Prostaglandins Leukot. Med 21 69 76 2419923 10.1016/0262-1746(86)90164-2

[b13-tog-2008-099] Baird SD Turcotte M Korneluk RG Holcik M 2006 Searching for IRES Rna 12 1755 85 16957278 10.1261/rna.157806 PMC1581980

[b14-tog-2008-099] Balbach JAS FX Pain R 2000 Mechanisms of protein folding 212 249 IRL Press Oxford

[b15-tog-2008-099] Balzi E Goffeau A 1994 Genetics and biochemistry of yeast multi-drug resistance Biochim. Biophys. Acta 1187 152 62 8075109 10.1016/0005-2728(94)90102-3

[b16-tog-2008-099] Barbato G Ikura M Kay LE Pastor RW Bax A 1992 Backbone dynamics of calmodulin studied by 15N. relaxation using inverse detected two-dimensional NMR. spectroscopy: the central helix is flexible Biochemistry 31 5269 78 1606151 10.1021/bi00138a005

[b17-tog-2008-099] Barbieri JT Sun J 2004 Pseudomonas aeruginosa ExoS and ExoT Rev. Physiol. Biochem. Pharmacol 152 79 92 15375697 10.1007/s10254-004-0031-7

[b18-tog-2008-099] Baruch A Hartmann M Yoeli M Adereth Y Greenstein S Stadler Y Skornik Y Zaretsky J Smorodinsky NI Keydar I Wreschner DH 1999 The breast cancer-associated MUC1 gene generates both a receptor and its cognate binding protein Cancer Res 59 1552 61 10197628

[b19-tog-2008-099] Baruch A Hartmann M Zrihan-Licht S Greenstein S Burstein M Keydar I Weiss M Smorodinsky N Wreschner DH 1997 Preferential expression of novel MUC1 tumor antigen isoforms in human epithelial tumors and their tumor-potentiating function Int. J. Cancer 71 741 9 9180140 10.1002/(sici)1097-0215(19970529)71:5<741::aid-ijc9>3.0.co;2-r

[b20-tog-2008-099] Basu S Binder RJ Ramalingam T Srivastava PK 2001 CD91 is a common receptor for heat shock proteins gp96, hsp90, hsp70, and calreticulin Immunity 14 303 13 11290339 10.1016/s1074-7613(01)00111-x

[b21-tog-2008-099] Baveye S Elass E Mazurier J Spik G Legrand D 1999 Lactoferrin: a multifunctional glycoprotein involved in the modulation of the inflammatory process Clin. Chem. Lab. Med 37 281 6 10353473 10.1515/CCLM.1999.049

[b22-tog-2008-099] Behm-Ansmant I Kashima I Rehwinkel J Sauliere J Wittkopp N Izaurralde E 2007 mRNA quality control: an ancient machinery recognizes and degrades mRNAs with nonsense codons FEBS Lett 581 2845 53 17531985 10.1016/j.febslet.2007.05.027

[b23-tog-2008-099] Bell JK Goetz DH Mahrus S Harris JL Fletterick RJ Craik CS 2003 The oligomeric structure of human granzyme A is a determinant of its extended substrate specificity Nat. Struct. Biol 10 527 34 12819769 10.1038/nsb944

[b24-tog-2008-099] Berry MJ Banu L Chen YY Mandel SJ Kieffer JD Harney JW Larsen PR 1991a Recognition of UGA as a selenocysteine codon in type I deiodinase requires sequences in the 3′ untranslated region Nature 353 273 6 1832744 10.1038/353273a0

[b25-tog-2008-099] Berry MJ Banu L Larsen PR 1991b Type I iodothyronine deiodinase is a selenocysteine-containing enzyme Nature 349 438 40 1825132 10.1038/349438a0

[b26-tog-2008-099] Bijlmakers MJ Marsh M 2003 The on-off story of protein palmitoylation Trends Cell Biol 13 32 42 12480338 10.1016/s0962-8924(02)00008-9

[b27-tog-2008-099] Birkenmeier G Usbeck E Saro L Kopperschlager G 1983 Triazine dye binding of human alpha-fetoprotein and albumin J. Chromatogr 265 27 35 6194171 10.1016/s0021-9673(01)96695-6

[b28-tog-2008-099] Black DL 2003 Mechanisms of alternative pre-messenger RNA splicing Annu. Rev. Biochem 72 291 336 12626338 10.1146/annurev.biochem.72.121801.161720

[b29-tog-2008-099] Bloom JW Madanat MS Ray MK 1996 Cell. line and site specific comparative analysis of the N.-linked oligosaccharides on human ICAM-1des454-532 by electrospray ionization mass spectrometry Biochemistry 35 1856 64 8639667 10.1021/bi952354m

[b30-tog-2008-099] Bohlen P Sorg C 1989 Fibroblast growth factor Macrophage-derived cell regulatory factors Cytokines Karger Basel

[b31-tog-2008-099] Bonetti B Fu L Moon J Bedwell DM 1995 The efficiency of translation termination is determined by a synergistic interplay between upstream and downstream sequences in Saccharomyces cerevisiae J. Mol. Biol 251 334 45 7650736 10.1006/jmbi.1995.0438

[b32-tog-2008-099] Bork P Schultz J Ponting CP 1997 Cytoplasmic signalling domains: the next generation Trends Biochem. Sci 22 296 8 9270302 10.1016/s0968-0004(97)01084-0

[b33-tog-2008-099] Brierley I Pennell S 2001 Structure and function of the stimulatory RNAs involved in programmed eukaryotic-1 ribosomal frameshifting Cold Spring Harb Symp. Quant. Biol 66 233 48 12762025 10.1101/sqb.2001.66.233

[b34-tog-2008-099] Brock JH 2002 The physiology of lactoferrin Biochem. Cell Biol 80 1 6 11908632 10.1139/o01-212

[b35-tog-2008-099] Bukau B Horwich AL 1998 The Hsp70 and Hsp60 chaperone machines Cell 92 351 66 9476895 10.1016/s0092-8674(00)80928-9

[b36-tog-2008-099] Burk RF Hill KE Awad JA Morrow JD Kato T Cockell KA Lyons PR 1995 Pathogenesis of diquat-induced liver necrosis in selenium-deficient rats: assessment of the roles of lipid peroxidation and selenoprotein P Hepatology 21 561 9 7843731

[b37-tog-2008-099] Buzza MS Zamurs L Sun J Bird CH Smith AI Trapani JA Froelich CJ Nice EC Bird PI 2005 Extracellular matrix remodeling by human granzyme B. via cleavage of vitronectin, fibronectin, and laminin J. Biol. Chem 280 23549 58 15843372 10.1074/jbc.M412001200

[b38-tog-2008-099] Cai J Huang Y Li F Li Y 2006 Alteration of protein subcellular location and domain formation by alternative translational initiation Proteins 62 793 9 16342262 10.1002/prot.20785

[b39-tog-2008-099] Cai X 2007 Molecular evolution and structural analysis of the Ca(2+) release-activated Ca(2+) channel subunit, Orai J. Mol. Biol 368 1284 91 17400243 10.1016/j.jmb.2007.03.022

[b40-tog-2008-099] Candeias MM Powell DJ Roubalova E Apcher S Bourougaa K Vojtesek B Bruzzoni-Giovanelli H Fahraeus R 2006 Expression of p53 and p53/47 are controlled by alternative mechanisms of messenger RNA translation initiation Oncogene 25 6936 47 16983332 10.1038/sj.onc.1209996

[b41-tog-2008-099] Carrington DM Auffret A Hanke DE 1985 Polypeptide ligation occurs during post-translational modification of concanavalin A Nature 313 64 7 3965973 10.1038/313064a0

[b42-tog-2008-099] Casey PJ Seabra MC 1996 Protein prenyltransferases J. Biol. Chem 271 5289 92 8621375 10.1074/jbc.271.10.5289

[b43-tog-2008-099] Cesareni G 2004 Modular Protein Domains Wiley-Verlag

[b44-tog-2008-099] Chen A Kao YF Brown CM 2005a Translation of the first upstream ORF in the hepatitis B. virus pregenomic RNA modulates translation at the core and polymerase initiation codons Nucleic Acids Res 33 1169 81 15731337 10.1093/nar/gki251 PMC549565

[b45-tog-2008-099] Chen PY Lin CC Chang YT Lin SC Chan SI 2002 One O-linked sugar can affect the coil-to-beta structural transition of the prion peptide Proc. Natl. Acad. Sci. U.S.A 99 12633 8 12235358 10.1073/pnas.192137799 PMC130512

[b46-tog-2008-099] Chen YT Collins LL Uno H Chang C 2005b Deficits in motor coordination with aberrant cerebellar development in mice lacking testicular orphan nuclear receptor 4 Mol. Cell Biol 25 2722 32 15767677 10.1128/MCB.25.7.2722-2732.2005 PMC1061629

[b47-tog-2008-099] Cheng X Cole RN Zaia J Hart GW 2000 Alternative O-glycosylation/O-phosphorylation of the murine estrogen receptor beta Biochemistry 39 11609 20 10995228 10.1021/bi000755i

[b48-tog-2008-099] Cheng X Hart GW 2001 Alternative O-glycosylation/ O-phosphorylation of serine-16 in murine estrogen receptor beta: post-translational regulation of turnover and transactivation activity J. Biol. Chem 276 10570 5 11150304 10.1074/jbc.M010411200

[b49-tog-2008-099] Cheung WY 1970 Cyclic 3′, 5′-nucleotide phosphodiesterase. Demonstration of an activator Biochem. Biophys. Res. Commun 38 533 8 4315350 10.1016/0006-291x(70)90747-3

[b50-tog-2008-099] Chittum HS Himeno S Hill KE Burk RF 1996 Multiple forms of selenoprotein P in rat plasma Arch. Biochem. Biophys 325 124 8 8554336 10.1006/abbi.1996.0015

[b51-tog-2008-099] Chong S Williams KS Wotkowicz C Xu MQ 1998 Modulation of protein splicing of the Saccharomyces cerevisiae vacuolar membrane ATPase intein J. Biol. Chem 273 10567 77 9553117 10.1074/jbc.273.17.10567

[b52-tog-2008-099] Chou CF Omary MB 1994 Mitotic arrest with anti-microtubule agents or okadaic acid is associated with increased glycoprotein terminal GlcNAc’s J. Cell Sci 107 Pt 7 1833 43 7527049 10.1242/jcs.107.7.1833

[b53-tog-2008-099] Chou TY Dang CV Hart GW 1995a Glycosylation of the c-Myc transactivation domain Proc. Natl. Acad. Sci. U.S.A 92 4417 21 7753821 10.1073/pnas.92.10.4417 PMC41955

[b54-tog-2008-099] Chou TY Hart GW Dang CV 1995b c-Myc is glycosylated at threonine 58, a known phosphorylation site and a mutational hot spot in lymphomas J. Biol. Chem 270 18961 5 7642555 10.1074/jbc.270.32.18961

[b55-tog-2008-099] Clerico A Del Ry S Giannessi D 2000 Measurement of cardiac natriuretic hormones (atrial natriuretic peptide, brain natriuretic peptide, and related peptides) in clinical practice: the need for a new generation of immunoassay methods Clin. Chem 46 1529 34 11017928

[b56-tog-2008-099] Cocquerel L Duvet S Meunier JC Pillez A Cacan R Wychowski C Dubuisson J 1999 The transmembrane domain of hepatitis C virus glycoprotein E1 is a signal for static retention in the endoplasmic reticulum J. Virol 73 2641 9 10074109 10.1128/jvi.73.4.2641-2649.1999 PMC104019

[b57-tog-2008-099] Cocquerel L Meunier JC Pillez A Wychowski C Dubuisson J 1998 A retention signal necessary and sufficient for endoplasmic reticulum localization maps to the transmembrane domain of hepatitis C virus glycoprotein E2 J. Virol 72 2183 91 9499075 10.1128/jvi.72.3.2183-2191.1998 PMC109514

[b58-tog-2008-099] Cocquerel L Op de Beeck A Lambot M Roussel J Delgrange D Pillez A Wychowski C Penin F Dubuisson J 2002 Topological changes in the transmembrane domains of hepatitis C virus envelope glycoproteins Embo. J 21 2893 902 12065403 10.1093/emboj/cdf295 PMC125386

[b59-tog-2008-099] Cocquerel L Wychowski C Minner F Penin F Dubuisson J 2000 Charged residues in the transmembrane domains of hepatitis C virus glycoproteins play a major role in the processing, subcellular localization, and assembly of these envelope proteins J. Virol 74 3623 33 10729138 10.1128/jvi.74.8.3623-3633.2000 PMC111872

[b60-tog-2008-099] Cohen P Burchell A Foulkes JG Cohen PT 1978 Identification of the Ca2+-dependent modulator protein as the fourth subunit of rabbit skeletal muscle phosphorylase kinase FEBS Lett 92 287 93 212300 10.1016/0014-5793(78)80772-8

[b61-tog-2008-099] Collins LL Lee YF Heinlein CA Liu NC Chen YT Shyr CR Meshul CK Uno H Platt KA Chang C 2004 Growth retardation and abnormal maternal behavior in mice lacking testicular orphan nuclear receptor 4 Proc. Natl. Acad. Sci. U.S.A 101 15058 63 15477591 10.1073/pnas.0405700101 PMC524065

[b62-tog-2008-099] Comer FI Hart GW 2000 O-Glycosylation of nuclear and cytosolic proteins. Dynamic interplay between O-GlcNAc and O-phosphate J. Biol. Chem 275 29179 82 10924527 10.1074/jbc.R000010200

[b63-tog-2008-099] Cote F Boisvert FM Grondin B Bazinet M Goodyer CG Bazett-Jones DP Aubry M 2001 Alternative promoter usage and splicing of ZNF74 multifinger gene produce protein isoforms with a different repressor activity and nuclear partitioning DNA Cell Biol 20 159 73 11313019 10.1089/104454901300069004

[b64-tog-2008-099] Cover TL Blanke SR 2005 Helicobacter pylori VacA, a paradigm for toxin multifunctionality Nat. Rev. Microbiol 3 320 32 15759043 10.1038/nrmicro1095

[b65-tog-2008-099] Coyle JE Jaeger J Gross M Robinson CV Radford SE 1997 Structural and mechanistic consequences of polypeptide binding by GroEL Fold Des 2 R.93 104 10.1016/S1359-0278(97)00046-1 9427006

[b66-tog-2008-099] Creighton T Pain R 2002 Mechanisms of protein folding 250 278 IRL Press Oxford

[b67-tog-2008-099] Cridge AG Major LL Mahagaonkar AA Poole ES Isaksson LA Tate WP 2006 Comparison of characteristics and function of translation termination signals between and within prokaryotic and eukaryotic organisms Nucleic Acids Res 34 1959 73 16614446 10.1093/nar/gkl074 PMC1435984

[b68-tog-2008-099] Datta B Ray MK Chakrabarti D Wylie DE Gupta NK 1989 Glycosylation of eukaryotic peptide chain initiation factor 2 (eIF-2)-associated 67-kDa polypeptide (p67) and its possible role in the inhibition of eIF-2 kinase-catalyzed phosphorylation of the eIF-2 alpha-subunit J. Biol. Chem 264 20620 4 2511207

[b69-tog-2008-099] Dauphinee MJ Mizejewski GJ 2002 Human alpha-fetoprotein contains potential heterodimerization motifs capable of interaction with nuclear receptors and transcription/growth factors Med. Hypotheses 58 453 61 12323110 10.1054/mehy.2001.1445

[b70-tog-2008-099] David R Richter MP Beck-Sickinger AG 2004 Expressed protein ligation. Method and applications Eur. J. Biochem 271 663 77 14764082 10.1111/j.1432-1033.2004.03978.x

[b71-tog-2008-099] De Palo EF Gatti R Antonelli G Spinella P 2006 Growth hormone isoforms, segments/fragments: does a link exist with multifunctionality? Clin. Chim. Acta 364 77 81 16154554 10.1016/j.cca.2005.06.010

[b72-tog-2008-099] de Souza SJ Camargo AA Briones MR Costa FF Nagai MA Verjovski-Almeida S Zago MA Andrade LE Carrer H El-Dorry HF Espreafico EM Habr-Gama A Giannella-Neto D Goldman GH Gruber A Hackel C Kimura ET Maciel RM Marie SK Martins EA Nobrega MP Paco-Larson ML Pardini MI Pereira GG Pesquero JB Rodrigues V Rogatto SR da Silva ID Sogayar MC de Fatima Sonati M Tajara EH Valentini SR Acencio M Alberto FL Amaral ME Aneas I Bengtson MH Carraro DM Carvalho AF Carvalho LH Cerutti JM Correa ML Costa MC Curcio C Gushiken T Ho PL Kimura E Leite LC Maia G Majumder P Marins M Matsukuma A Melo AS Mestriner CA Miracca EC Miranda DC Nascimento AN Nobrega FG Ojopi EP Pandolfi JR Pessoa LG Rahal P Rainho CA da Ros N de Sa RG Sales MM da Silva NP Silva TC da Silva W Jr Simao DF Sousa JF Stecconi D Tsukumo F Valente V Zalcbeg H Brentani RR Reis FL Dias-Neto E Simpson AJ 2000 Identification of human chromosome 22 transcribed sequences with ORF expressed sequence tags Proc. Natl. Acad. Sci. U.S.A 97 12690 3 11070084 10.1073/pnas.97.23.12690 PMC18825

[b73-tog-2008-099] De Vit MJ Waddle JA Johnston M 1997 Regulated nuclear translocation of the Mig1 glucose repressor Mol. Biol. Cell 8 1603 18 9285828 10.1091/mbc.8.8.1603 PMC276179

[b74-tog-2008-099] Dias Neto E Correa RG Verjovski-Almeida S Briones MR Nagai MA da Silva W Jr Zago MA Bordin S Costa FF Goldman GH Carvalho AF Matsukuma A Baia GS Simpson DH Brunstein A de Oliveira PS Bucher P Jongeneel CV O’Hare MJ Soares F Brentani RR Reis LF de Souza SJ Simpson AJ 2000 Shotgun sequencing of the human transcriptome with ORF expressed sequence tags Proc. Natl. Acad. Sci. U.S.A 97 3491 6 10737800 10.1073/pnas.97.7.3491 PMC16267

[b75-tog-2008-099] Dobson CM 1990 Protein conformation. Hinge-bending and folding Nature 348 198 9 2234088 10.1038/348198a0

[b76-tog-2008-099] Dobson CM Karplus M 1999 The fundamentals of protein folding: bringing together theory and experiment Curr. Opin. Struct. Biol 9 92 101 10047588 10.1016/s0959-440x(99)80012-8

[b77-tog-2008-099] Dong DL Hart GW 1994 Purification and characterization of an O-GlcNAc selective N.-acetyl-beta-D-glucosaminidase from rat spleen cytosol J. Biol. Chem 269 19321 30 8034696

[b78-tog-2008-099] Doronina VA Brown JD 2006 Non-canonical decoding events at stop codons in eukaryotes Mol. Biol. (Mosk) 40 731 41 16913232

[b79-tog-2008-099] Draper JM Xia Z Smith CD 2007 Cellular palmitoylation and trafficking of lipidated peptides J. Lipid Res 48 1873 84 17525474 10.1194/jlr.M700179-JLR200 PMC2895159

[b80-tog-2008-099] Du XL Edelstein D Rossetti L Fantus IG Goldberg H Ziyadeh F Wu J Brownlee M 2000 Hyperglycemia-induced mitochondrial superoxide overproduction activates the hexosamine pathway and induces plasminogen activator inhibitor-1 expression by increasing Sp1 glycosylation Proc. Natl. Acad. Sci. U.S.A 97 12222 6 11050244 10.1073/pnas.97.22.12222 PMC17322

[b81-tog-2008-099] Dunphy JT Linder ME 1998 Signalling functions of protein palmitoylation Biochim. Biophys. Acta 1436 245 61 9838145 10.1016/s0005-2760(98)00130-1

[b82-tog-2008-099] Eisenstein RS 2000 Iron regulatory proteins and the molecular control of mammalian iron metabolism Annu. Rev. Nutr 20 627 62 10940348 10.1146/annurev.nutr.20.1.627

[b83-tog-2008-099] el-Husseini Ael D Bredt DS 2002 Protein palmitoylation: a regulator of neuronal development and function Nat. Rev. Neurosci 3 791 802 12360323 10.1038/nrn940

[b84-tog-2008-099] Elass-Rochard E Roseanu A Legrand D Trif M Salmon V Motas C Montreuil J Spik G 1995 Lactoferrin-lipopolysaccharide interaction: involvement of the 28–34 loop region of human lactoferrin in the high-affinity binding to Escherichia coli 055B5 lipopolysaccharide Biochem. J 312 Pt 3 839 45 8554529 10.1042/bj3120839 PMC1136191

[b85-tog-2008-099] Ellis RJ Hartl FU 1996 Protein folding in the cell: competing models of chaperonin function Faseb J 10 20 6 8566542 10.1096/fasebj.10.1.8566542

[b86-tog-2008-099] Evans TC Jr Benner J Xu MQ 1999 The in vitro ligation of bacterially expressed proteins using an intein from Methanobacterium thermoautotrophicum J. Biol. Chem 274 3923 6 9933578 10.1074/jbc.274.7.3923

[b87-tog-2008-099] Evans TJT Xu MQ 2002 Mechanistic and kinetic considerations of protein splicing Chem. Rev 102 4869 84 12475209 10.1021/cr9601369

[b88-tog-2008-099] Fearon K McClendon V Bonetti B Bedwell DM 1994 Premature translation termination mutations are efficiently suppressed in a highly conserved region of yeast Ste6p, a member of the ATP-binding cassette (ABC) transporter family J. Biol. Chem 269 17802 8 7517933

[b89-tog-2008-099] Fewell SW Travers KJ Weissman JS Brodsky JL 2001 The action of molecular chaperones in the early secretory pathway Annu. Rev. Genet 35 149 91 11700281 10.1146/annurev.genet.35.102401.090313

[b90-tog-2008-099] Filippou PS Lioliou EE Panagiotidis CA Athanassopoulos CM Garnelis T Papaioannou D Kyriakidis DA 2007 Effect of polyamines and synthetic polyamine-analogues on the expression of antizyme (AtoC) and its regulatory genes BMC Biochem 8 1 17224065 10.1186/1471-2091-8-1 PMC1784093

[b91-tog-2008-099] Finol-Urdaneta RK Struver N Terlau H 2006 Molecular and Functional Differences between Heart mKv1.7 Channel Isoforms J. Gen. Physiol 128 133 45 16801386 10.1085/jgp.200609498 PMC2151556

[b92-tog-2008-099] Frand AR Cuozzo JW Kaiser CA 2000 Pathways for protein disulphide bond formation Trends Cell Biol 10 203 10 10754564 10.1016/s0962-8924(00)01745-1

[b93-tog-2008-099] Friedlander AM 1986 Macrophages are sensitive to anthrax lethal toxin through an acid-dependent process J. Biol. Chem 261 7123 6 3711080

[b94-tog-2008-099] Frischmeyer PA Dietz HC 1999 Nonsense-mediated mRNA decay in health and disease Hum. Mol. Genet 8 1893 900 10469842 10.1093/hmg/8.10.1893

[b95-tog-2008-099] Gandre S Bercovich Z Kahana C 2003 Mitochondrial localization of antizyme is determined by context-dependent alternative utilization of two AUG initiation codons Mitochondrion 2 245 56 16120325 10.1016/S1567-7249(02)00105-8

[b96-tog-2008-099] Gasdaska PY Gasdaska JR Cochran S Powis G 1995 Cloning and sequencing of a human thioredoxin reductase FEBS Lett 373 5 9 7589432 10.1016/0014-5793(95)01003-w

[b97-tog-2008-099] Gebauer F Hentze MW 2004 Molecular mechanisms of translational control Nat. Rev. Mol. Cell Biol 5 827 35 15459663 10.1038/nrm1488 PMC7097087

[b98-tog-2008-099] Gesteland RF Atkins JF 1996 Recoding: dynamic reprogramming of translation Annu Rev Biochem 65 10.1146/annurev.bi.65.070196.003521 8811194

[b99-tog-2008-099] Gesteland RF Weiss RB Atkins JF 1992 Recoding: reprogrammed genetic decoding Science 257 1640 1 1529352 10.1126/science.1529352

[b100-tog-2008-099] Ghosh I Sun L Xu MQ 2001 Zinc inhibition of protein trans-splicing and identification of regions essential for splicing and association of a split intein* J. Biol. Chem 276 24051 8 11331276 10.1074/jbc.M011049200

[b101-tog-2008-099] Gliemann J 1998 Receptors of the low density lipoprotein (LDL) receptor family in man. Multiple functions of the large family members via interaction with complex ligands Biol. Chem 379 951 64 9792428

[b102-tog-2008-099] Goehring UM Schmidt G Pederson KJ Aktories K Barbieri JT 1999 The N-terminal domain of Pseudomonas aeruginosa exoenzyme S is a GTPase-activating protein for Rho GTPases J. Biol. Chem 274 36369 72 10593930 10.1074/jbc.274.51.36369

[b103-tog-2008-099] Gokhale RS Khosla C 2000 Role of linkers in communication between protein modules Curr. Opin. Chem. Biol 4 22 7 10679375 10.1016/s1367-5931(99)00046-0

[b104-tog-2008-099] Goldstein J Scriver C 2001 Familial hypercholesteremia Metabolic and Molecular Bases of Inherited Disease 2863 2913 McGraw-Hill

[b105-tog-2008-099] Gopinath RM Vincenzi FF 1977 Phosphodiesterase protein activator mimics red blood cell cytoplasmic activator of (Ca2+-Mg2+) ATPase Biochem. Biophys. Res. Commun 77 1203 9 197955 10.1016/s0006-291x(77)80107-1

[b106-tog-2008-099] Goto C Osaka T Mizutani T 2001 A model for Sec incorporation with the regions upstream of the UGA Sec codon to play a key role Biofactors 14 25 35 11568437 10.1002/biof.5520140105

[b107-tog-2008-099] Gottesman MM Pastan I 1993 Biochemistry of multidrug resistance mediated by the multidrug transporter Annu. Rev. Biochem 62 385 427 8102521 10.1146/annurev.bi.62.070193.002125

[b108-tog-2008-099] Gotthardt M Trommsdorff M Nevitt MF Shelton J Richardson JA Stockinger W Nimpf J Herz J 2000 Interactions of the low density lipoprotein receptor gene family with cytosolic adaptor and scaffold proteins suggest diverse biological functions in cellular communication and signal transduction J. Biol. Chem 275 25616 24 10827173 10.1074/jbc.M000955200

[b109-tog-2008-099] Graveley BR 2001 Alternative splicing: increasing diversity in the proteomic world Trends Genet 17 100 7 11173120 10.1016/s0168-9525(00)02176-4

[b110-tog-2008-099] Greaves J Chamberlain LH 2007 Palmitoylation-dependent protein sorting J. Cell Biol 176 249 54 17242068 10.1083/jcb.200610151 PMC2063950

[b111-tog-2008-099] Gritli-Linde A Nilsson J Bohlooly YM Heby O Linde A 2001 Nuclear translocation of antizyme and expression of ornithine decarboxylase and antizyme are developmentally regulated Dev. Dyn 220 259 75 11241834 10.1002/1097-0177(20010301)220:3<259::AID-DVDY1100>3.0.CO;2-#

[b112-tog-2008-099] Groisman I Engelberg-Kulka H 1995 Translational bypassing: a new reading alternative of the genetic code Biochem. Cell Biol 73 1055 9 8722021 10.1139/o95-113

[b113-tog-2008-099] Grothe C Meisinger C 1997 The multifunctionality of FGF-2 in the adrenal medulla Anat. Embryol. (Berl) 195 103 11 9045980 10.1007/s004290050029

[b114-tog-2008-099] Gu Z Cavalcanti A Chen FC Bouman P Li WH 2002 Extent of gene duplication in the genomes of Drosophila, nematode, and yeast Mol. Biol. Evol 19 256 62 11861885 10.1093/oxfordjournals.molbev.a004079

[b115-tog-2008-099] Gutsche I Essen LO Baumeister W 1999 Group II chaperonins: new TRiC(k)s and turns of a protein folding machine J. Mol. Biol 293 295 312 10550210 10.1006/jmbi.1999.3008

[b116-tog-2008-099] Haltiwanger RS Blomberg MA Hart GW 1992 Glycosylation of nuclear and cytoplasmic proteins. Purification and characterization of a uridine diphospho-N-acetylglucosamine:polypeptide beta-N-acetylglucosaminyltransferase J. Biol. Chem 267 9005 13 1533623

[b117-tog-2008-099] Haltiwanger RS Holt GD Hart GW 1990 Enzymatic addition of O-GlcNAc to nuclear and cytoplasmic proteins. Identification of a uridine diphospho-N-acetylglucosamine:peptide beta-N-acetylglucosaminyltransferase J. Biol. Chem 265 2563 8 2137449

[b118-tog-2008-099] Han I Oh ES Kudlow JE 2000 Responsiveness of the state of O-linked N.-acetylglucosamine modification of nuclear pore protein p62 to the extracellular glucose concentration Biochem. J , 350 Pt 1 109 14 10926833 PMC1221231

[b119-tog-2008-099] Hanada K Yang JC 2005 Novel biochemistry: post-translational protein splicing and other lessons from the school of antigen processing J. Mol. Med 83 420 8 15759099 10.1007/s00109-005-0652-6

[b120-tog-2008-099] Hanada K Yewdell JW Yang JC 2004 Immune. recognition of a human renal cancer antigen through post-translational protein splicing Nature 427 252 6 14724640 10.1038/nature02240

[b121-tog-2008-099] Hanke J Brett D Zastrow I Aydin A Delbruck S Lehmann G Luft F Reich J Bork P 1999 Alternative splicing of human genes: more the rule than the exception? Trends Genet 15 389 90 10498933 10.1016/s0168-9525(99)01830-2

[b122-tog-2008-099] Harrison SC 1981 Molecular organization of virus particles: implications for assembly Prog. Clin. Biol. Res 64 3 18 7330049

[b123-tog-2008-099] Hart GW 1997 Dynamic O-linked glycosylation of nuclear and cytoskeletal proteins Annu. Rev. Biochem 66 315 35 9242909 10.1146/annurev.biochem.66.1.315

[b124-tog-2008-099] Hart GW Housley MP Slawson C 2007 Cycling of O-linked beta-N-acetylglucosamine on nucleocytoplasmic proteins Nature 446 1017 22 17460662 10.1038/nature05815

[b125-tog-2008-099] Hartl FU Hayer-Hartl M 2002 Molecular chaperones in the cytosol: from nascent chain to folded protein Science 295 1852 8 11884745 10.1126/science.1068408

[b126-tog-2008-099] He J Furmanski P 1995 Sequence specificity and transcriptional activation in the binding of lactoferrin to DNA Nature 373 721 4 7854459 10.1038/373721a0

[b127-tog-2008-099] Heffernan MA Jiang WJ Thorburn AW Ng FM 2000 Effects of oral administration of a synthetic fragment of human growth hormone on lipid metabolism Am. J. Physiol. Endocrinol. Metab 279 E501 7 10950816 10.1152/ajpendo.2000.279.3.E501

[b128-tog-2008-099] Heller JS Fong WF Canellakis ES 1976 Induction of a protein inhibitor to ornithine decarboxylase by the end products of its reaction Proc. Natl. Acad. Sci. U.S.A 73 1858 62 1064859 10.1073/pnas.73.6.1858 PMC430406

[b129-tog-2008-099] Henderson BR Kuhn LC 1995 Differential modulation of the RNA-binding proteins IRP-1 and IRP-2 in response to iron. IRP-2 inactivation requires translation of another protein J. Biol. Chem 270 20509 15 7544791 10.1074/jbc.270.35.20509

[b130-tog-2008-099] Henikoff S Greene EA Pietrokovski S Bork P Attwood TK Hood L 1997 Gene families: the taxonomy of protein paralogs and chimeras Science 278 609 14 9381171 10.1126/science.278.5338.609

[b131-tog-2008-099] Henkel T Zabel U van Zee K Muller JM Fanning E Baeuerle PA 1992 Intramolecular masking of the nuclear location signal and dimerization domain in the precursor for the p50 NF-kappa B. subunit Cell 68 1121 33 1547506 10.1016/0092-8674(92)90083-o

[b132-tog-2008-099] Hentze MW Kuhn LC 1996 Molecular control of vertebrate iron metabolism: mRNA-based regulatory circuits operated by iron, nitric oxide, and oxidative stress Proc. Natl. Acad. Sci. U.S.A 93 8175 82 8710843 10.1073/pnas.93.16.8175 PMC38642

[b133-tog-2008-099] Hentze MW Muckenthaler MU Andrews NC 2004 Balancing acts: molecular control of mammalian iron metabolism Cell 117 285 97 15109490 10.1016/s0092-8674(04)00343-5

[b134-tog-2008-099] Herr AJ Atkins JF Gesteland RF 2000a Coupling of open reading frames by translational bypassing Annu. Rev. Biochem 69 343 72 10966462 10.1146/annurev.biochem.69.1.343

[b135-tog-2008-099] Herr AJ Gesteland RF Atkins JF 2000b One protein from two open reading frames: mechanism of a 50 nt translational bypass Embo. J 19 2671 80 10835364 10.1093/emboj/19.11.2671 PMC212773

[b136-tog-2008-099] Herz J Strickland DK 2001 LRP: a multifunctional scavenger and signaling receptor J. Clin. Invest 108 779 84 11560943 10.1172/JCI13992 PMC200939

[b137-tog-2008-099] Higgins CF 1992 ABC transporters: from microorganisms to man Annu Rev. Cell Biol 8 67 113 1282354 10.1146/annurev.cb.08.110192.000435

[b138-tog-2008-099] Higgins CF 1995 The ABC of channel regulation Cell 82 693 6 7671298 10.1016/0092-8674(95)90465-4

[b139-tog-2008-099] Hilkens J Buijs F 1988 Biosynthesis of MAM-6, an epithelial sialomucin. Evidence for involvement of a rare proteolytic cleavage step in the endoplasmic reticulum J. Biol. Chem 263 4215 22 3346246

[b140-tog-2008-099] Hill KE Lloyd RS Yang JG Read R Burk RF 1991 The cDNA for rat selenoprotein P contains 10 TGA codons in the open reading frame J. Biol. Chem 266 10050 3 2037562

[b141-tog-2008-099] Hinnebusch AG Natarajan K 2002 Gcn4p, a master regulator of gene expression, is controlled at multiple levels by diverse signals of starvation and stress Eukaryot. Cell 1 22 32 12455968 10.1128/EC.01.1.22-32.2002 PMC118051

[b142-tog-2008-099] Hironaka T Furukawa K Esmon PC Yokota T Brown JE Sawada S Fournel MA Kato M Minaga T Kobata A 1993 Structural study of the sugar chains of porcine factor VIII—tissue- and species-specific glycosylation of factor VIII Arch. Biochem. Biophys 307 316 30 8274017 10.1006/abbi.1993.1595

[b143-tog-2008-099] Hirst CE Buzza MS Sutton VR Trapani JA Loveland KL Bird PI 2001 Perforin-independent expression of granzyme B. and proteinase inhibitor 9 in human testis and placenta suggests a role for granzyme B-mediated proteolysis in reproduction Mol. Hum. Reprod 7 1133 42 11719590 10.1093/molehr/7.12.1133

[b144-tog-2008-099] Holcik M Sonenberg N Korneluk RG 2000 Internal ribosome initiation of translation and the control of cell death Trends Genet 16 469 73 11050335 10.1016/s0168-9525(00)02106-5

[b145-tog-2008-099] Howell BW Herz J 2001 The LDL receptor gene family: signaling functions during development Curr. Opin. Neurobiol 11 74 81 11179875 10.1016/s0959-4388(00)00176-8

[b146-tog-2008-099] Huez I Bornes S Bresson D Creancier L Prats H 2001 New vascular endothelial growth factor isoform generated by internal ribosome entry site-driven CUG translation initiation Mol. Endocrinol 15 2197 210 11731620 10.1210/mend.15.12.0738

[b147-tog-2008-099] Huez I Creancier L Audigier S Gensac MC Prats AC Prats H 1998 Two independent internal ribosome entry sites are involved in translation initiation of vascular endothelial growth factor mRNA Mol. Cell Biol 18 6178 90 9774635 10.1128/mcb.18.11.6178 PMC109205

[b148-tog-2008-099] Huh WK Falvo JV Gerke LC Carroll AS Howson RW Weissman JS O’Shea EK 2003 Global analysis of protein localization in budding yeast Nature 425 686 91 14562095 10.1038/nature02026

[b149-tog-2008-099] Huq MD Gupta P Tsai NP Wei LN 2006 Modulation of testicular receptor 4 activity by mitogen-activated protein kinase-mediated phosphorylation Mol. Cell. Proteomics 5 2072 82 16887930 10.1074/mcp.M600180-MCP200

[b150-tog-2008-099] Ichiba T Matsufuji S Miyazaki Y Murakami Y Tanaka K Ichihara A Hayashi S 1994 Functional regions of ornithine decarboxylase antizyme Biochem. Biophys. Res. Commun 200 1721 7 8185631 10.1006/bbrc.1994.1651

[b151-tog-2008-099] Ichtchenko K Hata Y Nguyen T Ullrich B Missler M Moomaw C Sudhof TC 1995 Neuroligin 1: a splice site-specific ligand for beta-neurexins Cell 81 435 43 7736595 10.1016/0092-8674(95)90396-8

[b152-tog-2008-099] Ikura M Ames JB 2006 Genetic polymorphism and protein conformational plasticity in the calmodulin superfamily: two ways to promote multifunctionality Proc. Natl. Acad. Sci. U.S.A 103 1159 64 16432210 10.1073/pnas.0508640103 PMC1360552

[b153-tog-2008-099] Ikura M Clore GM Gronenborn AM Zhu G Klee CB Bax A 1992 Solution structure of a calmodulin-target peptide complex by multidimensional NMR Science 256 10.1126/science.1585175 1585175

[b154-tog-2008-099] Isken O Maquat LE 2007 Quality control of eukaryotic mRNA: safeguarding cells from abnormal mRNA function Genes Dev 21 1833 56 17671086 10.1101/gad.1566807

[b155-tog-2008-099] Iturralde M Alava MA Gonzalez B Anel A Pineiro A 1991 Effect of alpha-fetoprotein and albumin on the uptake of polyunsaturated fatty acids by rat hepatoma cells and fetal rat hepatocytes Biochim. Biophys. Acta 1086 81 8 1720022 10.1016/0005-2760(91)90157-d

[b156-tog-2008-099] Ivakhno SS Kornelyuk AI 2004 Cytokine-like activities of some aminoacyl-tRNA synthetases and auxiliary p43 cofactor of amino-acylation reaction and their role in oncogenesis Exp. Oncol 26 250 5 15627054

[b157-tog-2008-099] Jacobson HI Bennett JA Mizejewski GJ 1990 Inhibition of estrogen-dependent breast cancer growth by a reaction product of alpha-fetoprotein and estradiol Cancer Res 50 415 20 1688512

[b158-tog-2008-099] Janzen DM Geballe AP 2004 The effect of eukaryotic release factor depletion on translation termination in human cell lines Nucleic Acids Res 32 4491 502 15326224 10.1093/nar/gkh791 PMC516063

[b159-tog-2008-099] Jarrett HW Penniston JT 1977 Partial purification of the Ca2+-Mg2+ ATPase activator from human erythrocytes: its similarity to the activator of 3′:5′—cyclic nucleotide phosphodiesterase Biochem. Biophys. Res. Commun 77 1210 6 197956 10.1016/s0006-291x(77)80108-3

[b160-tog-2008-099] Johnson G Moore SW 2004 Identification of a structural site on acetylcholinesterase that promotes neurite outgrowth and binds laminin-1 and collagen IV Biochem. Biophys. Res. Commun 319 448 55 15178427 10.1016/j.bbrc.2004.05.018

[b161-tog-2008-099] Kaiserman D Bird CH Sun J Matthews A Ung K Whisstock JC Thompson PE Trapani JA Bird PI 2006 The major human and mouse granzymes are structurally and functionally divergent J. Cell Biol 175 619 30 17116752 10.1083/jcb.200606073 PMC2064598

[b162-tog-2008-099] Kakiuchi S Yamazaki R 1970 Calcium dependent phosphodiesterase activity and its activating factor (PAF) from brain studies on cyclic 3′, 5′-nucleotide phosphodiesterase (3) Biochem. Biophys. Res. Commun 41 1104 10 4320714 10.1016/0006-291x(70)90199-3

[b163-tog-2008-099] Kanyshkova TG Buneva VN Nevinsky GA 2001 Lactoferrin and its biological functions Biochemistry (Mosc) 66 1 7 11240386 10.1023/a:1002817226110

[b164-tog-2008-099] Kapp LD Lorsch JR 2004 The molecular mechanics of eukaryotic translation Annu. Rev. Biochem 73 657 704 15189156 10.1146/annurev.biochem.73.030403.080419

[b165-tog-2008-099] Karniely S Pines O 2005 Single translation—dual destination: mechanisms of dual protein targeting in eukaryotes EMBO Rep 6 420 5 15864293 10.1038/sj.embor.7400394 PMC1299304

[b166-tog-2008-099] Karunakaran KP Noguchi Y Read TD Cherkasov A Kwee J Shen C Nelson CC Brunham RC 2003 Molecular analysis of the multiple GroEL proteins of Chlamydiae J. Bacteriol 185 1958 66 12618460 10.1128/JB.185.6.1958-1966.2003 PMC150133

[b167-tog-2008-099] Kearse KP Hart GW 1991 Lymphocyte activation induces rapid changes in nuclear and cytoplasmic glycoproteins Proc. Natl. Acad. Sci. U.S.A 88 1701 5 2000378 10.1073/pnas.88.5.1701 PMC51092

[b168-tog-2008-099] Keeling KM Lanier J Du M Salas-Marco J Gao L Kaenjak-Angeletti A Bedwell DM 2004 Leaky termination at premature stop codons antagonizes nonsense-mediated mRNA decay in S. cerevisiae Rna 10 691 703 15037778 10.1261/rna.5147804 PMC1262634

[b169-tog-2008-099] Kelly A Powis SH Kerr LA Mockridge I Elliott T Bastin J Uchanska-Ziegler B Ziegler A Trowsdale J Townsend A 1992 Assembly and function of the two ABC transporter proteins encoded in the human major histocompatibility complex Nature 355 641 4 1538751 10.1038/355641a0

[b170-tog-2008-099] Kelly WG Dahmus ME Hart GW 1993 RNA polymerase II is a glycoprotein. Modification of the COOH-terminal domain by O-GlcNAc J. Biol. Chem 268 10416 24 8486697

[b171-tog-2008-099] Kennedy MC Mende-Mueller L Blondin GA Beinert H 1992 Purification and characterization of cytosolic aconitase from beef liver and its relationship to the iron-responsive element binding protein Proc. Natl. Acad. Sci. U.S.A 89 11730 4 1334546 10.1073/pnas.89.24.11730 PMC50630

[b172-tog-2008-099] Kim E Xie S Yeh SD Lee YF Collins LL Hu YC Shyr CR Mu XM Liu NC Chen YT Wang PH Chang C 2003 Disruption of TR.4 orphan nuclear receptor reduces the expression of liver apolipoprotein E/C-I/C-II gene cluster J. Biol. Chem 278 46919 26 12954636 10.1074/jbc.M304088200

[b173-tog-2008-099] Kinlough CL Poland PA Bruns JB Harkleroad KL Hughey RP 2004 MUC1 membrane trafficking is modulated by multiple interactions J. Biol. Chem 279 53071 7 15471854 10.1074/jbc.M409360200

[b174-tog-2008-099] Kirby JE 2004 Anthrax lethal toxin induces human endothelial cell apoptosis Infect. Immun 72 430 9 14688124 10.1128/IAI.72.1.430-439.2004 PMC343952

[b175-tog-2008-099] Klabunde T Sharma S Telenti A Jacobs WRJr Sacchettini JC 1998 Crystal structure of GyrA intein from Mycobacterium xenopi reveals structural basis of protein splicing Nat. Struct. Biol 5 31 6 9437427 10.1038/nsb0198-31

[b176-tog-2008-099] Kochetov AV 2006 Alternative translation start sites and their significance for eukaryotic proteome Mol. Biol. (Mosk) 40 788 95 17086979

[b177-tog-2008-099] Kochetov AV Sarai A Rogozin IB Shumny VK Kolchanov NA 2005 The role of alternative translation start sites in the generation of human protein diversity Mol. Genet. Genomics 273 491 6 15959805 10.1007/s00438-005-1152-7

[b178-tog-2008-099] Komeili A O’Shea EK 1999 Roles of phosphorylation sites in regulating activity of the transcription factor Pho4 Science 284 977 80 10320381 10.1126/science.284.5416.977

[b179-tog-2008-099] Koritschoner NP Madruga J Knespel S Blendinger G Anzinger B Otto A Zenke M Bartunek P 2001 The nuclear orphan receptor TR.4 promotes proliferation of myeloid progenitor cells Cell. Growth Differ 12 563 72 11714637

[b180-tog-2008-099] Kozak M 1980 Influence of mRNA secondary structure on binding and migration of 40S ribosomal subunits Cell 19 79 90 7357609 10.1016/0092-8674(80)90390-6

[b181-tog-2008-099] Kozak M 2002 Pushing the limits of the scanning mechanism for initiation of translation Gene 299 1 34 12459250 10.1016/S0378-1119(02)01056-9 PMC7126118

[b182-tog-2008-099] Krebs EG 1993 Nobel Lecture. Protein phosphorylation and cellular regulation I Biosci. Rep 13 127 42 8268421 10.1007/BF01149958

[b183-tog-2008-099] Kreppel LK Blomberg MA Hart GW 1997 Dynamic glycosylation of nuclear and cytosolic proteins. Cloning and characterization of a unique O-GlcNAc transferase with multiple tetratricopeptide repeats J. Biol. Chem 272 9308 15 9083067 10.1074/jbc.272.14.9308

[b184-tog-2008-099] Kreppel LK Hart GW 1999 Regulation of a cytosolic and nuclear O-GlcNAc transferase. Role of the tetratricopeptide repeats J. Biol. Chem 274 32015 22 10542233 10.1074/jbc.274.45.32015

[b185-tog-2008-099] Kretsinger RH Nockolds CE 1973 Carp muscle calcium-binding protein. II. Structure determination and general description J. Biol. Chem 248 3313 26 4700463

[b186-tog-2008-099] Kuchler K Sterne RE Thorner J 1989 Saccharomyces cerevisiae STE6 gene product: a novel pathway for protein export in eukaryotic cells Embo. J 8 3973 84 2686977 10.1002/j.1460-2075.1989.tb08580.x PMC401572

[b187-tog-2008-099] Kumar A Agarwal S Heyman JA Matson S Heidtman M Piccirillo S Umansky L Drawid A Jansen R Liu Y Cheung KH Miller P Gerstein M Roeder GS Snyder M 2002 Subcellular localization of the yeast proteome Genes Dev 16 707 19 11914276 10.1101/gad.970902 PMC155358

[b188-tog-2008-099] Ladomery M Dellaire G 2002 Multifunctional zinc finger proteins in development and disease Ann. Hum. Genet 66 331 42 12485467 10.1017/S0003480002001215

[b189-tog-2008-099] Layer PG Allebrandt K Andermann P Bodur E Boopathy R Bytyqi AH Paraoanu LE 2005 On the multifunctionality of cholinesterases Chem Biol Interact 157–158 37 41 10.1016/j.cbi.2005.10.006 16246318

[b190-tog-2008-099] Layer PG Weikert T Alber R 1993 Cholinesterases regulate neurite growth of chick nerve cells in vitro by means of a non-enzymatic mechanism Cell Tissue Res 273 219 26 8103422 10.1007/BF00312823

[b191-tog-2008-099] Lee SB Huang K Palmer R Truong VB Herzlinger D Kolquist KA Wong J Paulding C Yoon SK Gerald W Oliner JD Haber DA 1999 The Wilms tumor suppressor WT1 encodes a transcriptional activator of amphiregulin Cell 98 663 73 10490105 10.1016/s0092-8674(00)80053-7

[b192-tog-2008-099] Lee YN Nechushtan H Figov N Razin E 2004 The function of lysyl-tRNA synthetase and Ap4A as signaling regulators of MITF activity in FcepsilonRI-activated mast cells Immunity 20 145 51 14975237 10.1016/s1074-7613(04)00020-2

[b193-tog-2008-099] Lee YN Razin E 2005 Nonconventional involvement of LysRS in the molecular mechanism of USF2 transcriptional activity in FcepsilonRI-activated mast cells Mol. Cell Biol 25 8904 12 16199869 10.1128/MCB.25.20.8904-8912.2005 PMC1265770

[b194-tog-2008-099] Leffert HL Sell S 1974 Alpha1-fetoprotein biosynthesis during the growth cycle of differentiated fetal rat hepatocytes in primary monolayer culture J. Cell Biol 61 823 9 4134464 10.1083/jcb.61.3.823 PMC2109317

[b195-tog-2008-099] Leissring MA Farris W Wu X Christodoulou DC Haigis MC Guarente L Selkoe DJ 2004 Alternative translation initiation generates a novel isoform of insulin-degrading enzyme targeted to mitochondria Biochem. J 383 439 46 15285718 10.1042/BJ20041081 PMC1133736

[b196-tog-2008-099] Lesk AM 2001 Introduction to Protein Architecture Oxford University Press Oxford, New York

[b197-tog-2008-099] Levay PF Viljoen M 1995 Lactoferrin: a general review Haematologica 80 252 67 7672721

[b198-tog-2008-099] Levitin F Baruch A Weiss M Stiegman K Hartmann ML Yoeli-Lerner M Ziv R Zrihan-Licht S Shina S Gat A Lifschitz B Simha M Stadler Y Cholostoy A Gil B Greaves D Keydar I Zaretsky J Smorodinsky N Wreschner DH 2005a A novel protein derived from the MUC1 gene by alternative splicing and frameshifting J. Biol. Chem 280 10655 63 15623537 10.1074/jbc.M406943200

[b199-tog-2008-099] Levitin F Stern O Weiss M Gil-Henn C Ziv R Prokocimer Z Smorodinsky NI Rubinstein DB Wreschner DH 2005b The MUC1 SEA module is a self-cleaving domain J. Biol. Chem 280 33374 86 15987679 10.1074/jbc.M506047200

[b200-tog-2008-099] Lewis BP Green RE Brenner SE 2003 Evidence for the widespread coupling of alternative splicing and nonsense-mediated mRNA decay in humans Proc. Natl. Acad. Sci. U.S.A 100 189 92 12502788 10.1073/pnas.0136770100 PMC140922

[b201-tog-2008-099] Lewis UJ Sinha YN Lewis GP 2000 Structure and properties of members of the hGH family: a review Endocr. J 47 Suppl S1 8 10890174 10.1507/endocrj.47.supplmarch_s1

[b202-tog-2008-099] Li MS Li PF Yang FY He SP Du GG Li G 2002 The intracellular mechanism of alpha-fetoprotein promoting the proliferation of NIH 3T3 cells Cell Res 12 151 6 12118941 10.1038/sj.cr.7290121

[b203-tog-2008-099] Li QJ Yao M Wong W Parpura V Martins-Green M 2004 The N- and C-terminal peptides of hIL8/CXCL8 are ligands for hCXCR1 and hCXCR2 Faseb J 18 776 8 14766805 10.1096/fj.02-1175fje

[b204-tog-2008-099] Li Y Bharti A Chen D Gong J Kufe D 1998 Interaction of glycogen synthase kinase 3beta with the DF3/MUC1 carcinoma-associated antigen and beta-catenin Mol. Cell Biol 18 7216 24 9819408 10.1128/mcb.18.12.7216 PMC109303

[b205-tog-2008-099] Li Y Chen W Ren J Yu WH Li Q Yoshida K Kufe D 2003a DF3/MUC1 signaling in multiple myeloma cells is regulated by interleukin-7 Cancer Biol. Ther 2 187 93 12750561 10.4161/cbt.2.2.282

[b206-tog-2008-099] Li Y Kuwahara H Ren J Wen G Kufe D 2001a The c-Src tyrosine kinase regulates signaling of the human DF3/MUC1 carcinoma-associated antigen with GSK3 beta and beta-catenin J. Biol. Chem 276 6061 4 11152665 10.1074/jbc.C000754200

[b207-tog-2008-099] Li Y Ren J Yu W Li Q Kuwahara H Yin L Carraway KL3rd Kufe D 2001b The epidermal growth factor receptor regulates interaction of the human DF3/MUC1 carcinoma antigen with c-Src and beta-catenin J. Biol. Chem 276 35239 42 11483589 10.1074/jbc.C100359200

[b208-tog-2008-099] Li Y Yu WH Ren J Chen W Huang L Kharbanda S Loda M Kufe D 2003b Heregulin targets gamma-catenin to the nucleolus by a mechanism dependent on the DF3/MUC1 oncoprotein Mol. Cancer Res 1 765 75 12939402

[b209-tog-2008-099] Ligtenberg MJ Kruijshaar L Buijs F van Meijer M Litvinov SV Hilkens J 1992 Cell.-associated episialin is a complex containing two proteins derived from a common precursor J. Biol. Chem 267 6171 7 1556125

[b210-tog-2008-099] Linsley PS Kallestad JC Horn D 1988 Biosynthesis of high molecular weight breast carcinoma associated mucin glycoproteins J. Biol. Chem 263 8390 7 3131343

[b211-tog-2008-099] Liou J Kim ML Heo WD Jones JT Myers JW Ferrell JEJr Meyer T 2005 STIM is a Ca2+ sensor essential for Ca2+-store-depletion-triggered Ca2+ influx Curr. Biol 15 1235 41 16005298 10.1016/j.cub.2005.05.055 PMC3186072

[b212-tog-2008-099] Litvinov SV Hilkens J 1993 The epithelial sialomucin, episialin, is sialylated during recycling J. Biol. Chem 268 21364 71 8407976

[b213-tog-2008-099] Loeb DM Korz D Katsnelson M Burwell EA Friedman AD Sukumar S 2002 Cyclin E is a target of WT1 transcriptional repression J. Biol. Chem 277 19627 32 11919196 10.1074/jbc.M201336200

[b214-tog-2008-099] Loftfield RB Vanderjagt D 1972 The frequency of errors in protein biosynthesis Biochem. J 128 1353 6 4643706 10.1042/bj1281353 PMC1174024

[b215-tog-2008-099] Lopez-Lastra M Rivas A Barria MI 2005 Protein synthesis in eukaryotes: the growing biological relevance of cap-independent translation initiation Biol. Res 38 121 46 16238092 10.4067/s0716-97602005000200003

[b216-tog-2008-099] Lu PD Harding HP Ron D 2004 Translation reinitiation at alternative open reading frames regulates gene expression in an integrated stress response J. Cell Biol 167 27 33 15479734 10.1083/jcb.200408003 PMC2172506

[b217-tog-2008-099] Lubas WA Frank DW Krause M Hanover JA 1997 O-Linked GlcNAc transferase is a conserved nucleocytoplasmic protein containing tetratricopeptide repeats J. Biol. Chem 272 9316 24 9083068 10.1074/jbc.272.14.9316

[b218-tog-2008-099] Lykke-Andersen J Shu MD Steitz JA 2001 Communication of the position of exon-exon junctions to the mRNA surveillance machinery by the protein RNPS1 Science 293 1836 9 11546874 10.1126/science.1062786

[b219-tog-2008-099] Ma S Hill KE Caprioli RM Burk RF 2002 Mass spectrometric characterization of full-length rat selenoprotein P and three isoforms shortened at the C terminus. Evidence that three UGA codons in the mRNA open reading frame have alternative functions of specifying selenocysteine insertion or translation termination J. Biol. Chem 277 12749 54 11821412 10.1074/jbc.M111462200

[b220-tog-2008-099] Mackenzie SA 2005 Plant organellar protein targeting: a traffic plan still under construction Trends Cell Biol 15 548 54 16143534 10.1016/j.tcb.2005.08.007

[b221-tog-2008-099] Maderazo AB He F Mangus DA Jacobson A 2000 Upf1p control of nonsense mRNA translation is regulated by Nmd2p and Upf3p Mol. Cell Biol 20 4591 603 10848586 10.1128/mcb.20.13.4591-4603.2000 PMC85857

[b222-tog-2008-099] Makarova KS Aravind L Koonin EV 1999 A superfamily of archaeal, bacterial, and eukaryotic proteins homologous to animal transglutaminases Protein Sci 8 1714 9 10452618 10.1110/ps.8.8.1714 PMC2144420

[b223-tog-2008-099] Manch-Citron JN Dey A Schneider R Nguyen NY 1999 The translational hop junction and the 5′ transcriptional start site for the Prevotella loescheii adhesin encoded by plaA Curr. Microbiol 38 22 6 9841777 10.1007/pl00006766

[b224-tog-2008-099] Mann DM Romm E Migliorini M 1994 Delineation of the glycosaminoglycan-binding site in the human inflammatory response protein lactoferrin J. Biol. Chem 269 23661 7 8089135

[b225-tog-2008-099] Manning G Plowman GD Hunter T Sudarsanam S 2002a Evolution of protein kinase signaling from yeast to man Trends Biochem. Sci 27 514 20 12368087 10.1016/s0968-0004(02)02179-5

[b226-tog-2008-099] Manning G Whyte DB Martinez R Hunter T Sudarsanam S 2002b The protein kinase complement of the human genome Science 298 1912 34 12471243 10.1126/science.1075762

[b227-tog-2008-099] Mariller C Benaissa M Hardiville S Breton M Pradelle G Mazurier J Pierce A 2007 Human delta-lactoferrin is a transcription factor that enhances Skp1 (S-phase kinase-associated protein) gene expression Febs J 274 2038 53 17371504 10.1111/j.1742-4658.2007.05747.x

[b228-tog-2008-099] Marino M Ascenzi P Acconcia F 2006 S-palmitoylation modulates estrogen receptor alpha localization and functions Steroids 71 298 303 16274718 10.1016/j.steroids.2005.09.011

[b229-tog-2008-099] Martinoia E Klein M Geisler M Bovet L Forestier C Kolukisaoglu U Muller-Rober B Schulz B 2002 Multifunctionality of plant ABC transporters—more than just detoxifiers Planta 214 345 55 11855639 10.1007/s004250100661

[b230-tog-2008-099] Matsufuji S Matsufuji T Miyazaki Y Murakami Y Atkins JF Gesteland RF Hayashi S 1995 Autoregulatory frameshifting in decoding mammalian ornithine decarboxylase antizyme Cell 80 51 60 7813017 10.1016/0092-8674(95)90450-6 PMC7133313

[b231-tog-2008-099] McCaughan KK Brown CM Dalphin ME Berry MJ Tate WP 1995 Translational termination efficiency in mammals is influenced by the base following the stop codon Proc. Natl. Acad. Sci. U.S.A 92 5431 5 7777525 10.1073/pnas.92.12.5431 PMC41708

[b232-tog-2008-099] McGrath JP Varshavsky A 1989 The yeast STE6 gene encodes a homologue of the mammalian multidrug resistance P-glycoprotein Nature 340 400 4 2569166 10.1038/340400a0

[b233-tog-2008-099] McNally D Fares MA 2007 In silico identification of functional divergence between the multiple groEL gene paralogs in Chlamydiae BMC Evol. Biol 7 81 17519003 10.1186/1471-2148-7-81 PMC1892554

[b234-tog-2008-099] Meador WE Means AR Quiocho FA 1992 Target enzyme recognition by calmodulin: 2.4 A structure of a calmodulin-peptide complex Science 257 1251 5 1519061 10.1126/science.1519061

[b235-tog-2008-099] Medina L Grove K Haltiwanger RS 1998 SV40 large T antigen is modified with O-linked N.-acetylglucosamine but not with other forms of glycosylation Glycobiology 8 383 91 9499386 10.1093/glycob/8.4.383

[b236-tog-2008-099] Medzihradszky KF Kannicht C 2002 Characterization of site-specific glycosylation Posttranslational modifications of proteins 101 102 Humana Press

[b237-tog-2008-099] Milligan G Parenti M Magee AI 1995 The dynamic role of palmitoylation in signal transduction Trends Biochem. Sci 20 181 7 7610481 10.1016/s0968-0004(00)89004-0

[b238-tog-2008-099] Milligan SR Khan O Nash M 1998 Competitive binding of xenobiotic oestrogens to rat alpha-fetoprotein and to sex steroid binding proteins in human and rainbow trout (Oncorhynchus mykiss) plasma Gen. Comp. Endocrinol 112 89 95 9748407 10.1006/gcen.1998.7146

[b239-tog-2008-099] Min W Jones DH 1994 In vitro splicing of concanavalin A is catalyzed by asparaginyl endopeptidase Nat. Struct. Biol 1 502 4 7664074 10.1038/nsb0894-502

[b240-tog-2008-099] Miranda LR Duval M Doherty H Seaman MS Posner MR Cavacini LA 2007 The neutralization properties of a HIV-specific antibody are markedly altered by glycosylation events outside the antigen-binding domain J. Immunol 178 7132 8 17513762 10.4049/jimmunol.178.11.7132

[b241-tog-2008-099] Mironov AA Fickett JW Gelfand MS 1999 Frequent alternative splicing of human genes Genome. Res 9 1288 93 10613851 10.1101/gr.9.12.1288 PMC310997

[b242-tog-2008-099] Missler M Fernandez-Chacon R Sudhof TC 1998 The making of neurexins J. Neurochem 71 1339 47 9751164 10.1046/j.1471-4159.1998.71041339.x

[b243-tog-2008-099] Missler M Sudhof TC 1998 Neurexins: three genes and 1001 products Trends Genet 14 20 6 9448462 10.1016/S0168-9525(97)01324-3

[b244-tog-2008-099] Mizejewski GJ 1993 An apparent dimerization motif in the third domain of alpha-fetoprotein: molecular mimicry of the steroid/thyroid nuclear receptor superfamily Bioessays 15 427 32 7689318 10.1002/bies.950150610

[b245-tog-2008-099] Mizejewski GJ Vonnegut M Jacobson HI 1983 Estradiol-activated alpha-fetoprotein suppresses the uterotropic response to estrogens Proc. Natl. Acad. Sci. U.S.A 80 2733 7 6189129 10.1073/pnas.80.9.2733 PMC393902

[b246-tog-2008-099] Mosley AL Lakshmanan J Aryal BK Ozcan S 2003 Glucose-mediated phosphorylation converts the transcription factor Rgt1 from a repressor to an activator J. Biol. Chem 278 10322 7 12527758 10.1074/jbc.M212802200

[b247-tog-2008-099] Mostaqul Huq MD Gupta P Wei LN 2008 Post-translational modifications of nuclear co-repressor RIP140: a therapeutic target for metabolic diseases Curr. Med. Chem 15 386 92 18288993 10.2174/092986708783497382

[b248-tog-2008-099] Murai N Murakami Y Matsufuji S 2003 Identification of nuclear export signals in antizyme-1 J. Biol. Chem 278 44791 8 12941943 10.1074/jbc.M308059200

[b249-tog-2008-099] Murakami Y Matsufuji S Kameji T Hayashi S Igarashi K Tamura T Tanaka K Ichihara A 1992 Ornithine decarboxylase is degraded by the 26S proteasome without ubiquitination Nature 360 597 9 1334232 10.1038/360597a0

[b250-tog-2008-099] Nadolski MJ Linder ME 2007 Protein lipidation Febs J 274 5202 10 17892486 10.1111/j.1742-4658.2007.06056.x

[b251-tog-2008-099] Nagai M Becker JL Deutsch HF 1982 The fatty acid levels of rat alpha-fetoprotein derived from fetuses, pregnancy and hepatoma sera Oncodev. Biol. Med 3 343 50 6183647

[b252-tog-2008-099] Nagradova NK 2004 Protein folding in the cell: on the mechanisms of its acceleration Biochemistry (Mosc) 69 830 43 15377262 10.1023/b:biry.0000040214.43943.9a

[b253-tog-2008-099] Namy O Duchateau-Nguyen G Rousset JP 2002 Translational readthrough of the PDE2 stop codon modulates cAMP levels in Sac-charomyces cerevisiae Mol. Microbiol 43 641 52 11929521 10.1046/j.1365-2958.2002.02770.x

[b254-tog-2008-099] Namy O Rousset JP Napthine S Brierley I 2004 Reprogrammed genetic decoding in cellular gene expression Mol. Cell 13 157 68 14759362 10.1016/s1097-2765(04)00031-0

[b255-tog-2008-099] Naval J Villacampa MJ Goguel AF Uriel J 1985 Cell-type-specific receptors for alpha-fetoprotein in a mouse T-lymphoma cell line Proc. Natl. Acad. Sci. U.S.A 82 3301 5 2582410 10.1073/pnas.82.10.3301 PMC397763

[b256-tog-2008-099] Nemansky M Thotakura NR Lyons CD Ye S Reinhold BB Reinhold VN Blithe DL 1998 Developmental changes in the glycosylation of glycoprotein hormone free alpha subunit during pregnancy J. Biol. Chem 273 12068 76 9575149 10.1074/jbc.273.20.12068

[b257-tog-2008-099] Nishi S Matsue H Yoshida H Yamaoto R Sakai M 1991 Localization of the estrogen-binding site of alpha-fetoprotein in the chimeric human-rat proteins Proc. Natl. Acad. Sci. U.S.A 88 3102 5 1707533 10.1073/pnas.88.8.3102 PMC51393

[b258-tog-2008-099] Noren CJ Wang J Perler FB 2000 Dissecting the Chemistry of Protein Splicing and Its Applications Angew Chem. Int. Ed. Engl 39 450 66 10671234

[b259-tog-2008-099] Nott A Le Hir H Moore MJ 2004 Splicing enhances translation in mammalian cells: an additional function of the exon junction complex Genes Dev 18 210 22 14752011 10.1101/gad.1163204 PMC324426

[b260-tog-2008-099] Nykjaer A Willnow TE 2002 The low-density lipoprotein receptor gene family: a cellular Swiss army knife? Trends Cell Biol 12 273 80 12074887 10.1016/s0962-8924(02)02282-1

[b261-tog-2008-099] Ohkura K Hori H 2000 Analyses of insulin-potentiating fragments of human growth hormone by computative simulation; essential unit for insulin-involved biological responses Bioorg Med. Chem 8 1733 40 10976521 10.1016/s0968-0896(00)00105-x

[b262-tog-2008-099] Ohno S 1970 Evolution by gene duplication Springer-Verlag Berlin

[b263-tog-2008-099] Osterhout JL Waheed AA Hiol A Ward RJ Davey PC Nini L Wang J Milligan G Jones TL Druey KM 2003 Palmitoylation regulates regulator of G-protein signaling (RGS) 16 function. II. Palmitoylation of a cysteine residue in the RGS box is critical for RGS16 GTPase accelerating activity and regulation of Gi-coupled signalling J. Biol. Chem 278 19309 16 12642592 10.1074/jbc.M210124200

[b264-tog-2008-099] Otani A Slike BM Dorrell MI Hood J Kinder K Ewalt KL Cheresh D Schimmel P Friedlander M 2002 A fragment of human TrpRS as a potent antagonist of ocular angiogenesis Proc. Natl. Acad. Sci. U.S.A 99 178 83 11773625 10.1073/pnas.012601899 PMC117535

[b265-tog-2008-099] Packham G Brimmell M Cleveland JL 1997 Mammalian cells express two differently localized Bag-1 isoforms generated by alternative translation initiation Biochem. J 328 Pt 3 807 13 9396724 10.1042/bj3280807 PMC1218990

[b266-tog-2008-099] Paraoanu LE Layer PG 2004 Mouse acetylcholinesterase interacts in yeast with the extracellular matrix component laminin-1beta FEBS Lett 576 161 4 15474030 10.1016/j.febslet.2004.08.078

[b267-tog-2008-099] Paulus H 2001 Inteins as enzymes Bioorg Chem 29 119 29 11437387 10.1006/bioo.2001.1203

[b268-tog-2008-099] Pawson T Gish GD 1992 SH2 and SH3 domains: from structure to function Cell 71 359 62 1423600 10.1016/0092-8674(92)90504-6

[b269-tog-2008-099] Pawson T Nash P 2003 Assembly of cell regulatory systems through protein interaction domains Science 300 445 52 12702867 10.1126/science.1083653

[b270-tog-2008-099] Perler FB 2005 Protein splicing mechanisms and applications IUBMB. Life 57 469 76 16081367 10.1080/15216540500163343

[b271-tog-2008-099] Perler FB Davis EO Dean GE Gimble FS Jack WE Neff N Noren CJ Thorner J Belfort M 1994 Protein splicing elements: inteins and exteins—a definition of terms and recommended nomenclature Nucleic Acids Res 22 1125 7 8165123 10.1093/nar/22.7.1125 PMC523631

[b272-tog-2008-099] Persechini A Kretsinger RH 1988 The central helix of calmodulin functions as a flexible tether J. Biol. Chem 263 12175 8 3137220

[b273-tog-2008-099] Pestova TV Hellen CU 1999 Ribosome recruitment and scanning: what’s new? Trends Biochem. Sci 24 85 7 10203752 10.1016/s0968-0004(99)01356-0

[b274-tog-2008-099] Pham CT MacIvor DM Hug BA Heusel JW Ley TJ 1996 Long-range disruption of gene expression by a selectable marker cassette Proc. Natl. Acad. Sci. U.S.A 93 13090 5 8917549 10.1073/pnas.93.23.13090 PMC24051

[b275-tog-2008-099] Pietrokovski S 2001 Intein spread and extinction in evolution Trends Genet 17 465 72 11485819 10.1016/s0168-9525(01)02365-4

[b276-tog-2008-099] Plant EP Jacobs KL Harger JW Meskauskas A Jacobs JL Baxter JL Petrov AN Dinman JD 2003 The 9-A solution: how mRNA pseudoknots promote efficient programmed-1 ribosomal frameshifting Rna 9 168 74 12554858 10.1261/rna.2132503 PMC1237042

[b277-tog-2008-099] Plateau P Blanquet S 1982 Zinc-dependent synthesis of various dinucleoside 5′, 5′-P1,P3-Tri- or 5″, 5′-P1,P4-tetraphosphates by Escherichia coli lysyl-tRNA synthetase Biochemistry 21 5273 9 6756470 10.1021/bi00264a024

[b278-tog-2008-099] Porras P Padilla CA Krayl M Voos W Barcena JA 2006 One single in-frame AUG codon is responsible for a diversity of subcellular localizations of glutaredoxin 2 in Saccharomyces cerevisiae J. Biol. Chem 281 16551 62 16606613 10.1074/jbc.M600790200

[b279-tog-2008-099] Qiao F Bowie JU 2005 The many faces of SAM Sci. STKE 2005 re7 15928333 10.1126/stke.2862005re7

[b280-tog-2008-099] Quinton TM Kim S Dangelmaier C Dorsam RT Jin J Daniel JL Kunapuli SP 2002 Protein kinase C- and calcium-regulated pathways independently synergize with Gi pathways in agonist-induced fibrinogen receptor activation Biochem. J 368 535 43 12215172 10.1042/BJ20020226 PMC1223015

[b281-tog-2008-099] Rammensee HG 2004 Immunology: protein surgery Nature 427 203 4 14724617 10.1038/427203a

[b282-tog-2008-099] Regev-Rudzki N Karniely S Ben-Haim NN Pines O 2005 Yeast aconitase in two locations and two metabolic pathways: seeing small amounts is believing Mol. Biol. Cell 16 4163 71 15975908 10.1091/mbc.E04-11-1028 PMC1196327

[b283-tog-2008-099] Regev-Rudzki N Pines O 2007 Eclipsed distribution: a phenomenon of dual targeting of protein and its significance Bioessays 29 772 82 17621655 10.1002/bies.20609

[b284-tog-2008-099] Rehfeld JF Goetze JP 2003 The posttranslational phase of gene expression: new possibilities in molecular diagnosis Curr. Mol. Med 3 25 38 12558072 10.2174/1566524033361717

[b285-tog-2008-099] Ren J Li Y Kufe D 2002 Protein kinase C delta regulates function of the DF3/MUC1 carcinoma antigen in beta-catenin signaling J. Biol. Chem 277 17616 22 11877440 10.1074/jbc.M200436200

[b286-tog-2008-099] Resh MD 1999 Fatty acylation of proteins: new insights into membrane targeting of myristoylated and palmitoylated proteins Biochim. Biophys. Acta 1451 1 16 10446384 10.1016/s0167-4889(99)00075-0

[b287-tog-2008-099] Revell PA Grossman WJ Thomas DA Cao X Behl R Ratner JA Lu ZH Ley TJ 2005 Granzyme B. and the downstream granzymes C and/or F are important for cytotoxic lymphocyte functions J. Immunol 174 2124 31 15699143 10.4049/jimmunol.174.4.2124

[b288-tog-2008-099] Richardson JS 1981 The anatomy and taxonomy of protein structure Adv. Protein Chem 34 167 339 7020376 10.1016/s0065-3233(08)60520-3

[b289-tog-2008-099] Roberts AW 2005 G-CSF: a key regulator of neutrophil production, but that’s not all! Growth Factors 23 33 41 16019425 10.1080/08977190500055836

[b290-tog-2008-099] Robinson DN Cooley L 1997 Examination of the function of two kelch proteins generated by stop codon suppression Development 124 1405 17 9118811 10.1242/dev.124.7.1405

[b291-tog-2008-099] Rochette-Egly C 2003 Nuclear receptors: integration of multiple signalling pathways through phosphorylation Cell Signal 15 355 66 12618210 10.1016/s0898-6568(02)00115-8

[b292-tog-2008-099] Rom E Kahana C 1994 Polyamines regulate the expression of ornithine decarboxylase antizyme in vitro by inducing ribosomal frame-shifting Proc. Natl. Acad. Sci. U.S.A 91 3959 63 8171019 10.1073/pnas.91.9.3959 PMC43702

[b293-tog-2008-099] Roos J DiGregorio PJ Yeromin AV Ohlsen K Lioudyno M Zhang S Safrina O Kozak JA Wagner SL Cahalan MD Velicelebi G Stauderman KA 2005 STIM1, an essential and conserved component of store-operated Ca2+ channel function J. Cell Biol 169 435 45 15866891 10.1083/jcb.200502019 PMC2171946

[b294-tog-2008-099] Roos MD Su K Baker JR Kudlow JE 1997 O glycosylation of an Sp1-derived peptide blocks known Sp1 protein interactions Mol. Cell. Biol 17 6472 80 9343410 10.1128/mcb.17.11.6472 PMC232500

[b295-tog-2008-099] Roquemore EP Chevrier MR Cotter RJ Hart GW 1996 Dynamic O-GlcNAcylation of the small heat shock protein alpha B-crystallin Biochemistry 35 3578 86 8639509 10.1021/bi951918j

[b296-tog-2008-099] Routier FH Davies MJ Bergemann K Hounsell EF 1997 The glycosylation pattern of humanized IgGI antibody (D1.3) expressed in CHO cells Glycoconj J 14 201 7 9111137 10.1023/a:1018589704981

[b297-tog-2008-099] Ruiz-Gutierrez V Moreno R Moreda W Copado MA Rodriguez-Burgos A 2001 Detection of squalene in alpha-fetoprotein and fetal serum albumin from bovine J. Protein Chem 20 19 23 11330344 10.1023/a:1011096702910

[b298-tog-2008-099] Ruoslahti E Estes T Seppala M 1979 Binding of bilirubin by bovine and human alpha-fetoprotein Biochim. Biophys. Acta 578 511 9 90526 10.1016/0005-2795(79)90181-8

[b299-tog-2008-099] Sampath P Mazumder B Seshadri V Gerber CA Chavatte L Kinter M Ting SM Dignam JD Kim S Driscoll DM Fox PL 2004 Noncanonical function of glutamyl-prolyl-tRNA synthetase: gene-specific silencing of translation Cell 119 195 208 15479637 10.1016/j.cell.2004.09.030

[b300-tog-2008-099] Santonico E Castagnoli L Cesareni G 2005 Methods to reveal domain networks Drug Discov. Today 10 1111 7 16182196 10.1016/S1359-6446(05)03513-0

[b301-tog-2008-099] Sass E Blachinsky E Karniely S Pines O 2001 Mitochondrial and cytosolic isoforms of yeast fumarase are derivatives of a single translation product and have identical amino termini J. Biol. Chem 276 46111 7 11585823 10.1074/jbc.M106061200

[b302-tog-2008-099] Schartner JM Fathman CG Seroogy CM 2007 Preservation of self: an overview of E3 ubiquitin ligases and T cell tolerance Semin. Immunol 19 188 96 17403607 10.1016/j.smim.2007.02.010

[b303-tog-2008-099] Scheiffele P Fan J Choih J Fetter R Serafini T 2000 Neuroligin expressed in nonneuronal cells triggers presynaptic development in contacting axons Cell 101 657 69 10892652 10.1016/s0092-8674(00)80877-6

[b304-tog-2008-099] Schmucker D Clemens JC Shu H Worby CA Xiao J Muda M Dixon JE Zipursky SL 2000 Drosophila Dscam is an axon guidance receptor exhibiting extraordinary molecular diversity Cell 101 671 84 10892653 10.1016/s0092-8674(00)80878-8

[b305-tog-2008-099] Sheeley DM Merrill BM Taylor LC 1997 Characterization of monoclonal antibody glycosylation: comparison of expression systems and identification of terminal alpha-linked galactose Anal Biochem 247 102 10 9126378 10.1006/abio.1997.2036

[b306-tog-2008-099] Shyr CR Collins LL Mu XM Platt KA Chang C 2002 Spermatogenesis and testis development are normal in mice lacking testicular orphan nuclear receptor 2 Mol. Cell. Biol 22 4661 6 12052874 10.1128/MCB.22.13.4661-4666.2002 PMC133912

[b307-tog-2008-099] Silvius JR 2002 Mechanisms of Ras protein targeting in mammalian cells J. Membr. Biol 190 83 92 12474073 10.1007/s00232-002-1026-4

[b308-tog-2008-099] Singh PK Hollingsworth MA 2006 Cell. surface-associated mucins in signal transduction Trends Cell. Biol 16 467 76 16904320 10.1016/j.tcb.2006.07.006

[b309-tog-2008-099] Smorodinsky N Weiss M Hartmann ML Baruch A Harness E Yaakobovitz M Keydar I Wreschner DH 1996 Detection of a secreted MUC1/SEC protein by MUC1 isoform specific monoclonal antibodies Biochem. Biophys. Res. Commun 228 115 21 8912645 10.1006/bbrc.1996.1625

[b310-tog-2008-099] Son KN Park J Chung CK Chung DK Yu DY Lee KK Kim J 2002 Human lactoferrin activates transcription of IL-1beta gene in mammalian cells Biochem. Biophys. Res. Commun 290 236 41 11779159 10.1006/bbrc.2001.6181

[b311-tog-2008-099] Sorg C 1989 Macrophage-derived cell regulatory factors Cytokines Karger Basel

[b312-tog-2008-099] Southworth MW Amaya K Evans TC Xu MQ Perler FB 1999 Purification of proteins fused to either the amino or carboxy terminus of the Mycobacterium xenopi gyrase A intein Biotechniques 27 110 4 116 118 20 10407673 10.2144/99271st04

[b313-tog-2008-099] Stahl G Ben Salem S Li Z McCarty G Raman A Shah M Farabaugh PJ 2001 Programmed +1 translational frameshifting in the yeast Saccharomyces cerevisiae results from disruption of translational error correction Cold Spring Harb Symp. Quant. Biol 66 249 58 12762026 10.1101/sqb.2001.66.249

[b314-tog-2008-099] Stansfield I Jones KM Herbert P Lewendon A Shaw WV Tuite MF 1998 Missense translation errors in Saccharomyces cerevisiae J. Mol. Biol 282 13 24 9733638 10.1006/jmbi.1998.1976

[b315-tog-2008-099] Starokadomskyy P 2007 Protein splicing Molecular Biology 41 278 93

[b316-tog-2008-099] Stein I Itin A Einat P Skaliter R Grossman Z Keshet E 1998 Translation of vascular endothelial growth factor mRNA by internal ribosome entry: implications for translation under hypoxia Mol. Cell Biol 18 3112 9 9584152 10.1128/mcb.18.6.3112 PMC108893

[b317-tog-2008-099] Steneberg P Samakovlis C 2001 A novel stop codon readthrough mechanism produces functional Headcase protein in Drosophila trachea EMBO Rep 2 593 7 11463742 10.1093/embo-reports/kve128 PMC1083942

[b318-tog-2008-099] Strehl A Munnix IC Kuijpers MJ van der Meijden PE Cosemans JM Feijge MA Nieswandt B Heemskerk JW 2007 Dual role of platelet protein kinase C in thrombus formation: stimulation of pro-aggregatory and suppression of procoagulant activity in platelets J. Biol. Chem 282 7046 55 17210570 10.1074/jbc.M611367200

[b319-tog-2008-099] Stuehr DJ Cho HJ Kwon NS Weise MF Nathan CF 1991 Purification and characterization of the cytokine-induced macrophage nitric oxide synthase: an FAD- and FMN-containing flavoprotein Proc. Natl. Acad. Sci. U.S.A 88 7773 7 1715579 10.1073/pnas.88.17.7773 PMC52385

[b320-tog-2008-099] Su Z Wang J Yu J Huang X Gu X 2006 Evolution of alternative splicing after gene duplication Genome. Res 16 182 9 16365379 10.1101/gr.4197006 PMC1361713

[b321-tog-2008-099] Sugita S Khvochtev M Sudhof TC 1999 Neurexins are functional alpha-latrotoxin receptors Neuron 22 489 96 10197529 10.1016/s0896-6273(00)80704-7

[b322-tog-2008-099] Sukenaga Y Ishida K Takeda T Takagi K 1987 cDNA sequence coding for human glutathione peroxidase Nucleic Acids Res 15 7178 3658677 10.1093/nar/15.17.7178 PMC306203

[b323-tog-2008-099] Sunderland PA West CE Waterworth WM Bray CM 2006 An evolutionarily conserved translation initiation mechanism regulates nuclear or mitochondrial targeting of DNA ligase 1 in Arabidopsis thaliana Plant J 47 356 67 16790030 10.1111/j.1365-313X.2006.02791.x

[b324-tog-2008-099] Suzuki T Park H Lennarz WJ 2002 Cytoplasmic peptide: N-glycanase (PNGase) in eukaryotic cells: occurrence, primary structure, and potential functions Faseb J 16 635 41 11978727 10.1096/fj.01-0889rev

[b325-tog-2008-099] Suzuki T Tanabe K Hara I Taniguchi N Colavita A 2007 Dual enzymatic properties of the cytoplasmic peptide: N-glycanase in C. elegans Biochem. Biophys. Res. Commun 358 837 41 17509531 10.1016/j.bbrc.2007.04.199

[b326-tog-2008-099] Suzuki Y Zeng CQ Alpert E 1992 Isolation and partial characterization of a specific alpha-fetoprotein receptor on human monocytes J. Clin. Invest 90 1530 6 1383274 10.1172/JCI116021 PMC443200

[b327-tog-2008-099] Suzuki YA Lonnerdal B 2002 Characterization of mammalian receptors for lactoferrin Biochem. Cell Biol 80 75 80 11908646 10.1139/o01-228

[b328-tog-2008-099] Szczypka MS Wemmie JA Moye-Rowley WS Thiele DJ 1994 A yeast metal resistance protein similar to human cystic fibrosis transmembrane conductance regulator (CFTR.) and multidrug resistance-associated protein J. Biol. Chem 269 22853 7 7521334

[b329-tog-2008-099] Tagashira M Iijima H Isogai Y Hori M Takamatsu S Fujibayashi Y Yoshizawa-Kumagaye K Isaka S Nakajima K Yamamoto T Teshima T Toma K 2001 Site-dependent effect of O-glycosylation on the conformation and biological activity of calcitonin Biochemistry 40 11090 5 11551206 10.1021/bi010306y

[b330-tog-2008-099] Tanabe O Katsuoka F Campbell AD Song W Yamamoto M Tanimoto K Engel JD 2002 An embryonic/fetal beta-type globin gene repressor contains a nuclear receptor TR2/TR4 heterodimer Embo J 21 3434 42 12093744 10.1093/emboj/cdf340 PMC126089

[b331-tog-2008-099] Tatarinov YS Terentiev AA Moldogazieva NT Tagirova AK 1991 Human alpha-fetoprotein and its purification by chromatography on immobilized estrogens Tumour. Biol 12 125 30 1712508 10.1159/000217697

[b332-tog-2008-099] Tate WP Mansell JB Mannering SA Irvine JH Major LL Wilson DN 1999 UGA: a dual signal for ‘stop’ and for recoding in protein synthesis Biochemistry (Mosc) 64 1342 53 10648957

[b333-tog-2008-099] Tatebayashi K Tanaka K Yang HY Yamamoto K Matsushita Y Tomida T Imai M Saito H 2007 Transmembrane mucins Hkr1 and Msb2 are putative osmosensors in the SHO1 branch of yeast HOG pathway Embo J 26 3521 33 17627274 10.1038/sj.emboj.7601796 PMC1949007

[b334-tog-2008-099] Terentiev AA Moldogazieva NT 2006 Structural and functional mapping of alpha-fetoprotein Biochemistry (Mosc) 71 120 32 16489915 10.1134/s0006297906020027

[b335-tog-2008-099] Theil EC Eisenstein RS 2000 Combinatorial mRNA regulation: iron regulatory proteins and iso-iron-responsive elements (Iso-IREs) J. Biol. Chem 275 40659 62 11062250 10.1074/jbc.R000019200

[b336-tog-2008-099] Torres CR Hart GW 1984 Topography and polypeptide distribution of terminal N.-acetylglucosamine residues on the surfaces of intact lymphocytes. Evidence for O-linked GlcNAc J. Biol. Chem 259 3308 17 6421821

[b337-tog-2008-099] Torres JM Darracq N Uriel J 1992 Membrane proteins from lymphoblastoid cells showing cross-affinity for alpha-fetoprotein and albumin. Isolation and characterization Biochim. Biophys. Acta 1159 60 6 1382611 10.1016/0167-4838(92)90075-o

[b338-tog-2008-099] Torres JM Laborda J Naval J Darracq N Calvo M Mishal Z Uriel J 1989 Expression of alpha-fetoprotein receptors by human T-lymphocytes during blastic transformation Mol. Immunol 26 851 7 2481232 10.1016/0161-5890(89)90141-7

[b339-tog-2008-099] Touriol C Bornes S Bonnal S Audigier S Prats H Prats AC Vagner S 2003 Generation of protein isoform diversity by alternative initiation of translation at non-AUG codons Biol. Cell 95 169 78 12867081 10.1016/s0248-4900(03)00033-9

[b340-tog-2008-099] Treitel MA Kuchin S Carlson M 1998 Snf1 protein kinase regulates phosphorylation of the Mig1 repressor in Saccharomyces cerevisiae Mol. Cell Biol 18 6273 80 9774644 10.1128/mcb.18.11.6273 PMC109214

[b341-tog-2008-099] Trewhella J 1992 The solution structures of calmodulin and its complexes with synthetic peptides based on target enzyme binding domains Cell Calcium 13 377 90 1505003 10.1016/0143-4160(92)90051-s

[b342-tog-2008-099] Tsarfaty I Hareuveni M Horev J Zaretsky J Weiss M Jeltsch JM Garnier JM Lathe R Keydar I Wreschner DH 1990 Isolation and characterization of an expressed hypervariable gene coding for a breast-cancer-associated antigen Gene 93 313 8 1688329 10.1016/0378-1119(90)90242-j

[b343-tog-2008-099] Tsukuda M Asaoka Y Sekiguchi K Kikkawa U Nishizuka Y 1988 Properties of protein kinase C subspecies in human platelets Biochem. Biophys. Res. Commun 155 1387 95 3140809 10.1016/s0006-291x(88)81295-6

[b344-tog-2008-099] Ubersax JA Ferrell JE Jr 2007 Mechanisms of specificity in protein phosphorylation Nat. Rev. Mol. Cell Biol 8 530 41 17585314 10.1038/nrm2203

[b345-tog-2008-099] Ullrich B Ushkaryov YA Sudhof TC 1995 Cartography of neurexins: more than 1000 isoforms generated by alternative splicing and expressed in distinct subsets of neurons Neuron 14 497 507 7695896 10.1016/0896-6273(95)90306-2

[b346-tog-2008-099] Urner F Sakkas D 2003 Protein phosphorylation in mammalian spermatozoa Reproduction 125 17 26 12622692 10.1530/rep.0.1250017

[b347-tog-2008-099] Vigneron N Stroobant V Chapiro J Ooms A Degiovanni G Morel S van der Bruggen P Boon T Van den Eynde BJ 2004 An antigenic peptide produced by peptide splicing in the proteasome Science 304 587 90 15001714 10.1126/science.1095522

[b348-tog-2008-099] Vincent TS Fraylick JE McGuffie EM Olson JC 1999 ADP-ribosylation of oncogenic Ras proteins by pseudomonas aeruginosa exoenzyme S in vivo Mol. Microbiol 32 1054 64 10361307 10.1046/j.1365-2958.1999.01420.x

[b349-tog-2008-099] von der Haar T Tuite MF 2007 Regulated translational bypass of stop codons in yeast Trends Microbiol 15 78 86 17187982 10.1016/j.tim.2006.12.002

[b350-tog-2008-099] Wakasugi K Schimmel P 1999 Two distinct cytokines released from a human aminoacyl-tRNA synthetase Science 284 147 51 10102815 10.1126/science.284.5411.147

[b351-tog-2008-099] Walden WE Selezneva AI Dupuy J Volbeda A Fontecilla-Camps JC Theil EC Volz K 2006 Structure of dual function iron regulatory protein 1 complexed with ferritin IRE-RNA Science 314 1903 8 17185597 10.1126/science.1133116

[b352-tog-2008-099] Wang W Czaplinski K Rao Y Peltz SW 2001 The role of Upf proteins in modulating the translation read-through of nonsense-containing transcripts Embo J 20 880 90 11179232 10.1093/emboj/20.4.880 PMC145432

[b353-tog-2008-099] Ward PP Uribe-Luna S Conneely OM 2002 Lactoferrin and host defense Biochem. Cell Biol 80 95 102 11908649 10.1139/o01-214

[b354-tog-2008-099] Weiss M Zaretsky J Zimlichman R Smorodinsky N Dion AS Keydar I Wreschner DH 1991 Expression of a gene coding for breast tumor-associated antigen in thyroid papillary carcinoma Cancer Lett 58 125 30 2049778 10.1016/0304-3835(91)90034-f

[b355-tog-2008-099] Wells L Hart GW 2003 O-GlcNAc turns twenty: functional implications for post-translational modification of nuclear and cytosolic proteins with a sugar FEBS Lett 546 154 8 12829252 10.1016/s0014-5793(03)00641-0

[b356-tog-2008-099] Wen Y Caffrey TC Wheelock MJ Johnson KR Hollingsworth MA 2003 Nuclear association of the cytoplasmic tail of MUC1 and beta-catenin J. Biol. Chem 278 38029 39 12832415 10.1074/jbc.M304333200

[b357-tog-2008-099] Wiegand HL Lu S Cullen BR 2003 Exon junction complexes mediate the enhancing effect of splicing on mRNA expression Proc. Natl. Acad. Sci. U.S.A 100 11327 32 12972633 10.1073/pnas.1934877100 PMC208756

[b358-tog-2008-099] Wilchek M Miron T 1974 Polymers coupled to agarose as stable and high capacity spacers Methods Enzymol 34 72 6 4449485 10.1016/s0076-6879(74)34008-6

[b359-tog-2008-099] Wilkinson MF 2005 A new function for nonsense-mediated mRNA-decay factors Trends Genet 21 143 8 15734573 10.1016/j.tig.2005.01.007

[b360-tog-2008-099] Williams CJ Wreschner DH Tanaka A Tsarfaty I Keydar I Dion AS 1990 Multiple protein forms of the human breast tumor-associated epithelial membrane antigen (EMA) are generated by alternative splicing and induced by hormonal stimulation Biochem. Biophys. Res. Commun 170 1331 8 2202302 10.1016/0006-291x(90)90540-4

[b361-tog-2008-099] Williams I Richardson J Starkey A Stansfield I 2004 Genome-wide prediction of stop codon readthrough during translation in the yeast Saccharomyces cerevisiae Nucleic Acids Res 32 6605 16 15602002 10.1093/nar/gkh1004 PMC545446

[b362-tog-2008-099] Williams RT Manji SS Parker NJ Hancock MS Van Stekelenburg L Eid JP Senior PV Kazenwadel JS Shandala T Saint R Smith PJ Dziadek MA 2001 Identification and characterization of the STIM (stromal interaction molecule) gene family: coding for a novel class of transmembrane proteins Biochem. J 357 673 85 11463338 10.1042/0264-6021:3570673 PMC1221997

[b363-tog-2008-099] Willnow TE Sheng Z Ishibashi S Herz J 1994 Inhibition of hepatic chylomicron remnant uptake by gene transfer of a receptor antagonist Science 264 1471 4 7515194 10.1126/science.7515194

[b364-tog-2008-099] Wilson RB Renault G Jacquet M Tatchell K 1993 The pde2 gene of Saccharomyces cerevisiae is allelic to rca1 and encodes a phosphodiesterase which protects the cell from extracellular cAMP FEBS Lett 325 191 5 8391474 10.1016/0014-5793(93)81071-7

[b365-tog-2008-099] Wilson RB Tatchell K 1988 SRA5 encodes the low-Km cyclic AMP phosphodiesterase of Saccharomyces cerevisiae Mol. Cell Biol 8 505 10 2827010 10.1128/mcb.8.1.505-510.1988 PMC363163

[b366-tog-2008-099] Wood P 2001 Growth hormone: its measurement and the need for assay harmonization Ann. Clin. Biochem 38 471 82 11587125 10.1177/000456320103800504

[b367-tog-2008-099] Wreschner DH Hareuveni M Tsarfaty I Smorodinsky N Horev J Zaretsky J Kotkes P Weiss M Lathe R Dion A 1990 Human epithelial tumor antigen cDNA sequences. Differential splicing may generate multiple protein forms Eur. J. Biochem 189 463 73 2351132 10.1111/j.1432-1033.1990.tb15511.x

[b368-tog-2008-099] Wreschner DH McGuckin MA Williams SJ Baruch A Yoeli M Ziv R Okun L Zaretsky J Smorodinsky N Keydar I Neophytou P Stacey M Lin HH Gordon S 2002 Generation of ligand-receptor alliances by SEA module-mediated cleavage of membrane-associated mucin proteins Protein Sci 11 698 706 11847293 10.1110/ps.16502 PMC2373471

[b369-tog-2008-099] Wreschner DH Zrihan-Licht S Baruch A Sagiv D Hartman ML Smorodinsky N Keydar I 1994 Does a novel form of the breast cancer marker protein, MUC1, act as a receptor molecule that modulates signal transduction? Adv Exp. Med. Biol 353 17 26 7985536 10.1007/978-1-4615-2443-4_3

[b370-tog-2008-099] Wriggers W Chakravarty S Jennings PA 2005 Control of protein functional dynamics by peptide linkers Biopolymers 80 736 46 15880774 10.1002/bip.20291

[b371-tog-2008-099] Wu Y Matthews CR 2002 A cis-prolyl peptide bond isomerization dominates the folding of the alpha subunit of Trp synthase, a TIM barrel protein J. Mol. Biol 322 7 13 12215410 10.1016/s0022-2836(02)00737-4

[b372-tog-2008-099] Yannay-Cohen N Razin E 2006 Translation and transcription: the dual functionality of LysRS in mast cells Mol. Cells 22 127 32 17085962

[b373-tog-2008-099] Yap KL Kim J Truong K Sherman M Yuan T Ikura M 2000 Calmodulin target database J. Struct. Funct. Genomics 1 8 14 12836676 10.1023/a:1011320027914

[b374-tog-2008-099] Yook SH Oltvai ZN Barabasi AL 2004 Functional and topological characterization of protein interaction networks Proteomics 4 928 42 15048975 10.1002/pmic.200300636

[b375-tog-2008-099] Yoshinaka Y Katoh I Copeland TD Oroszlan S 1985 Murine leukemia virus protease is encoded by the gag-pol gene and is synthesized through suppression of an amber termination codon Proc. Natl. Acad. Sci. U.S.A 82 1618 22 3885215 10.1073/pnas.82.6.1618 PMC397323

[b376-tog-2008-099] Yueh A Schneider RJ 1996 Selective translation initiation by ribosome jumping in adenovirus-infected and heat-shocked cells Genes Dev 10 1557 67 8666238 10.1101/gad.10.12.1557

[b377-tog-2008-099] Zamecnik PC Stephenson ML Janeway CM Randerath K 1966 Enzymatic synthesis of diadenosine tetraphosphate and diadenosine triphosphate with a purified lysyl-sRNA synthetase Biochem. Biophys. Res. Commun 24 91 7 5338216 10.1016/0006-291x(66)90415-3

[b378-tog-2008-099] Zaretsky JZ Barnea I Aylon Y Gorivodsky M Wreschner DH Keydar I 2006 MUC1 gene overexpressed in breast cancer: structure and transcriptional activity of the MUC1 promoter and role of estrogen receptor alpha (ERalpha) in regulation of the MUC1 gene expression Mol. Cancer 5 57 17083744 10.1186/1476-4598-5-57 PMC1636664

[b379-tog-2008-099] Zaretsky JZ Sarid R Aylon Y Mittelman LA Wreschner DH Keydar I 1999 Analysis of the promoter of the MUC1 gene over-expressed in breast cancer FEBS Lett 461 189 95 10567695 10.1016/s0014-5793(99)01452-0

[b380-tog-2008-099] Zaretsky JZ Weiss M Tsarfaty I Hareuveni M Wreschner DH Keydar I 1990 Expression of genes coding for pS2, c-erbB2, estrogen receptor and the H23 breast tumor-associated antigen. A comparative analysis in breast cancer FEBS Lett 265 46 50 2194831 10.1016/0014-5793(90)80880-r

[b381-tog-2008-099] Zhang SL Yu Y Roos J Kozak JA Deerinck TJ Ellisman MH Stauderman KA Cahalan MD 2005 STIM1 is a Ca2+ sensor that activates CRAC channels and migrates from the Ca2+ store to the plasma membrane Nature 437 902 5 16208375 10.1038/nature04147 PMC1618826

[b382-tog-2008-099] Zipursky SL Wojtowicz WM Hattori D 2006 Got diversity? Wiring the fly brain with Dscam Trends Biochem. Sci 31 581 8 16919957 10.1016/j.tibs.2006.08.003

[b383-tog-2008-099] Zrihan-Licht S Vos HL Baruch A Elroy-Stein O Sagiv D Keydar I Hilkens J Wreschner DH 1994 Characterization and molecular cloning of a novel MUC1 protein, devoid of tandem repeats, expressed in human breast cancer tissue Eur. J. Biochem 224 787 95 7925397 10.1111/j.1432-1033.1994.00787.x

